# A microprotein atlas of the human frontal cortex in Alzheimer’s disease

**DOI:** 10.1038/s43587-026-01207-x

**Published:** 2026-09-14

**Authors:** Brendan Miller, Eduardo Vieira de Souza, Calvin Lau, Joan M. Vaughan, Victor J. Pai, Servando Giraldez, Andréa Rocha, Jolene K. Diedrich, Clodagh C. O’Shea, David A. Bennett, Alan Saghatelian

**Affiliations:** 1 Clayton Foundation Laboratories for Peptide Biology, The Salk Institute for Biological Studies, La Jolla, CA USA; 2 Molecular and Cell Biology Laboratory, The Salk Institute for Biological Studies, La Jolla, CA USA; 3 Department of Integrative Structural and Computational Biology, The Scripps Research Institute, La Jolla, CA USA; 4 Rush Alzheimer’s Disease Center, Rush University Medical Center, Chicago, IL USA

**Keywords:** Ageing, Alzheimer's disease, Gene expression

## Abstract

Understanding the molecular basis of neurodegeneration requires a comprehensive map of the genome’s protein-coding output. Although thousands of small open reading frames (ORFs) are translated in the human brain, proteomic evidence for their encoded microproteins (MPs) (≤150 amino acids (aa)) remains limited. Here, we present a brain MP atlas that integrates transcriptomics, mass spectrometry and deep-learning-predicted spectra across more than 600 postmortem frontal cortex samples with and without Alzheimer’s disease (AD). We identified 1,067 MPs absent from reviewed UniProtKB entries with high-confidence spectral support. A subset is differentially expressed in AD independently of the annotated main ORF at the same locus. A small ORF expressed by *MKKS* encodes a 63-amino-acid MP that is the locus’s predominant translation product and is downregulated in AD; its loss impairs microglial mitochondrial respiration, implicating it in microglial bioenergetics. This atlas expands the annotated brain proteome and provides a resource for studying MPs in aging and neurodegeneration.

## Abstract (web summary)

A new atlas identifies 1,067 unannotated microproteins in the human frontal cortex with strong spectral grades. A subset is differentially expressed in Alzheimer’s disease independently of their canonical genes, including an *MKKS*-derived microprotein required for normal microglial mitochondrial respiration.

## Main

The molecular mechanisms of healthy cognitive aging remain incompletely understood. Multi-omics studies of the postmortem human brain have provided key insights but depend on reference genome databases that do not represent small open reading frames (smORFs). These smORFs are genomic elements that, when transcribed and translated, can encode short polypeptides, often termed microproteins (MPs) (typically fewer than 100–150 aa)^1–3^. Because of their historical exclusion, the role of MPs and the genes that encode them remain largely unexplored in omics-based neurobiology research.

Approximately 10% of the entries in UniProtKB’s reviewed section (Swiss-Prot) are proteins shorter than 150 aa (ref. ^4^), but this fraction may be an underestimate^5^. Recently, the TransCODE consortium documented over 7,000 actively translated smORFs across human cell types, nearly a quarter with mass spectrometry (MS) evidence, termed ‘peptideins’ to denote expression evidence without an established biological function^6^. Should these peptideins be experimentally validated as functional proteins, they would nearly double the number of annotated entries shorter than 150 aa in the reviewed UniProt/Swiss-Prot database.

Collaborative efforts, such as the TransCODE consortium, aim to enhance the annotation of MPs by overcoming historical detection challenges. MPs have been difficult to detect because their short length yields few proteolytic peptides, limiting their identification using MS. In addition, since a large fraction of smORFs is embedded within or adjacent to annotated protein-coding ORFs, conventional RNA sequencing workflows cannot infer expression of smORFs alone. Recent advances in ribosome profiling, proteogenomics and MS-based technologies have begun to address these challenges.

Ribosome sequencing (Ribo-seq) enables the identification of thousands of translating smORFs by sequencing ribosome-protected fragments^7,8^. MS-based proteomics provides evidence that smORF translation indeed yields an MP^9–11^. Once MPs are detected, machine learning and computational approaches are often used to infer their biological function^12–14^. Characterized MP functions range from mitochondrial membrane components to secreted signaling peptides and immunopeptidome modulators^15^, positioning them within aging and neurodegeneration pathways. Despite their emerging functional relevance, a comprehensive brain MP atlas has not been built. Such a resource would provide an experimental entry point for investigating smORFs and their MPs in fundamental neurobiology and neurodegeneration.

To address this gap, we built a transcript-to-MP atlas integrating native transcriptomics with deep proteomics across more than 600 postmortem frontal cortex tissues with and without Alzheimer’s disease (AD). This atlas provides protein-level expression evidence in the form of tandem MS (MS/MS) spectra graded against deep-learning-predicted references. We report over 1,000 MPs absent from the reviewed Swiss-Prot catalog whose spectra closely match predicted spectra. One of these is an MP encoded by the *MKKS* locus that is significantly downregulated in AD, disrupts mitochondrial oxidative phosphorylation upon knockout (KO) and diverges in expression from the canonical MKKS ORF-encoded protein, a decoupling also seen in other MPs, replicated across cohorts and linked to RNA-binding protein (RBP) motifs near splice junctions. Data including MS/PROSIT plots, grades, expression profiles, sequences and formatted files are accessible via a browsable application, which can be used to filter sequences based on user-specific interests (https://huggingface.co/spaces/brmiller/brain-microprotein-atlas-app).

## Results

### Unique MS evidence of MPs in aged human brains

To identify MPs with unique MS evidence, we generated a custom dorsolateral prefrontal cortex (DLPFC)-derived protein search database from 12 native transcriptomes^16^. This database consists of all putative smORFs and their resulting MP sequences appended to the UniProt reference proteome (UP000005640). UniProt organizes entries into two curation tiers: Swiss-Prot (manually curated, experimentally supported) and TrEMBL (computationally annotated, awaiting review)^4^. We designated Swiss-Prot-matched sequences (≤150 aa) as reviewed and the combined set of TrEMBL-matched sequences plus transcriptome-reconstructed smORFs not annotated by Ensembl as unreviewed.

**Fig. 1:**
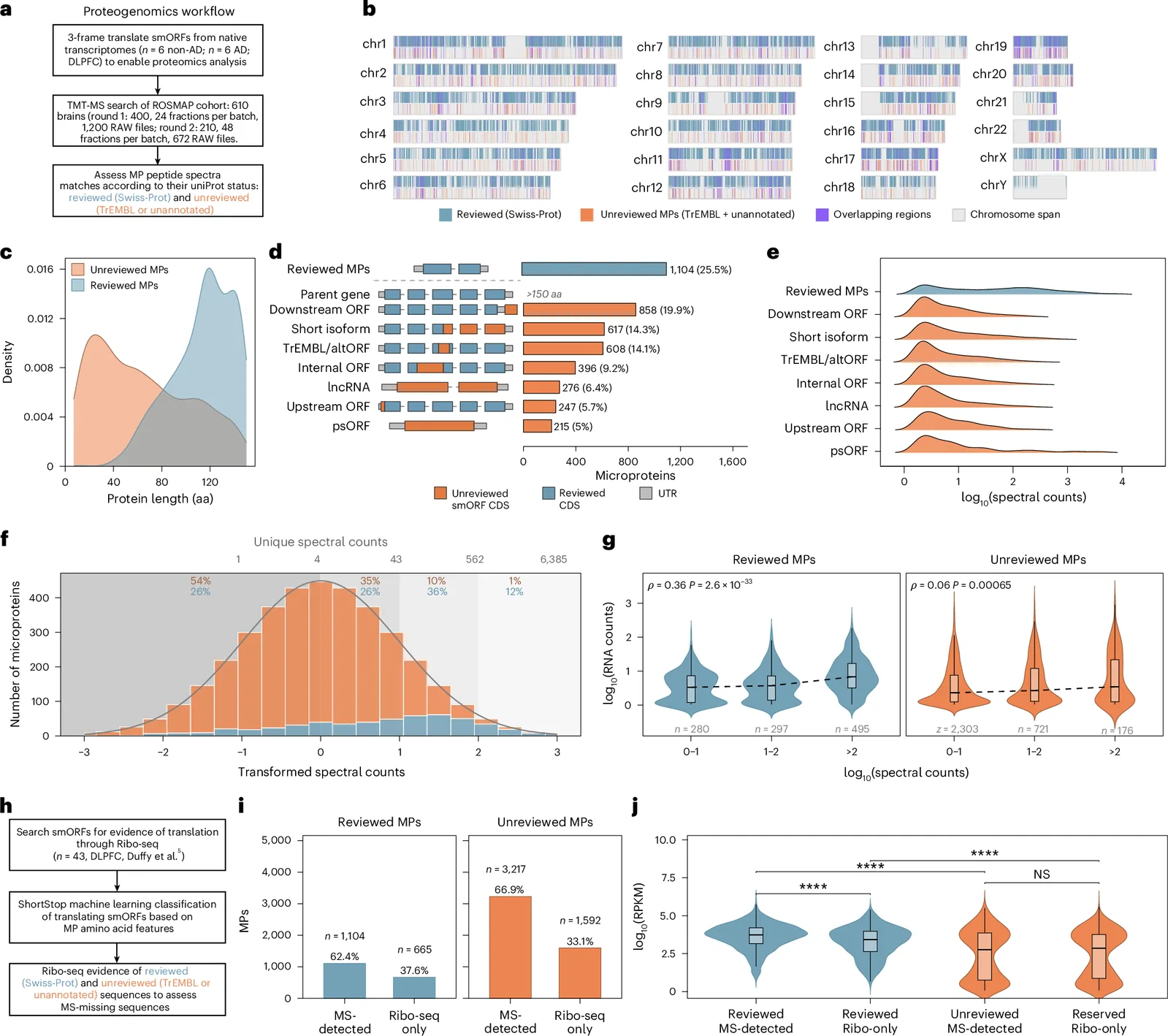
MS evidence of MPs in the aged human frontal cortex. Box plots (embedded within violins where shown): the center line represents the median; the box bounds represent the 25th and 75th percentiles; and the whiskers extend to the most extreme data points within 1.5× the interquartile range (defining the plotted minima and maxima). **a**, Long-read transcriptomes from 12 DLPFC samples (six AD/six non-AD; six females, six males) were assembled, three-frame translated and appended to the UniProt reference proteome for proteomics search studies. TMT-MS search cohort: 610 brains, biological replicates (round 1: *n* = 400, 24 fractions per batch, 1,200 RAW files; round 2: *n* = 210, 48 fractions per batch, 672 RAW files). **b**, Proteogenomic map of aged human DLPFC according to chromosome: reviewed (Swiss-Prot, teal) and unreviewed (TrEMBL + unannotated, orange) MPs; overlap, purple. **c**, Protein length density: reviewed ≤150 aa (*n* = 1,104) and unreviewed (*n* = 3,217) MPs with MS evidence in ROSMAP. **d**, smORF location and counts (*n* = 4,321 total). smORF types are shown according to their position relative to the main ORF of the annotated parental gene (for example, upstream ORF); definitions are given in the Methods. **e**, Spectral count distribution according to smORF type (*n* = 3,732), ridgeline, log_10_ scale. **f**, Total spectral counts (*n* = 3,732), reviewed/unreviewed stacked; percentage shown per transformed bin. **g**, RNA–protein correlation according to the number of spectral count bins (0–1, 1–2, >2). Protein abundance: TMT-MS spectral counts (*n* = 610 ROSMAP brains). RNA: counts per million (CPM) (*n* = 373 ROSMAP brains). Two-sided Spearman correlation, 95% confidence interval (CI) via Fisher’s *z*. Reviewed: *n* = 1,072, *ρ* = 0.356 (0.302–0.407), *P* = 2.61 × 10^−33^. Unreviewed: *n* = 3,200, *ρ* = 0.060 (0.026–0.095), *P* = 6.51 × 10^−4^. Correlation was done on razor peptides for Swiss-Prot; correlation was done on unique peptides for unreviewed. **h**, Ribo-seq (43 non-AD DLPFC brains) recovered MPs missed by MS; ShortStop classified translated smORFs according to aa physicochemical properties. **i**, MPs according to detection evidence (MS-detected versus Ribo-seq only), reviewed/unreviewed. **j**, Ribosome occupancy (log_10_ reads per kilobase per million mapped reads (RPKM)) according to detection evidence; *n* = 43 brains; *n* = 816/631/747/980 MPs with uniquely mappable reads (reviewed MS-detected/Ribo-only, unreviewed MS-detected/Ribo-only). Two-sided Wilcoxon rank-sum (four comparisons), effect size r_rb (rank-biserial correlation, −1 to 1): within reviewed, MS-detected versus Ribo-only, r_rb = −0.209, *P* = 8.58 × 10^−12 (****);^ within unreviewed, MS-detected versus Ribo-only, NS (*P* = 0.969); within MS-detected, reviewed versus unreviewed, r_rb = −0.411, *P* = 6.34 × 10^−45 (****);^ within Ribo-only, reviewed versus unreviewed, r_rb = −0.285, *P* = 4.68 × 10^−22 (****).^ Asterisks denote *P* < 0.0001. NS, not significant. Source data

We then used this search database to identify MPs in tandem mass tag (TMT)-MS data from 610 DLPFC samples from the Religious Orders Study (ROS) and the Memory and Aging Project (MAP) across the AD neuropathological spectrum. ROSMAP TMT-MS data are derived from 400 samples across 50 batches and 24 fractions (1,200 RAW files) and 210 samples across 14 batches and 48 fractions (672 raw files)^17^. Our analysis yielded 4,321 MPs passing protein identification with a false discovery rate (FDR) < 0.01, including 3,217 unreviewed and 1,104 reviewed (Fig. 1a–d and Supplementary Tables 1–3), each with at least one unique peptide-spectrum match (PSM).

The 3,217 unreviewed MPs map broadly across all autosomes, mostly encoded near or within genes producing a larger canonical protein (Fig. 1b), with a median aa length nearly half that of the reviewed MPs (median 58 aa versus 117; Fig. 1c). Their encoding smORFs are located upstream and downstream, within the coding sequence (CDS) of annotated ORFs, or are derived from transcripts annotated as noncoding. Downstream ORFs (dORFs) were the largest class (19.9% of all 4,321 MPs), followed by short isoforms (14.3%) and TrEMBL/altORFs (14.1%), with internal ORFs (iORFs) (9.2%), long noncoding RNA (lncRNA)-hosted smORFs (6.4%), upstream ORFs (uORFs) (5.7%) and pseudogene ORFs (psORFs) (5.0%) comprising the remainder (Fig. 1d).

MPs are historically challenging to detect using MS because of limited peptide recovery from short length and relatively low abundance^9^. Indeed, reviewed MPs generated roughly an order of magnitude more spectral counts per sequence than unreviewed MPs (Fig. 1e); 11% of unreviewed MPs exceeded mean abundance by more than one standard deviation, versus 48% of reviewed MPs (Fig. 1f, and Supplementary Table 4). Unreviewed MPs also showed substantially weaker protein–mRNA concordance than reviewed MPs (*ρ* = 0.06 versus *ρ* = 0.36; Fig. 1g).

Because MPs may fall below MS detection thresholds, we integrated Ribo-seq from 43 non-AD DLPFC samples with ShortStop, a machine learning framework that classifies translated smORFs resembling annotated UniProt/Swiss-Prot MPs^5,12^ (Fig. 1h). This identified 665 of 1,769 (37.6%) reviewed and 1,592 of 4,809 (33.1%) translated MPs lacking MS evidence in ROSMAP TMT-MS (Fig. 1i). Among MPs with unique ribosome coverage evidence, MS-detected reviewed entries showed higher ribosome occupancy than Ribo-seq-only reviewed entries (median log_10_ RPKM = 3.70 versus 3.38; *P* = 8.58 × 10^−12^) and unreviewed MPs (2.72; *P* = 6.34 × 10^−45^), indicating that MS identification is associated with higher unique Ribo-seq coverage for reviewed sequences with a functional annotation (Fig. 1j). Data-independent acquisition (DIA) MS across four samples recovered only modest additional sequences (35 reviewed, 30 unreviewed) (Supplementary Table 1), suggesting that limited recovery reflects abundance, not a technical limitation.

### Integrating Ribo-seq, MS and PROSIT provides an evidence framework for confidently identifying brain-expressed MPs

**Fig. 2:**
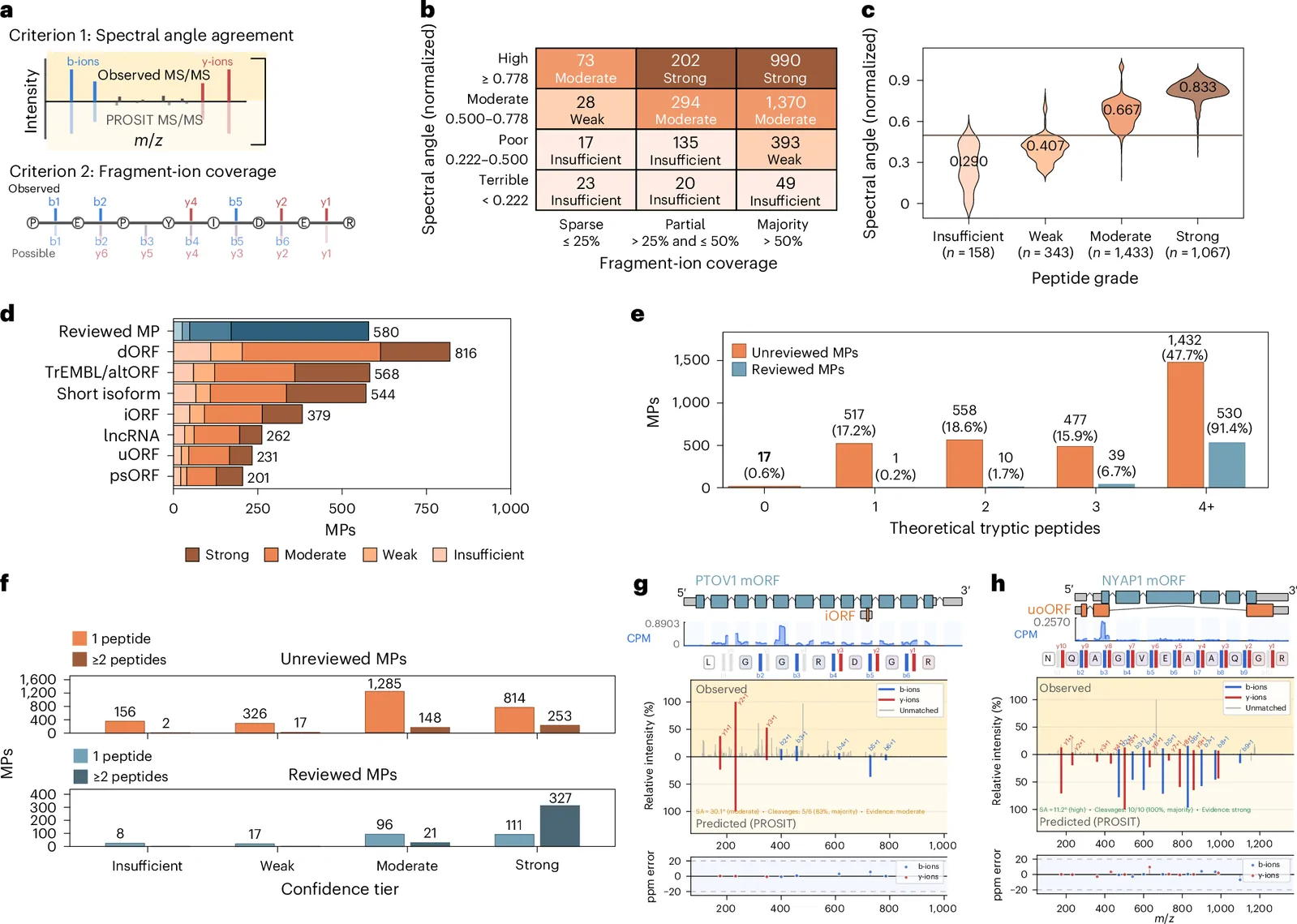
MP MS grades derived from PROSIT SA and fragment-ion coverage. **a**, Peptide grading criteria. Criterion 1: SA agreement between observed and PROSIT-predicted spectra. Criterion 2: fragment-ion coverage (percentage of b/y-ions observed). **b**, Peptide grading matrix (*n* = 3,594 peptides uniquely mapped to unreviewed MPs). Each cell reports the count of peptides in each combination of normalized SA bin (≥0.778/0.500–0.778/0.222–0.500/≤0.222) and fragment-ion coverage bin (sparse <25%/partial 25–50%/majority >50%). Cell labels indicate the assigned grade (strong/moderate/weak/insufficient). **c**, Per-protein SA distributions (median SA across the peptides of each unreviewed MP; *n* = 3,001), stratified according to confidence grade (insufficient *n* = 158, weak *n* = 343, moderate *n* = 1,433, strong *n* = 1,067). Descriptive distribution only; no statistical test was performed. **d**, MP peptide grade according to smORF type (stacked bar chart; *n* = 3,581 proteins total). Each protein is assigned its highest confidence uniquely mapped tryptic peptide grade. Strong/moderate/weak/insufficient tiers are shown for reviewed MPs (*n* = 580), dORFs (*n* = 816), TrEMBL/altORFs (*n* = 568), short isoforms (*n* = 544), iORFs (*n* = 379), lncRNA-ORFs (*n* = 262), uORFs (*n* = 231) and psORFs (*n* = 201). **e**, Theoretical tryptic unique peptide capacity for MPs amenable to PROSIT examination. The bar plots show the fraction of reviewed (*n* = 580) and unreviewed (*n* = 3,001) MPs expected to yield 0, 1, 2, 3 or ≥ 4 tryptic peptides based on the sequence. Descriptive distribution only. **f**, Observed unique tryptic peptide support according to confidence tier, split into 1-peptide and ≥2 tryptic peptide categories for unreviewed (*n* = 3,001) and reviewed (*n* = 580) MPs. **g**,**h**, Representative PROSIT mirror plots of an iORF from the *PTOV1* locus graded moderate (**g**) and a uoORF from the *NYAP1* locus (**h**) graded strong. The parent gene schematic and Ribo-seq track are shown above each spectrum (*y* axis, Ribo-seq CPM). Representative of a single identified spectrum. Source data

**Extended Data Fig. 1:**
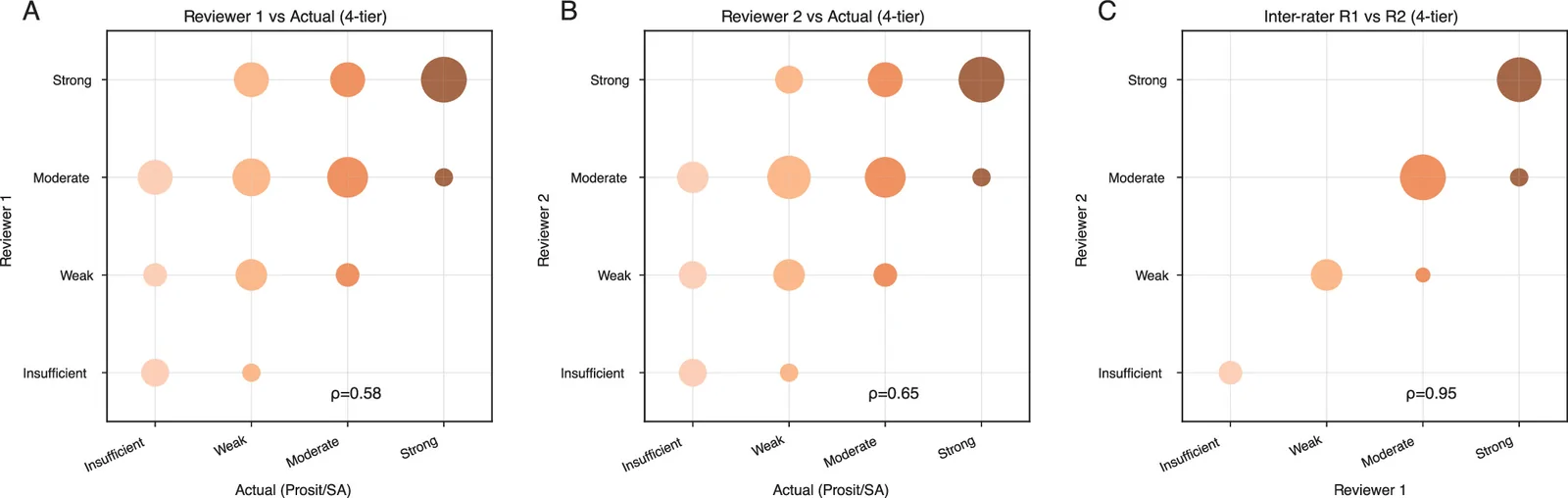
Manual blind grading of predicted spectral quality correlates with PROSIT-based scores. Balloon plots comparing manually assigned tiers against the PROSIT spectral-angle (SA)-derived tier for Reviewer 1 (**a**) and Reviewer 2 (**b**) with inter-rater agreement (**c**). Unit of study = individual spectrum; *n* = 50 spectra per panel. Bubble area is proportional to the number of spectra at each coordinate pair (maximum *n* = 50); bubble shade reflects the grading tier. Statistics: two-sided Spearman rank correlation. (**a**) Reviewer 1 versus SA tier: ρ = 0.584, P = 8.32 × 10^−6^. (**b**) Reviewer 2 versus SA tier: ρ = 0.647, P = 3.79 × 10^−7^. (**c**) inter-rater agreement (Reviewer 1 versus Reviewer 2): ρ = 0.955, P = 7.01 × 10^−27^. Source data

Large-scale proteomics studies are inherently susceptible to false positives^18^. Therefore, we assessed the spectral quality of the 3,217 unreviewed MPs that passed protein identification (FDR < 0.01) by using PROSIT, a deep learning framework predicting theoretical fragmentation spectra for comparison against observed spectra^19^. Each MP was assigned to one of four confidence tiers (strong, moderate, weak, insufficient) based on its combination of PROSIT-compared spectral angle (SA) score (0–1 scale, normalized) and fragment-ion coverage, with thresholds derived from previously reported MP grading criteria (Fig. 2a,b)^20^. This scoring system was cross-validated against two independent manual reviewers (Extended Data Fig. 1).

Of 3,001 unreviewed MPs amenable to PROSIT interrogation, 1,067 (35.6%) were strong, 1,433 (47.8%) moderate, 343 (11.4%) weak and 158 (5.3%) insufficient. Over 90% of MPs graded as strong had more than 50% fragment-ion coverage and a high normalized SA score over 0.78 (Fig. 2c). Similarly, over 95% of MPs graded as moderate had fragment-ion coverage over 50% but lacked the high SA concordance; nevertheless, these may reflect genuine peptides, warrant follow-up and should not be discarded. Overall, across all grades, MP detection was consistent for all smORF classes (Fig. 2d).

**Extended Data Fig. 2:**
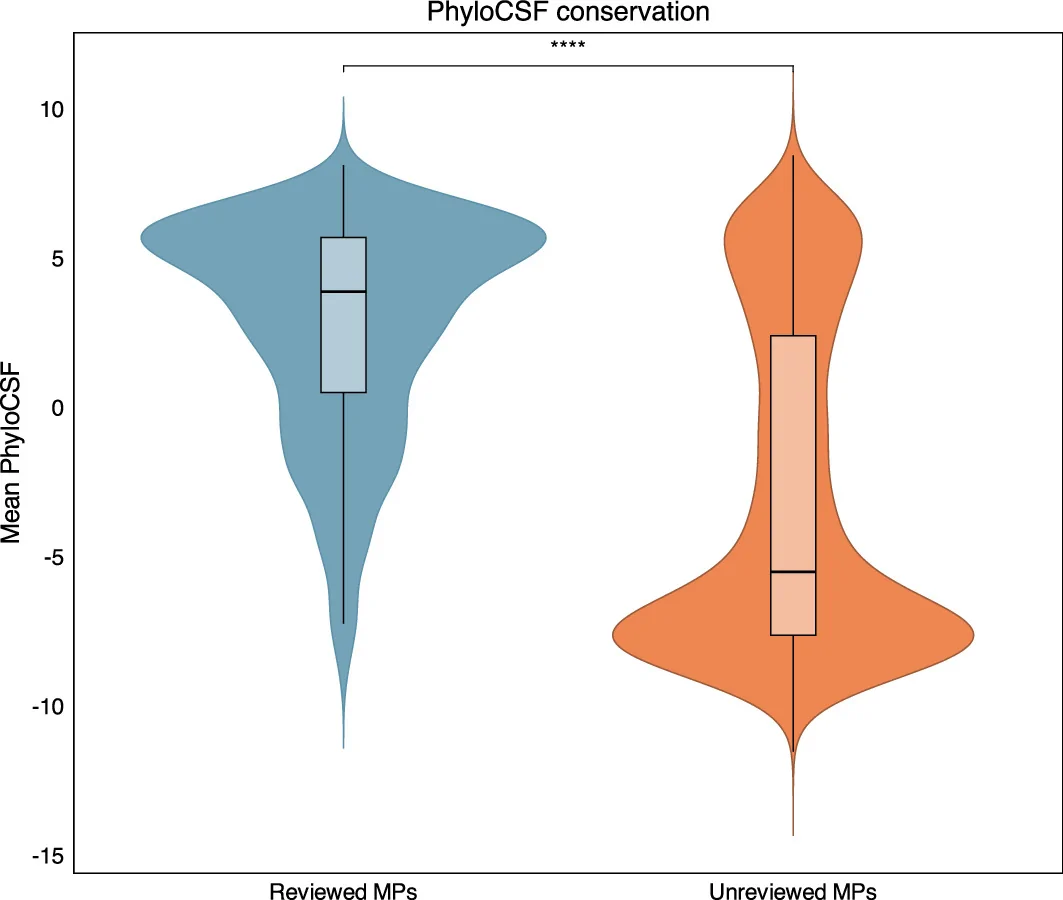
PhyloCSF scores of Reviewed (Swiss-Prot) versus Unreviewed (TrEMBL + Unannotated) microproteins. *n* = 1,530 Reviewed and *n* = 4,052 Unreviewed microproteins, spanning both MS-detected and Ribo-seq/ShortStop-detected, with a computed mean PhyloCSF score (biologically independent sequences). Violin plots show the score distribution with embedded box plots (center line, median; box bounds 25th and 75th percentiles; whiskers extend to the most extreme data points within 1.5× the interquartile range). Statistics: two-sided Wilcoxon rank-sum test; W = 4,864,549, P < 2.2 × 10^−16^ (below the floating-point floor reported by R, asterisks denote ****). Reviewed microproteins show significantly higher PhyloCSF scores (median 3.90 vs −5.49), consistent with stronger evolutionary conservation. Source data

Conventional proteomics typically requires two unique tryptic peptides per protein for confident identification^21^, a threshold most MPs cannot meet given their short length (Fig. 2e). Conservation-based approaches such as PhyloCSF offer an alternative but are also poorly suited to MPs because many that have been documented are evolutionarily young^22^; accordingly, nearly three-quarters of MS-evidenced MPs in our dataset showed negative PhyloCSF scores (Extended Data Fig. 2). Consequently, relying on peptide count or conservation alone would exclude many bona fide MPs.

Our MS-based gradient system and ribosome profiling integration workflow helps recover MPs that peptide-count or conservation filters alone would exclude. Among 1,067 strong-grade unreviewed MPs, 814 had just one observed tryptic PSM (Fig. 2f), but these can be further assessed using ribosome profiling (Fig. 2g–h and Supplementary Tables 3–5). For example, an MP encoded by an upstream overlapping ORF (uoORF) within *NYAP1* illustrates this: despite a negative PhyloCSF score (−3.4) and only one detectable tryptic peptide, it showed clear ribosome occupancy and a strong PROSIT grade (Fig. 2h).

### Differential MP abundance in AD using TMT-MS quantification

**Fig. 3:**
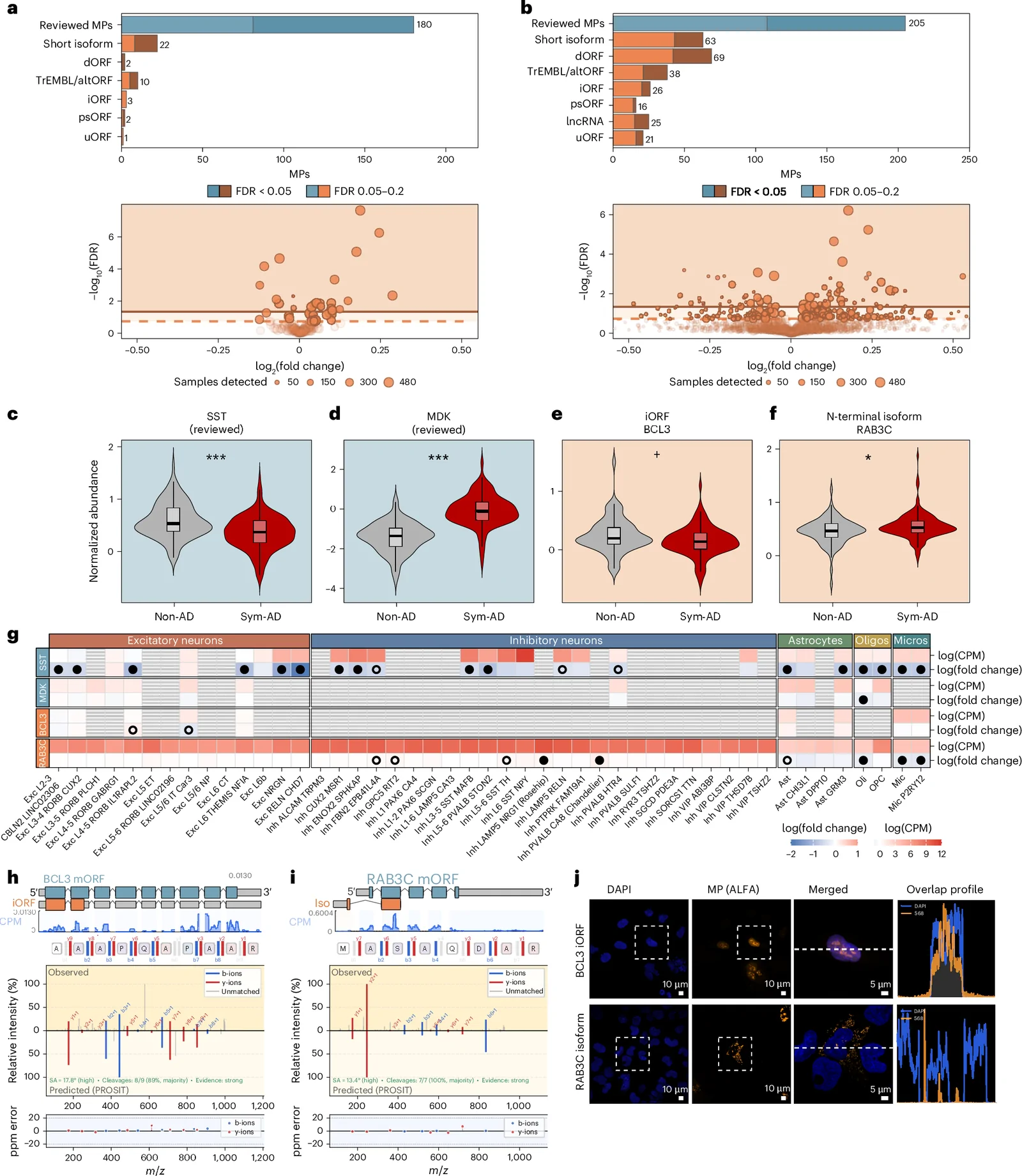
Differential abundance of MPs in AD using TMT quantitation. Symbols denote Benjamini–Hochberg FDR: + < 0.2; * < 0.05; ** < 0.01; *** < 0.001. **a**,**b**, Differentially abundant MPs, sym-AD versus non-AD, according to smORF type, including reviewed MPs. The bars split into high-confidence (FDR < 0.05, light orange and light teal) and exploratory (FDR = 0.05–0.2, dark orange and dark teal) tiers. Groups: non-AD (*n* = 111) versus sym-AD (*n* = 187). Two-sided Student’s *t*-test, Benjamini–Hochberg FDR, corrected within each stringency model. **a**, Stringent: detected in more than 50% of donors (*n* = 580 MPs). **b**, Relaxed: detected in one or more donors per condition (*n* = 3,179 MPs). **c**–**f**, Normalized abundance, non-AD versus sym-AD, for two reviewed and two unreviewed sequences: SST (**c**), MDK (reviewed) (**d**), BCL3-iORF (**e**) and RAB3C N-terminal isoform (unreviewed) (**f**). Donor *n* (non-AD/sym-AD): SST 111/187; MDK 109/178; BCL3-iORF 69/100; RAB3C 87/162. Violin plots with embedded box plots (center line, median; box, 25th–75th percentiles; whiskers, most extreme values within 1.5× the interquartile range (IQR)). Two-sided Student’s *t*-test, Benjamini–Hochberg FDR, stringent model (≥ 50% detection); values are *t*-statistic, log_2_(fold change) (95% CI), Cohen’s *d*, exact *P* and FDR. **c**, SST: *t*_(296)_ = −6.01, log_2_(fold change) = −0.236 (−0.314 to −0.159), *d* = −0.720, *P* = 5.59 × 10^−9^, FDR = 3.08 × 10^−7^ (***). **d**, MDK: *t*_(285)_ = 14.07, log_2_(fold change) = 1.342 (1.155 to 1.530), *d* = 1.712, *P* = 1.63 × 10^−34^, FDR = 6.05 × 10^−31^ (***). **e**, BCL3-iORF: *t*_(167)_ = −2.40, log_2_(fold change) = −0.103 (−0.188 to −0.018), *d* = −0.375, *P* = 0.0177, FDR = 0.0595 (+). **f**, RAB3C: *t*_(247)_ = 3.01, log_2_(fold change) = 0.097 (0.034 to 0.161), *d* = 0.401, *P* = 0.00284, FDR = 0.0148 (*). **g**, Heatmap: smORF-containing genes mapped to snRNA-seq cell types using summary statistics (Mathys et al.^24^), showing log(CPM) and log(fold change) for SST, MDK, BCL3 and RAB3C across neuronal and glial subtypes. On the log(fold change) row, filled circles mark significant global AD pathology association (summary statistics adjusted *P* < 0.05); open circles mark trend-level association (0.05–0.1); unmarked cells reached neither threshold. log(CPM) carries no significance marking. **h**,**i**, PROSIT mirror plots, both strong quality, representative of one spectrum per peptide: BCL3-iORF (peptide AAAPQAPAAR) (**h**) and RAB3C N-terminal isoform (peptide MASAQDAR) (**i**). **j**, Confocal microscopy of HMC3 microglia overexpressing C-terminal ALFA-tagged BCL3-iORF and RAB3C N-terminal isoform. Representative of three independent transfections. 4′,6-diamidino-2-phenylindole (DAPI) (blue) and ALFA (orange); BCL3-iORF localizes to the nucleus. Source data

**Extended Data Fig. 3:**
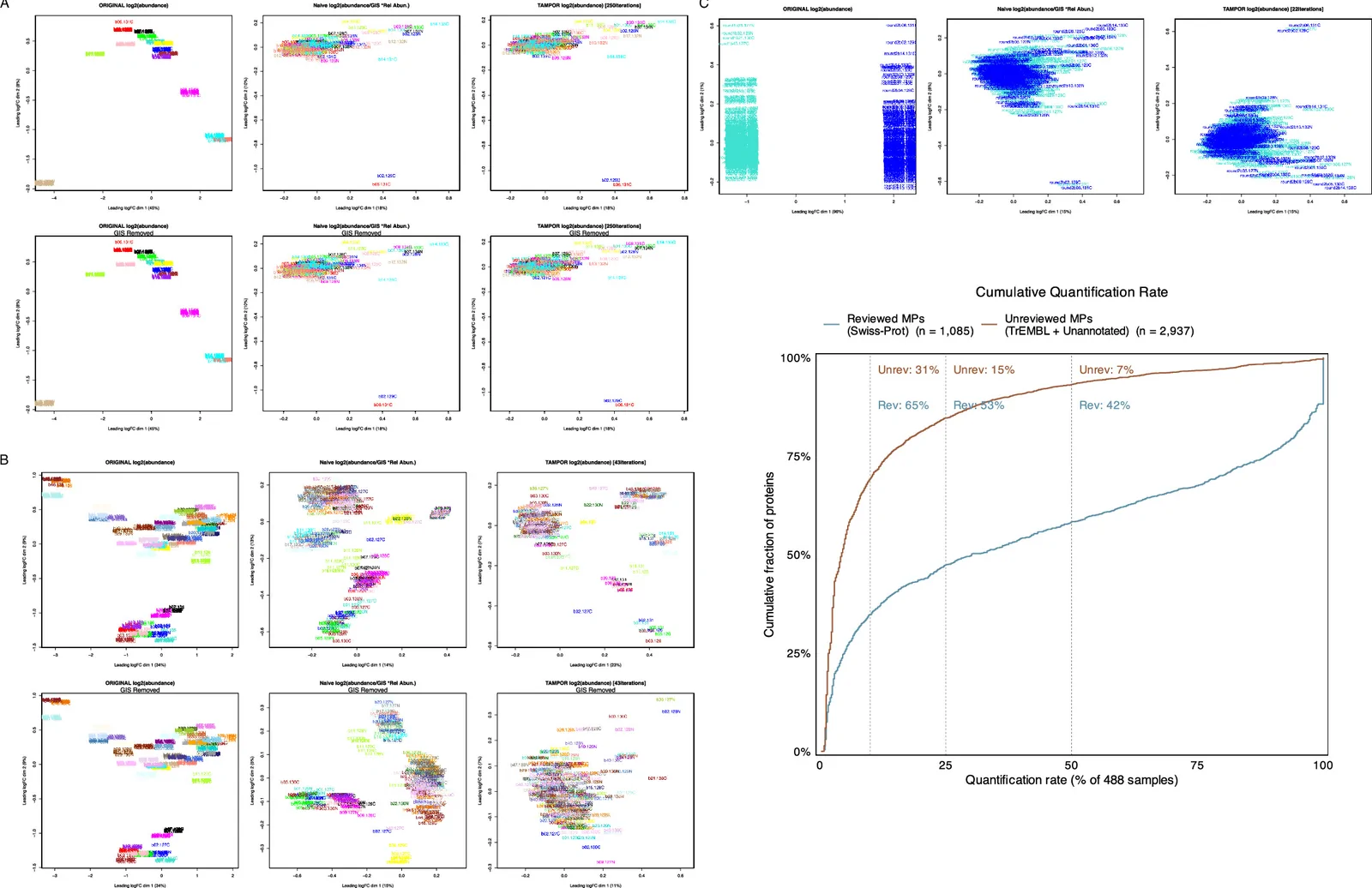
Batch correction of ROSMAP TMT-MS data across two rounds. Multidimensional scaling (MDS) illustrates correction steps for 210 samples (**a**, ROSMAP TMT-MS Round 2) and 400 samples (**b**, ROSMAP-TMT Round 1): starting from raw log2 abundance, then normalized to the global internal standard (GIS), followed by Tunable Approach for Median Polish of Ratio (TAMPOR) batch correction. (**c**) Additional correction across rounds (green = Round 1; blue = Round 2 samples). (**d**) Cumulative microprotein quantification rate (*x* axis) versus cumulative fraction of proteins (*y* axis) with quantitative relative abundance prior to TAMPOR. Source data

TMT labeling enables multiplexed peptide quantification. We performed differential abundance analyses on TMT-MS data from 480 ROSMAP DLPFC samples, grouped into three diagnostic categories per prior criteria: non-AD, asym-AD (pathology without symptoms) and sym-AD (pathology with symptoms), and then carried out a differential abundance analysis between non-AD and sym-AD donors. Given the MP detection challenges, we used a stringent model requiring detection in at least half of samples and a relaxed model using all quantified proteins, each reporting high-confidence (FDR < 0.05) and exploratory (0.05 ≤ FDR < 0.2) tiers (Fig. 3a,b, Extended Data Fig. 3 and Supplementary Table 2).

**Extended Data Fig. 4:**
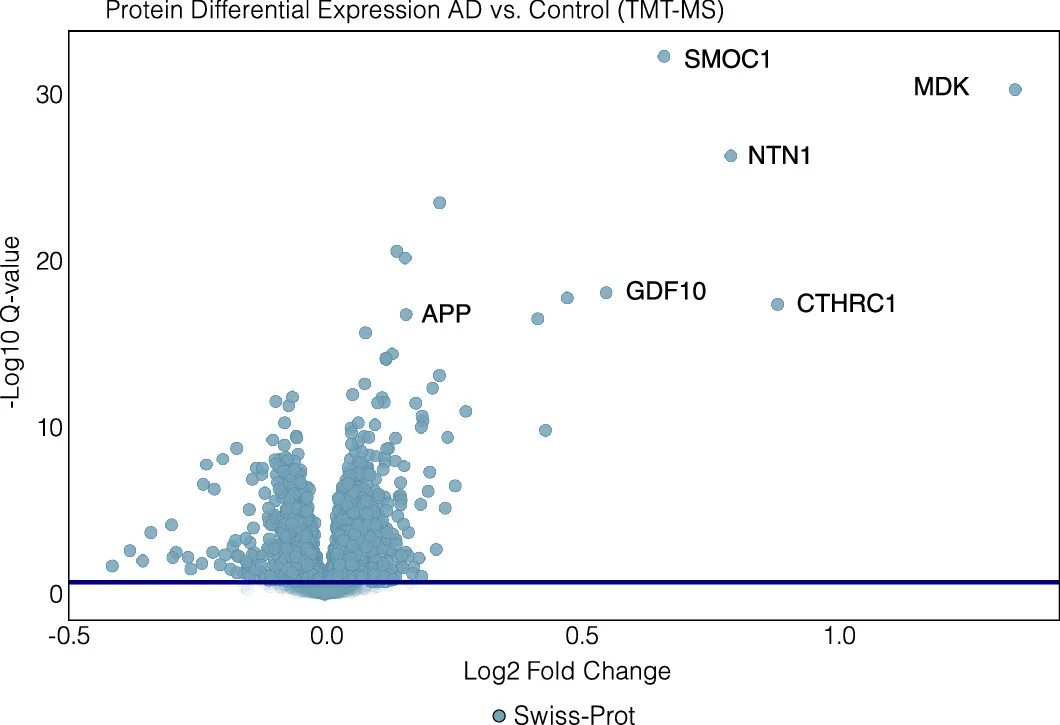
Volcano plot of the UniProt/Swiss-Prot reviewed protein sequences in sym-AD versus non-AD brain samples from ROSMAP TMT-MS. Each point represents a Swiss-Prot-annotated protein quantified across cohorts. The x-axis shows the log_2_ fold change (AD vs. Control), and the y-axis indicates –log10 q from multiple hypothesis correction. Selected proteins with strong differential expression and statistical significance are labeled, including SMOC1, MDK, NTN1, and APP, replicating previous findings from Johnson et al. The horizontal line marks the FDR q-value = 0.2 threshold. Statistics: two-sided Student’s t-test, Benjamini-Hochberg-adjusted FDR (q-value), same stringent model (≥50% detection) as Fig. 3a. Source data

In the stringent model, 99 reviewed MPs (≤ 150 amino acids) were differentially abundant at FDR < 0.05. For example, decreased SST and elevated MDK recapitulated prior ROSMAP findings^23^, validating the framework for interpreting unreviewed MPs (Extended Data Fig. 4). This same framework identified 22 unreviewed MPs at FDR < 0.05, rising to 40 at the exploratory tier (Fig. 3a). The relaxed model recovered 258 unreviewed MPs at FDR < 0.2, spanning all smORF classes (Fig. 3b). Effect sizes reflect directional associations rather than large shifts, as median absolute log_2_ (fold change) among significant stringent-model MPs was 0.0969, consistent with cellular heterogeneity in bulk postmortem tissue^23^.

Proteins that are differentially abundant as revealed through TMT-MS can be further interpreted in their cellular context (Fig. 3c–f). We mapped each MP to its encoding genomic locus and queried published single-nucleus RNA sequencing (snRNA-seq) summary statistics from the same ROSMAP cohort^24^ (Supplementary Table 6). Among Swiss-Prot reviewed proteins shorter than 150 aa, SST, predominantly expressed in inhibitory neurons, was negatively associated with AD pathology across all major cell types including glia (Fig. 3g). MDK showed widespread neuronal and glial expression but was negatively associated with AD only in oligodendrocytes (oligos) despite elevated protein AD brains.

Among unreviewed MPs, we highlight two microglia-enriched examples with altered abundance in AD. BCL3-iORF (Fig. 3e,h) was lower in sym-AD tissue at the exploratory tier of the relaxed differential expression model (FDR < 0.2), whereas the RAB3C N-terminal isoform was significantly higher in the stringent model (FDR < 0.05). BCL3-iORF is most highly expressed in microglia, whereas the RAB3C N-terminal isoform shows microglia-specific expression that is negatively associated with global AD pathology. For both MPs, transient overexpression in HMC3 human microglial cells confirmed stable expression. BCL3-iORF localized to the nucleus, whereas RAB3C showed no nuclear overlap (Fig. 3j).

### A subset of smORFs diverges in expression from the main ORF of the encoding gene

Nearly three-quarters of MS-detected MPs are encoded by loci already annotated to produce a larger canonical protein. Therefore, we asked whether smORF-containing transcripts are regulated independently of those containing the gene’s main canonical ORF (mORF). After quantifying reads mapping specifically to the smORF coding sequence (CDS) versus the mORF CDS, we generated coexpression profiles pairing each gene’s smORF CDS with the same gene’s mORF CDS.

**Fig. 4:**
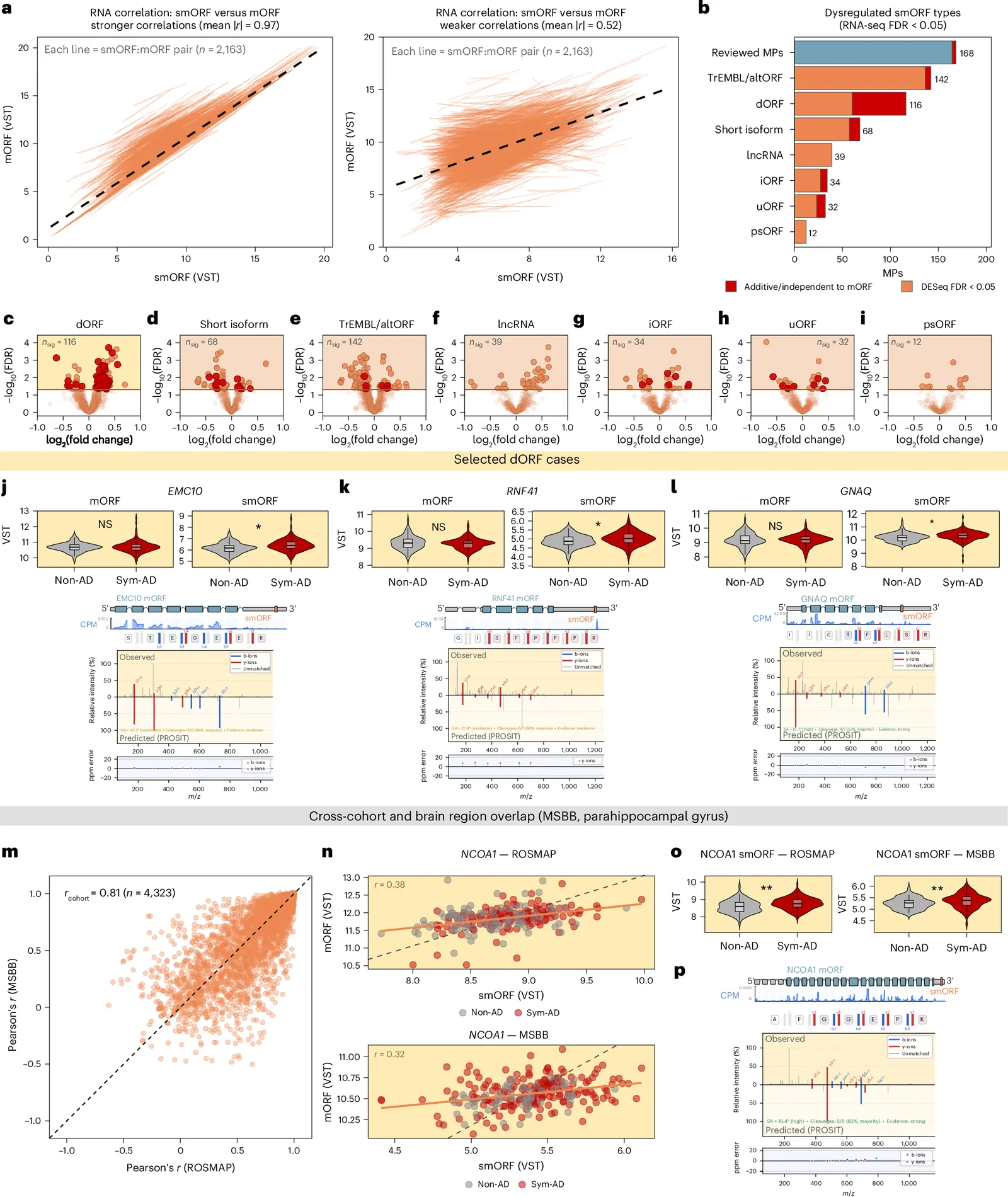
Differential RNA expression of MPs in AD and correlation with their corresponding gene’s mORF. Unless noted, comparisons use ROSMAP (*n* = 90 non-AD/142 sym-AD) and MSBB (*n* = 57 non-AD/179 sym-AD); distinct brain donors; unit of study = individual smORF CDS. The asterisks denote Benjamini–Hochberg FDR: *P < 0.05; **P < 0.01; ***P < 0.001. NS, not significant. **a**, Per-pair Pearson correlation of smORF versus mORF transcript expression across ROSMAP donors (*n* = 4,326 smORF-mORF pairs; each faint orange line = one pair’s linear fit). Pairs are split at the median correlation (|*r*| = 0.868, 50th percentile) into strongly correlated (left; *n* = 2,163, mean |*r*| = 0.97) and weakly correlated (right; *n* = 2,163, mean |*r*| = 0.52) halves. The black dashed line represents a descriptive pooled least-squares fit per subset. **b**, Dysregulated smORF types (RNA-seq FDR < 0.05), according to class, colored according to additive (orange) versus independent (red) mORF expression. Two-sided LRT (additive model), ROSMAP; *n* = 390 smORF-mORF gene pairs; 152 decoupled (LRT *P* < 0.05, uncorrected). **c**–**i**, Volcano plots (log_2_(fold change) versus −log_10_(FDR), sym-AD versus non-AD), according to smORF class: dORF (**c**), short isoform (**d**), TrEMBL/altORF (**e**), lncRNA (**f**), iORF (**g**), uORF (**h**) and psORF (**i**). Two-sided DESeq2 Wald test, Benjamini–Hochberg FDR; ROSMAP. **j**–**l**, Three dORF cases—EMC10 (**j**), RNF41(**k**) and GNAQ (**l**). Variance stabilizing transformed (VST)-normalized smORF expression, non-AD versus sym-AD (ROSMAP). Box plots: center indicates the median; boundaries indicate the 25th–75th percentiles; and whiskers indicate the most extreme points within 1.5× the IQR. Two-sided DESeq2 Wald test, Benjamini–Hochberg FDR; log_2_(fold change): EMC10 = 0.336, FDR = 4.04 × 10^−2^ (*); RNF41 = 0.392, FDR = 1.66 × 10^−2^ (*); GNAQ = 0.188, FDR = 2.63 × 10^−2^ (*). Parent mORFs were not significant for all three (FDR: EMC10 = 0.41, RNF41 = 0.36, GNAQ = 0.62). Independence from the parent mORF was significant through a two-sided additive LRT (EMC10 *P* = 1.16 × 10^−2^; RNF41 *P* = 4.22 × 10^−3^; GNAQ *P* = 6.27 × 10^−3^). **m**, smORF-mORF coexpression, ROSMAP versus MSBB (parahippocampal gyrus); 4,323 shared pairs. Cross-cohort *r* = 0.813. **n**, NCOA1 smORF versus mORF, ROSMAP and MSBB. Pearson’s *r* according to diagnosis: ROSMAP non-AD = 0.276, sym-AD = 0.457; MSBB non-AD = 0.407, sym-AD = 0.277. The global linear-model fit is shown as an orange line. **o**, NCOA1 smORF and mORF expression (VST-normalized), non-AD versus sym-AD, ROSMAP and MSBB. Box plots as in **j**–**l**. Two-sided DESeq2 Wald test, Benjamini–Hochberg FDR: ROSMAP log_2_(fold change) = 0.249, FDR = 2.01 × 10^−3^ (**); MSBB log_2_(fold change) = 0.225, FDR = 7.94 × 10^−3^ (**). **p**, Representative PROSIT mirror plot and Ribo-seq track of the NCOA1-dORF. Source data

We observed a bimodal expression architecture. Half of all smORF-mORF pairs showed near-perfect coexpression (mean |*r*| = 0.97), consistent with shared transcripts, while the other half were more weakly correlated (mean |*r*| = 0.52), indicating partial independent regulation for many smORF-harboring transcripts (Fig. 4a and Supplementary Table 7).

Within this decorrelated fraction, differential smORF expression in AD may reflect isoform-specific regulation. Applying a likelihood ratio test (LRT) across smORF-containing transcripts differentially expressed in sym-AD versus non-AD, we found that 39% of testable pairs showed disease-associated changes independent of the mORF (RNA-seq FDR < 0.05; Fig. 4b–i). dORFs were disproportionately represented (48%, the highest rate among smORF classes) and enriched for moderate-to-strong MS/MS spectral grades, supporting protein-level relevance. Three dORF loci—EMC10, RNF41 and GNAQ—illustrate this, where the mORF remained unchanged in sym-AD while the dORF showed significant fold change, each supported by moderate-to-strong PROSIT grades (Fig. 4j–l).

We next tested whether this decorrelation extended to another RNA extraction, sequencing workflow and brain region by re-analyzing parahippocampal gyrus expression data from the Mount Sinai Brain Bank (MSBB)^25^. The ROSMAP bimodal architecture of smORF-mORF RNA expression was concordant in MSBB (*r* = 0.81; Fig. 4m). For example, a dORF encoded within *NCOA1* is supported by high-confidence spectral evidence, partially decoupled from the mORF in both cohorts (ROSMAP pooled *r* = 0.38; MSBB pooled *r* = 0.32) and significantly upregulated in sym-AD while the mORF remained stable (Fig. 4n–p).

### Posttranscriptional splicing motif enrichment for genes encoding MPs

Expression divergence for genes that express both smORF-containing and mORF-containing isoforms raises a mechanistic question. Do these variants arise from distinct transcription start sites (TSS) or a shared primary transcript via alternative splicing? Aberrant splicing is a hallmark of neurodegeneration and may drive selective isoform dysregulation in AD^26,27^. Even if an MP lacks a direct biological function, its detection reveals previously unrecognized regulation of its encoding gene.

**Extended Data Fig. 5:**
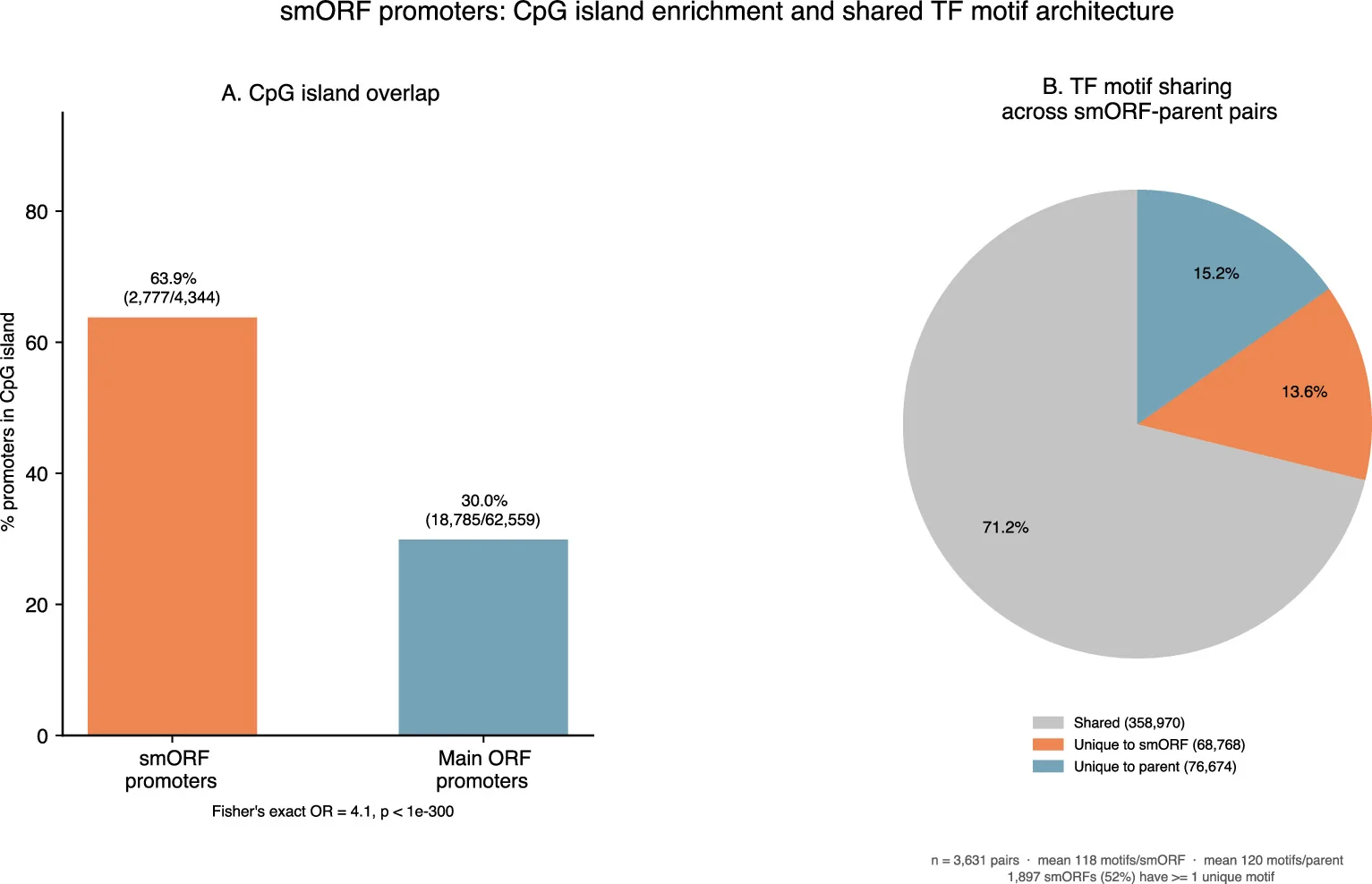
Promoter motif enrichment analyses comparing smORFs genomic coordinates vs. mORF genomic coordinates. Analysis of 3,631 matched genes containing both a MS-detectable smORF-encoded microprotein and its mORF (that is, parent locus) (HOMER v4.11, hg38; TSS window −1000 to +100 bp). Unit of study = individual promoter (**a**) or smORF–parent pair (**b**). (**a**) Proportion of promoters overlapping CpG islands (UCSC cpgIslandExt): smORF promoters 2,777/4,344 (63.9%) vs. mORF promoters 18,785/62,559 (30.0%). Statistics: two-sided Fisher’s exact test, OR = 4.13, P < 1 × 10^−300^. (**b**) Pie chart of transcription-factor motif sharing across the 3,631 matched pairs: 71.2% shared, 13.6% unique to smORF, 15.2% unique to parent (mean 118 motifs per smORF promoter, 120 per parent; 1,897 smORFs [52%] have ≥1 unique motif). Values are descriptive proportions; no statistical test applied. Source data

Alternative transcription initiation does not appear to be the major explanation for genes whose transcripts containing smORFs and mORFs decorrelate, as approximately 75% of smORF promoters shared motif instances with their paired mORF, per HOMER analysis across 3,631 matched gene pairs (Extended Data Fig. 5 and Supplementary Table 8). While a smaller subset with unique promoters may warrant follow-up, independent initiation for transcripts carrying smORFs did not appear to be the predominant explanation for unique transcript expression.

**Fig. 5:**
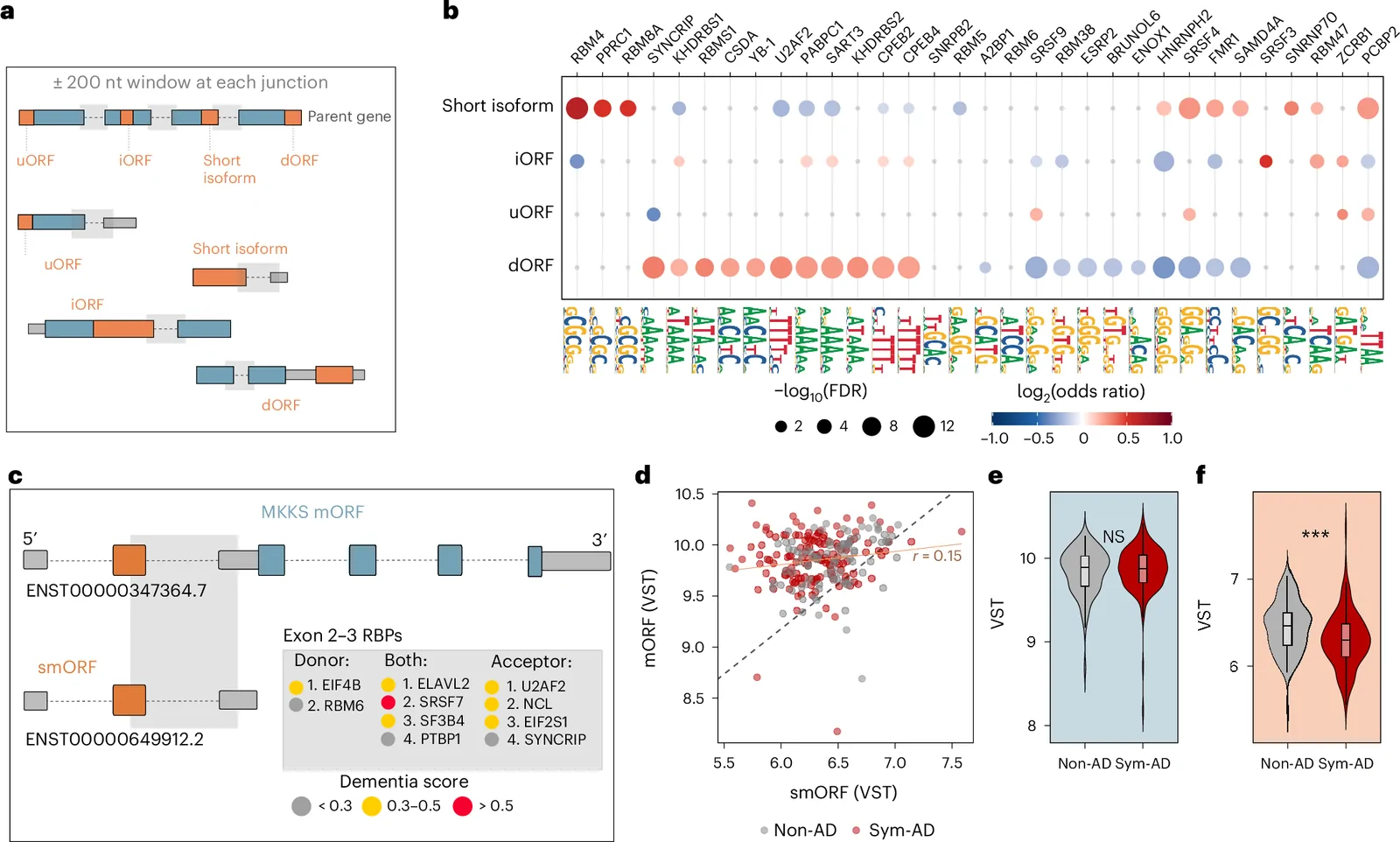
RBP motif enrichment of genes encoding MPs. **a**, Splice-junction RBP motif enrichment approach: a ± 200 nt window scanned at each exon–exon junction, across smORF classes, for RBP recognition motifs. **b**, RBP motif enrichment at smORF–gene splice junctions. Dot plot: dot size = −log_10_(FDR); color (blue–red) = log_2_(odds ratio) per RBP motif and smORF class. Two-sided Fisher’s exact test; smORF-class junctions versus a shared background of junctions from transcripts lacking MP evidence (*n* = 446,044 background junctions); Benjamini–Hochberg FDR within each smORF class. Junction counts: downstream ORF *n* = 16,442; isoform *n* = 7,916; internal *n* = 9,746; upstream ORF *n* = 4,232. **c**, *MKKS* locus architecture: canonical transcript (ENST00000347364.7, teal) and smORF-containing transcript (ENST00000649912.2, orange) diverge at the exon 2–3 junction. Candidate donor and acceptor RBPs are shown with the Open Targets Platform dementia score (< 0.3 gray, 0.3–0.5 yellow, > 0.5 red). **d**, MKKS mORF versus smORF expression (VST-normalized), ROSMAP (*n* = 373 donors: non-AD 90, asym-AD 141, sym-AD 142; non-AD and sym-AD shown). Pearson’s *r* = 0.149. **e**, MKKS mORF RNA expression (VST-normalized), non-AD (*n* = 90) versus sym-AD (*n* = 142), distinct brain donors. Box plots: the center line indicates the median; the bounds indicate the 25th–75th percentiles; and the whiskers indicate the most extreme points within 1.5× the IQR; outliers not shown. Two-sided DESeq2 Wald test (sym-AD versus non-AD): log_2_(fold change) = 0.03, *P* = 0.38, FDR = 0.60. **f**, MKKS smORF expression (VST-normalized), same donors and box plot convention as in **e**. Two-sided DESeq2 Wald test (non-AD versus sym-AD): log_2_(fold change) = −0.20, *P* = 2.01 × 10^−4^, FDR = 8.54 × 10^−3^ (***). Source data

Posttranscriptional splicing emerged as an explanation for smORF-mORF expression decorrelation. RBP motif enrichment at splice junctions in smORF-harboring transcripts differed from transcripts lacking MP evidence by smORF class (Fig. 5a,b and Supplementary Table 9). For example, dORFs showed a distinct 3′ splice site/polyadenylation signature (for example, U2AF2), implicating different posttranscriptional programs depending on smORF location.

At the *MKKS* locus, the smORF-mORF correlation (*r* = 0.15; Fig. 5c,d) falls below the 10th percentile of the decorrelation distribution. Of seven annotated MKKS transcript isoforms (Ensembl), three lack the 570-aa mORF and exclusively encode the 63-aa MP^28^. At the shared second intron splice junction, dementia-associated RBP motifs were identified (Open Targets Platform GraphQL API; Fig. 5c), offering a candidate mechanism that could shift the balance toward full-length over smORF-containing isoforms in AD. The MKKS mORF-carrying transcripts are unchanged in sym-AD (FDR = 0.60), while smORF-containing transcripts are significantly downregulated (log_2_(fold change) = −0.20, FDR = 8.54 × 10^−3^; Fig. 5e,f).

### At the *MKKS* locus, an MP is the predominant translation product, not the mORF, and coexpresses with a mitochondrial gene network

Because *MKKS* was among the most decoupled smORF-mORF pairs in the atlas, we examined its translational output closely. We found that the predominant detectable protein product in human DLPFC is not the 570-aa Swiss-Prot entry (Q9NPJ1), which has been linked to the rare disease McKusick–Kaufman syndrome^4,29^; instead, the predominant detectable protein is a 63-aa smORF-encoded MP cataloged in unreviewed TrEMBL (Q9HB66), which we designated micro-MKKS63.

**Fig. 6:**
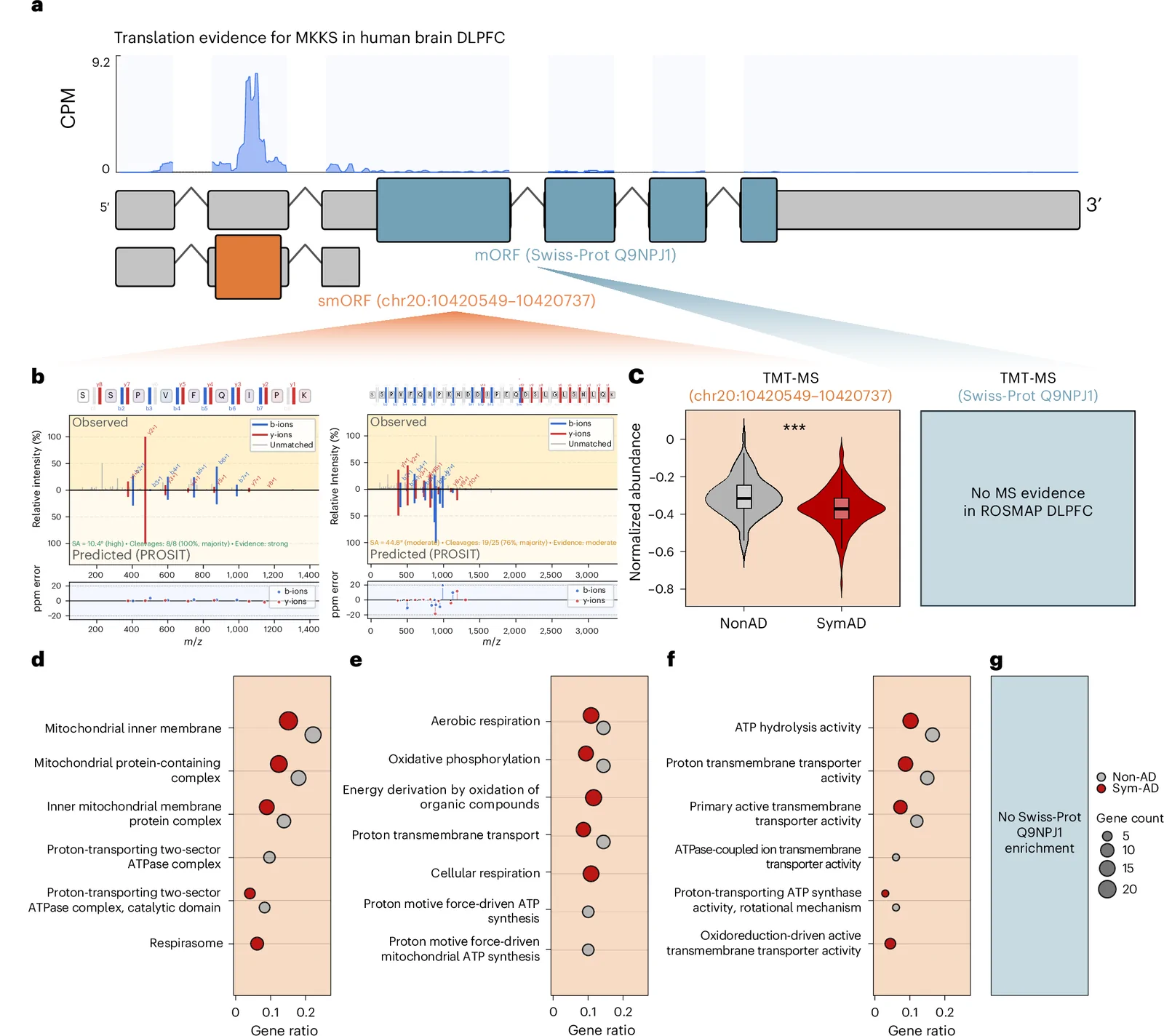
Micro-MKKS63 is the dominant MKKS translation product and coexpresses with a mitochondrial network. **a**, Ribo-seq coverage at the *MKKS* locus, human DLPFC. Strong ribosome occupancy (peak 9.2 CPM) at micro-MKKS63 (chr20:10420549–10420737, orange); sparse occupancy across the canonical Swiss-Prot Q9NPJ1 CDS (teal). Orientation: 5′ to 3′, left to right. **b**, PROSIT mirror plots, two micro-MKKS63-specific tryptic peptides. **c**, TMT-MS normalized abundance of the micro-MKKS63 peptide, non-AD (*n* = 111) versus sym-AD (*n* = 187), distinct brain ROSMAP donors. Violin plot with embedded box plot: the center line represents the median; the bounds represent the 25th–75th percentiles; and the whiskers represent the most extreme points within 1.5× the IQR. Two-sided Student’s *t*-test, Benjamini–Hochberg FDR (stringent model, ≥ 50% detection): *t*_(296)_ = −4.98, log_2_(fold change) = −0.063 (95% CI −0.087 to −0.038), Cohen’s *d* = −0.596, *P* = 1.09 × 10^−6^, FDR = 2.42 × 10^−5^ (***). Right: No MS evidence for canonical Q9NPJ1 in the ROSMAP DLPFC (descriptive; no test performed). **d**–**f**, GO overrepresentation enrichment of the micro-MKKS63 coexpression network, non-AD (gray) versus sym-AD (red): cellular component (**d**), biological process (**e**) and molecular function (**f**). Networks: genes coexpressed with micro-MKKS63 (|*r*| > 0.7), ROSMAP donors (non-AD *n* = 90, sym-AD *n* = 142); unit of study = gene. Gene set sizes: sym-AD *n* = 149, non-AD *n* = 73. Two-sided hypergeometric test (clusterProfiler enrichGO), Benjamini–Hochberg-adjusted *P* < 0.05, FDR < 0.2. Dot size = gene count; *x* axis = gene ratio. **g**, No significant Q9NPJ1 coexpression enrichment, either diagnosis (descriptive; no test performed). Source data

**Extended Data Fig. 6:**
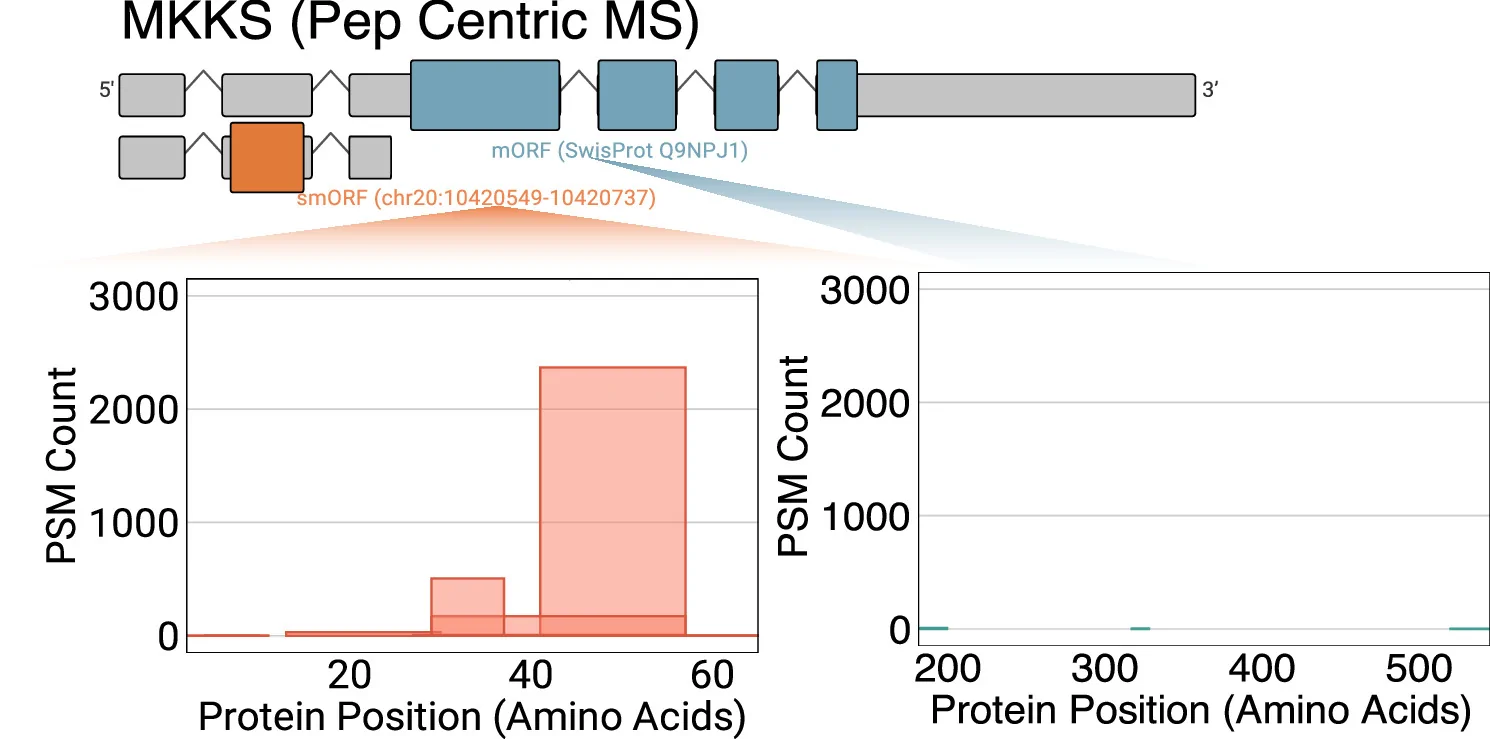
Micro-MKKS63 is the dominant MKKS-derived translation product across public proteomic datasets. MS and ribosome profiling analysis of *MKKS* using PepCentric, a proteogenomic search engine applied to 2.3 billion spectra from 66,700 public MS runs. Canonical MKKS (Q9NPJ1, teal) yields 14 unique PSMs, accounting for <0.1% of all PSMs from the *MKKS* locus when considering micro-MKKS63 (orange). Micro-MKKS63 produces thousands of PSMs, representing the predominant *MKKS*-derived protein across datasets. Source data

Ribosome profiling in human DLPFC revealed localized coverage at the micro-MKKS63 smORF but sparse signal across the main MKKS ORF (Fig. 6a). No peptides from the canonical 570-aa MKKS sequence were detected across the ROSMAP TMT-MS samples, while micro-MKKS63 yielded over 1,000 PSMs, including peptides with strong-grade and moderate-grade coverage (Fig. 6b). A complementary search of the PepCentric meta-database (66,700 public MS runs)^30^ confirmed this imbalance, with only 14 PSMs assignable to canonical MKKS versus thousands for micro-MKKS63 (Extended Data Fig. 6). TMT quantitation showed significantly decreased micro-MKKS63 abundance in sym-AD versus non-AD (Fig. 6c).

To gain functional insight, we built coexpression networks anchored to ROSMAP RNA-seq reads mapping exclusively to the micro-MKKS63 smORF CDS. This network was strongly enriched for mitochondrial biology across all three Gene Ontology (GO) domains (Fig. 6d–f). However, the network anchored to the canonical Q9NPJ1 mORF CDS showed no significant GO enrichment in either condition (Fig. 6g and Supplementary Tables 10–13). As such, micro-MKKS63 expression—not the main MKKS ORF—correlates with processes strongly related to mitochondrial biology.

### Micro-MKKS63 localizes to the mitochondria and CRISPR–Cas9-mediated KO of its smORF lowers microglial oxidative phosphorylation

The mitochondrial coexpression signature of micro-MKKS63 motivated further functional experiments. Given established roles for mitochondria and microglia in AD^31,32^, we tested for endogenous micro-MKKS63 expression in HMC3 human microglial cells. MS on HMC3 protein extracts identified robust micro-MKKS63 detection (PXD071500), with no peptides recovered from the canonical 570-aa MKKS protein, mirroring the pattern seen across ROSMAP brain tissue.

**Fig. 7:**
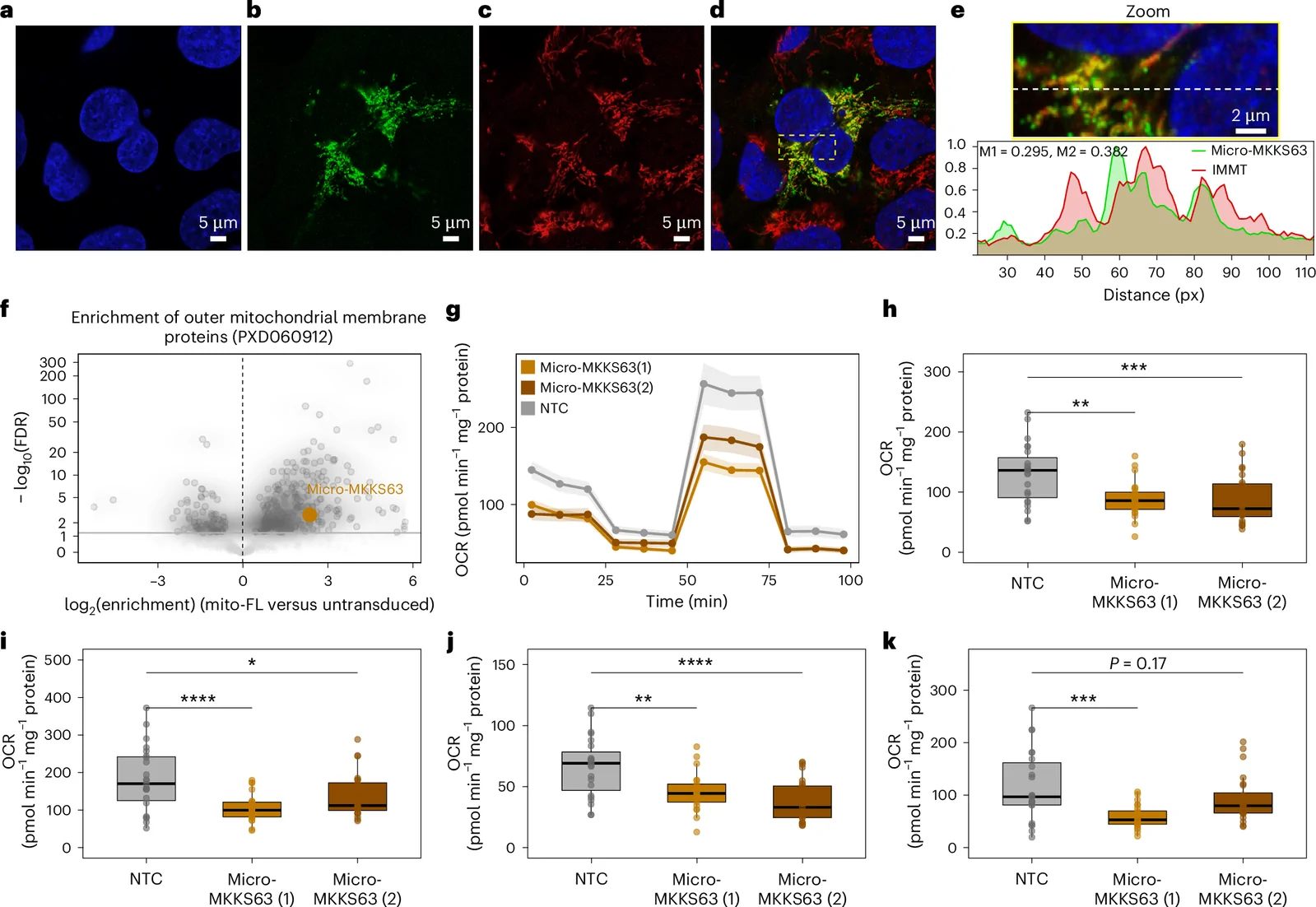
Micro-MKKS63 localizes to mitochondria and Cas9-mediated KO of its encoding smORF disrupts microglial oxidative phosphorylation. **a**–**d**, Immunofluorescence (IF) of HMC3 microglia overexpressing C-terminal FLAG-tagged micro-MKKS63: DAPI (blue) (**a**); IMMT (red) (**b**); micro-MKKS63 (green) (**c**); merge, partial colocalization (**d**). *n* = 3 independent transfections; representative images are shown. **e**, Enlarged boxed region from **d**. Manders coefficients: M1, micro-MKKS63 overlapping IMMT; M2, IMMT overlapping micro-MKKS63. **f**, Re-analysis of fractionated mitochondrial proteomics (PXD060912), comparing cells expressing mitochondrially targeted full-length BirA (mito-FL) versus untransduced controls lacking the biotin ligase (*n* = 3 per group); Benjamini–Hochberg-adjusted two-sided *t*-test. Micro-MKKS63 was enriched in the mitochondrial fraction (log_2_(fold change) = 2.36, 95% CI 1.61–3.12; FDR = 1.41 × 10^−3^). **g**–**k**, Seahorse XF Mito Stress Test, oxygen consumption rate (OCR) in a polyclonal nontargeting control (NTC) cell line versus two independent micro-MKKS63 KO lines, sgRNA1 and sgRNA2. *n* = 23 (NTC), 24 (micro-MKKS63-sgRNA1), 24 (micro-MKKS63-sgRNA2). **g**, OCR time course: the line represents the mean; the shaded bands represent the mean ± s.e.m. **h**–**k**, Basal OCR (**h**), maximal respiration (**i**), ATP-linked OCR (**j**) and reserve capacity (**k**): box plots (center line represents the median; the boundaries represent the 25th–75th percentiles; and the whiskers represent the most extreme points within 1.5× the IQR). Both lines independently reduced OCR relative to NTC. Two-sided one-way analysis of variance (d.f. = 2, 68), Dunnett’s post hoc test versus NTC; mean difference (95% CI) *P*. **h**, *F* = 8.56, *P* = 5 × 10^−4^; sgRNA1 = 41.68 (14.94–68.41), *P* = 0.0015 (**); sgRNA2 = 43.44 (16.71–70.18), *P* = 0.0009 (***). **i**, *F* = 8.96, *P* = 4 × 10^−4^; sgRNA1 = 77.73 (36.17–119.3), *P* = 0.0001 (***); sgRNA2 = 44.59 (3.03–86.14), *P* = 0.0337 (*). **j**, *F* = 14.12, *P* < 0.0001; sgRNA1 = 20.70 (7.40–33.99), *P* = 0.0015 (**); sgRNA2 = 28.89 (16.39–41.39), *P* < 0.0001 (****). ***k***, F = 8.84, *P* = 4 × 10^−4^; sgRNA1 = 57.69 (26.49–88.89), *P* = 0.0002 (***); sgRNA2 = 23.44 (−7.76 to 54.65), NS (*P* = 0.165). *P < 0.05, **P < 0.01, ***P < 0.001, ****P < 0.0001. Source data

We overexpressed a C-terminal FLAG-tagged micro-MKKS63 construct in HMC3 cells and co-immunostained for the mitochondrial marker IMMT, observing signal concentrated at peripheral mitochondrial regions (Fig. 7a–e). To validate this at the endogenous level, we re-analyzed a published BioID proximity-labeling dataset targeting the cytosolic face of the outer mitochondrial membrane (PXD060912)^33^, which confirmed that endogenous micro-MKKS63 ranked among the most highly enriched proteins in that fraction (Fig. 7f and Supplementary Table 14).

To test whether micro-MKKS63 is functionally required for mitochondrial activity, we generated two independent polyclonal CRISPR–Cas9 KO HMC3 lines using single-guide RNAs (sgRNAs) disrupting the micro-MKKS63 smORF while preserving the mORF. Seahorse Mito Stress Test analysis showed significant reductions in basal oxygen consumption rate (OCR), ATP-linked OCR and maximal respiration across KO lines relative to nontargeting controls (Fig. 7g–k), with consistent effects across two independent sgRNA lines, supporting a role for micro-MKKS63 in microglial oxidative phosphorylation.

### Ribosome footprints and MS coverage provide orthogonal evidence for brain-expressed pseudogenes

The MP detection framework used to build this atlas highlights how technical limitations can influence our understanding of genome function. Similar challenges may apply to other genomic elements, such as pseudogenes, which are often classified as nonfunctional because of mutations, including premature stop codons, frameshifts or disrupted splice sites^34^. However, some pseudogenes may be misclassified because they are difficult to detect with conventional approaches^35,36^.

**Extended Data Fig. 7:**
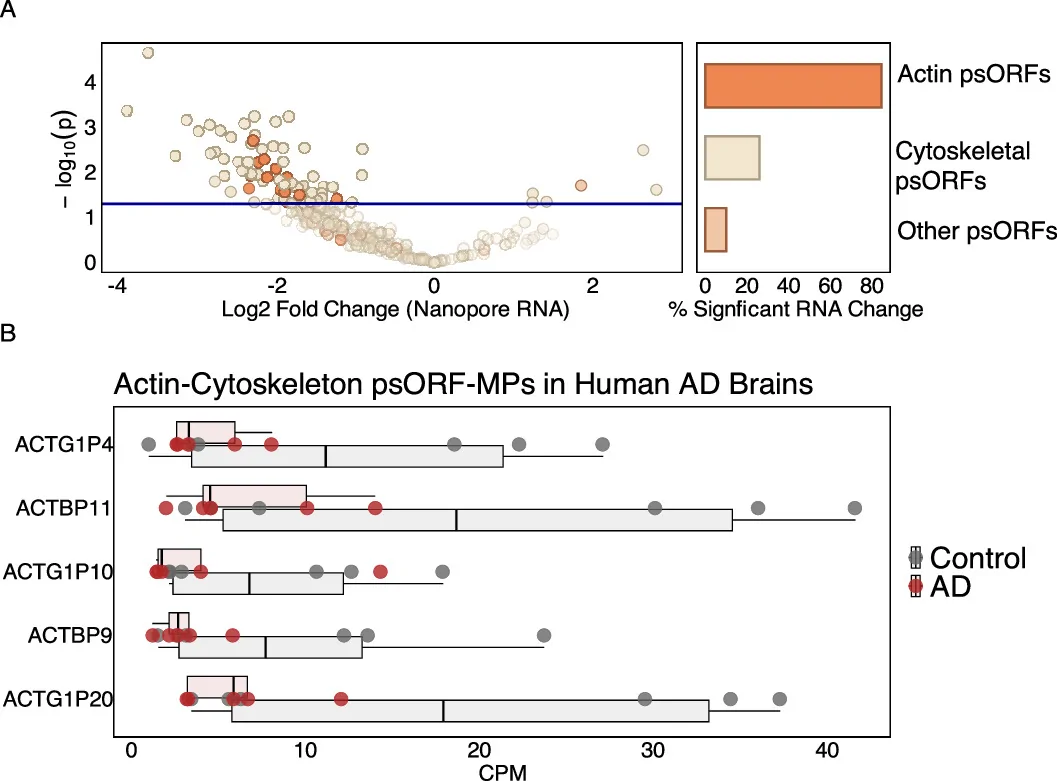
Actin-related pseudogene transcripts are nominally lower in AD brains. Long-read RNA sequencing was used to quantify transcript-level changes between AD (*n* = 6) and control (*n* = 6) brains (Aguzzoli Heberle et al.). Differentially expressed psORFs were then mapped to their ancestral protein-coding paralogs to identify cytoskeletal gene families. (**a**) Volcano plot of differential RNA expression of psORF-coding transcript families, AD vs. control. Statistics: two-sided DESeq2 Wald test (negative binomial GLM, design ~ diagnosis + sex), Benjamini-Hochberg-adjusted FDR; *n* = 6 AD / 6 control biologically independent brains (distinct individuals). Transcripts were called significant at FDR < 0.05 and |log2 fold change|> 1 (colored points). The bar plot (right) summarizes the proportion of significantly altered psORF-coding transcripts by functional class; actin-related and other cytoskeletal pseudogenes show the strongest repression. (**b**) Box plots of representative actin-related psORFs (ACTB/ACTG1-derived: ACTG1P20, ACTBP9, ACTG1P10, ACTBP11, ACTG1P4) with nominally lower RNA expression in AD vs. control. Plotted values are CPM (shown as CPM + 1). Box plots: center line, median; box bounds 25th and 75th percentiles; whiskers extend to the most extreme data points within 1.5× the interquartile range (defining the plotted minima and maxima); points beyond the whiskers are not shown. Statistics: significance is from per-transcript two-sided DESeq2 Wald tests (negative binomial GLM, design ~ diagnosis + sex) computed on rounded integer ESPRESSO counts (not on the CPM values shown); effect size = log2 fold change. All reach nominal (unadjusted) P < 0.05 but do not survive Benjamini-Hochberg correction (FDR ≥ 0.05): ACTG1P20 log2FC = −1.67, P = 0.022; ACTBP9 −1.93, P = 0.025; ACTG1P10 −1.87, P = 0.028; ACTBP11 −1.69, P = 0.033; ACTG1P4 − 1.84, P = 0.046. Source data

To investigate pseudogene RNA expression, we revisited native transcriptomics data from six AD and six non-AD DLPFC samples, the same datasets used to build our MS proteogenomic search space, as short-read RNA-seq cannot reliably assign multi-mapping reads to their originating loci. Actin pseudogene transcripts were broadly downregulated in AD at nominal significance (Extended Data Fig. 7 and Supplementary Tables 15 and 16), motivating us to test whether ribosome footprint coverage, ShortStop functional predictions and unique tryptic peptide detection using MS could together support MP identification from these loci.

**Fig. 8:**
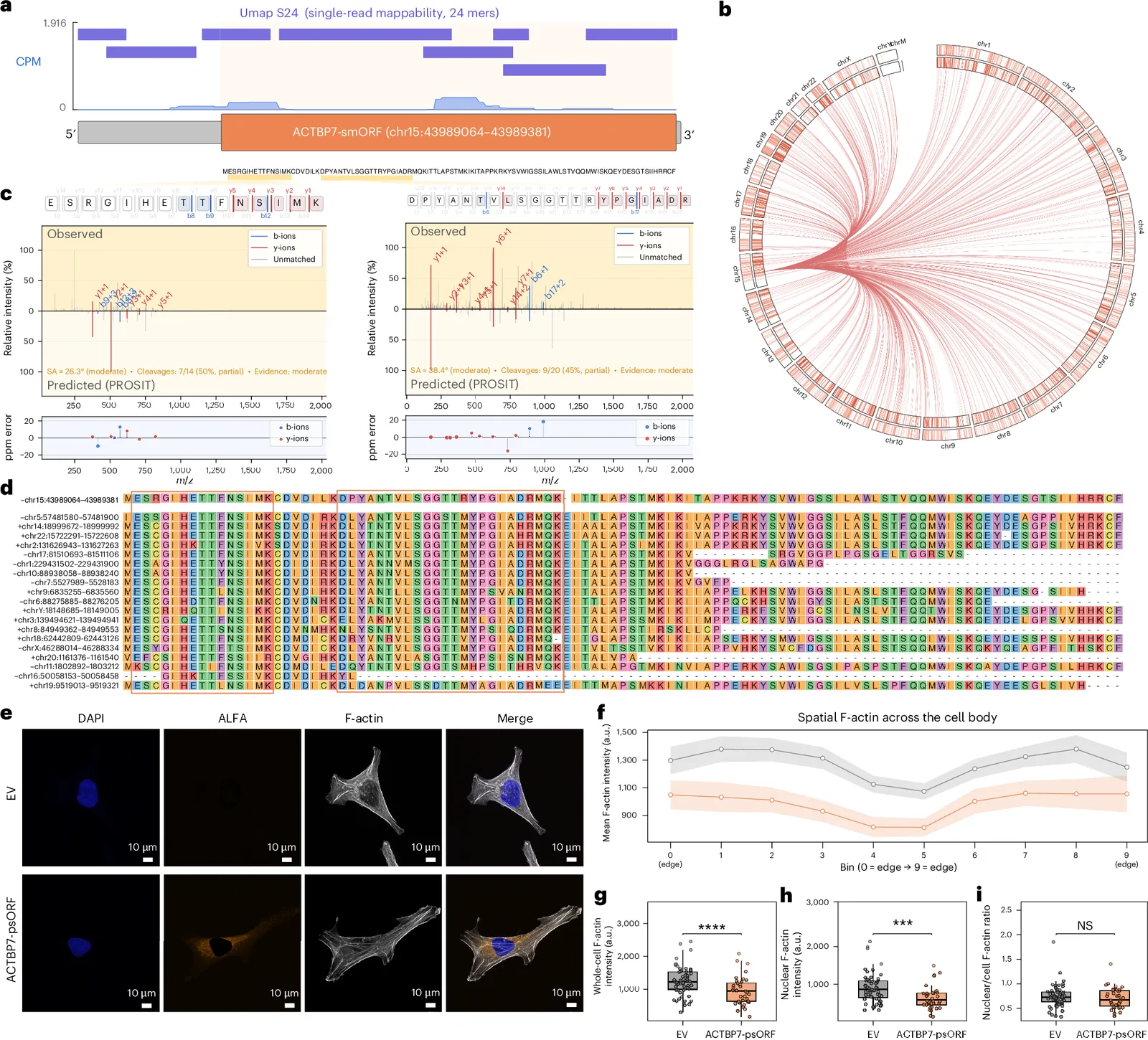
Orthogonal evidence of actin-related pseudogene MP expression. **a**, *ACTBP7* locus view showing unique-mappability and ribosome-occupancy support. Upper track: Single-read mappability (24 mers). Blue histogram: Ribo-seq coverage (CPM). **b**, Genome-wide Circos summary of multi-mapping of the ACTBP7-psORF across chromosomes. The box marks the *ACTBP7* locus and its multi-mapping capacity across the genome. **c**, Representative PROSIT-supported mirror plots for ACTBP7-associated peptides: observed versus predicted fragment-ion intensities, matched b-ions and y-ions, mass error, SA, cleavage coverage and evidence tier. **d**, Multiple-sequence alignment: ACTBP-family and putative ACTB-related sequences. The red boxes mark peptide-supported residues distinguishing ACTBP7 from canonical ACTB and close paralogs. **e**, Functional readout of ACTBP7-psORF overexpression in HMC3 microglia. Representative images; empty vector (EV) versus ACTBP7-psORF: DAPI, ALFA, phalloidin-labeled F-actin and merge. *n* = 3 independent transfections; representative images are shown. **f**–**i**, Effects of ACTBP7-psORF on F-actin distribution in HMC3 microglia (EV = pcDNA3.1-ALFA; ACTBP7-psORF = pcDNA3.1-psORF-ALFA). **f**, Edge-to-center radial F-actin intensity profile; mean ± s.e.m. **g**–**i**, Box plots of whole-cell, nuclear and nuclear-to-cell F-actin intensity/ratio, respectively; unit of study = individual cell. *n* = 3 independent transfections, 13–27 cells per EV transfection and 12 ACTBP7-psORF cells per transfection (total EV *n* = 55, ACTBP7-psORF *n* = 36 cells). Box plots: the center line represents the median; the boundaries represent the 25th–75th percentiles; and the whiskers represent the most extreme points within 1.5× the IQR; outliers are not shown. **g**–**i**, linear mixed-effects model (F-actin metric ~ group + (1 | transfection); restricted maximum likelihood), two-sided Wald test on the plasmid fixed effect; effect size = adjusted mean difference of arbitrary units (a.u.) of fluorescence intensity (ACTBP7-psORF minus EV, 95% CI, raw a.u.). **g**, Whole-cell F-actin: −365.7 a.u. (−529.0 to −202.4, 948.1 vs 1248.3), −29.3%, *P* = 1.14 × 10^−5^ (****). **h**, Nuclear F-actin: −265.3 a.u. (−418.1 to −112.5), −29.5%, *P* = 6.68 × 10^−4^ (***). **i**, Nuclear/cell F-actin: −0.007 (−0.095 to 0.081), −0.9%, *P* = 0.880, NS. *P < 0.05, **P < 0.01, ***P < 0.001, ****P < 0.0001. Source data

**Extended Data Fig. 8:**
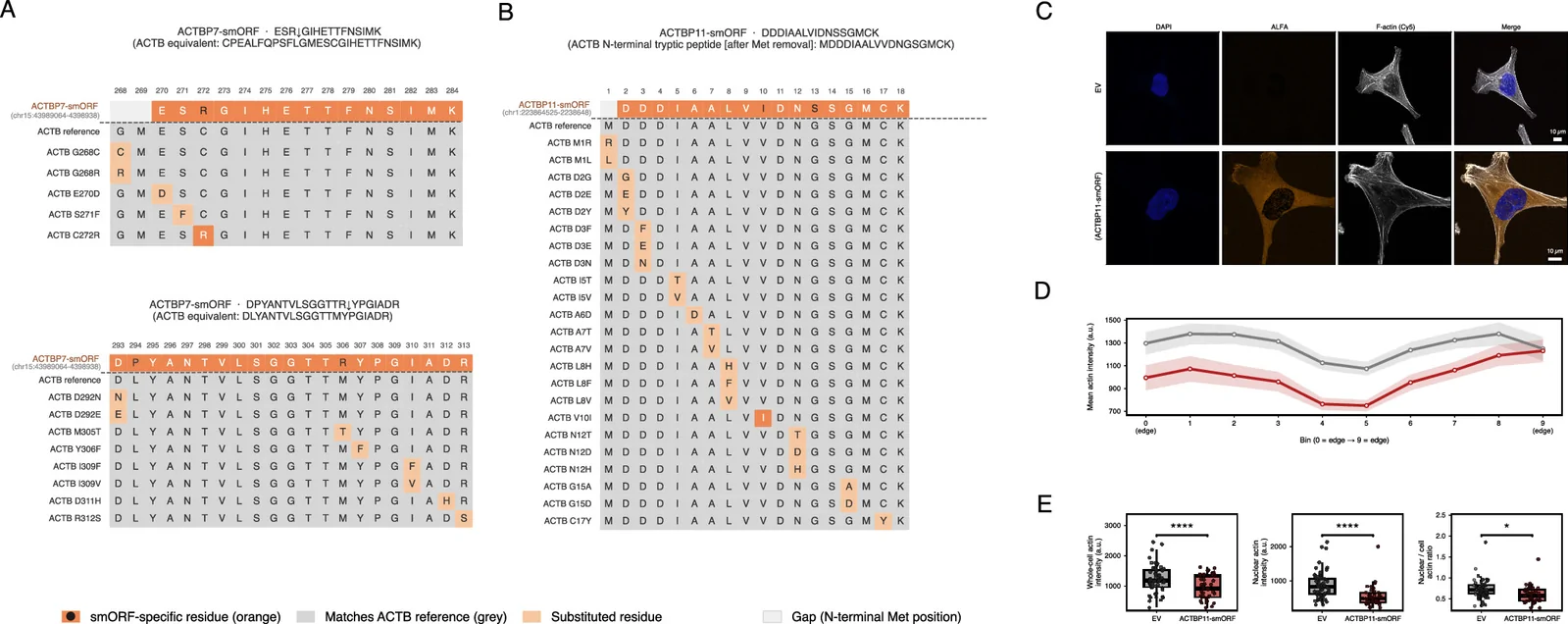
Pseudogene-encoded microproteins are sequence-unique relative to ACTB missense variation. Alignment of two ACTBP7-smORF peptides against the ACTB reference protein (UniProt P60709) and 342 ACTB missense variants (dbSNP). (**a**) Top: peptide ESRGIHETTFNSIMK (ACTB positions 268–284); the aligned Arg corresponds to Cys-272 in ACTB. Bottom: peptide DPYANTVLSGGTTRYPGIADR (ACTB positions 293–313); the aligned Arg corresponds to Met-305. Orange, residues unique to the smORF peptide; grey, residues matching ACTB; peach, missense substitutions matching neither. Alignment of the ACTBP11-smORF peptide DDDIAALVIDNSSGMCK to the ACTB N-terminal window (positions 1–18) across the same ACTB variants. (**b**) No variant matches either smORF peptide, confirming both derive from their smORF loci, not ACTB variation. (**c**) Representative immunofluorescence, HMC3 cells transfected with empty vector (EV, top) or ACTBP11-smORF (bottom): DAPI (nuclei, blue), anti-ALFA (ACTBP11-smORF, orange), phalloidin-Cy5 (F-actin, grey). Transfections performed 3 times. Transfections performed alongside ACTBP7-psORF presented in Fig. 8 and share same EV comparison. (**d–e**) Effect of ACTBP11-smORF vs. EV on F-actin distribution, HMC3 microglia. Unit of study = individual cell. N = 3 independent transfections (biological replicates), 13–27 EV and 12–14 ACTBP11-smORF cells per transfection (total EV *n* = 55, ACTBP11-smORF *n* = 38). (**d**) Radial F-actin intensity profile, edge to center: line, mean; shaded band, 95% CI. (**e**) Box plots of whole-cell, nuclear, and nuclear-to-cell F-actin intensity: center line, median; bounds, 25th–75th percentiles; whiskers, most extreme points within 1.5× IQR; outliers not shown; points, individual cells. Statistics (**e**): linear mixed-effects model, transfection as random intercept for biological replicate structure; two-sided Wald test, ACTBP11-smORF vs. EV; effect size = adjusted mean difference (95% CI, raw a.u.). Whole-cell: −365.7 a.u. (−519.1 to −212.3, 955.1 vs. 1248.3), −29.3%, P = 2.98 × 10^−6^ (***). Nuclear: −345.4 a.u. (−496.0 to −194.7), −38.4%, P = 6.99 × 10^−6^ (***). Nuclear-to-cell ratio: −0.097 (−0.183 to −0.010), −13.1%, P = 0.028 (*). Asterisks denote * P < 0.05; ** P < 0.01; *** P < 0.001. Source data

For *ACTBP7*, ribosome profiling across 43 non-AD DLPFC samples^5^ revealed unique footprint coverage at the psORF (Fig. 8a), although without typical periodic ribosome occupancy. Periodicity cannot be assessed at *ACTBP7* because the window of unique sequence is too short (Fig. 8b). We also mapped two unique tryptic peptides exclusive to ACTBP7 and distinguishable from its ancestral ACTB sequence; both received moderate-grade PROSIT spectra (Fig. 8c,d and Extended Data Fig. 8).

As canonical ACTB is fundamental to cytoskeletal biology, we tested whether ACTBP7-psORF overexpression affects cytoskeletal organization. Its endogenous function is hard to resolve directly because pseudogene loci often are not amenable to CRISPR perturbation and may act only under disease or stress conditions^37^. Therefore, we overexpressed ACTBP7-psORF in HMC3 microglial cells and assessed F-actin dynamics; after 24 h, IF showed a significant reduction in whole-cell F-actin intensity relative to control (Fig. 8e–i), which is consistent with its ancestral actin-related function. We additionally showed a similar effect for an MP encoded by ACTBP11 (Extended Data Fig. 8). This illustrates how orthogonal evidence can motivate functional studies at loci otherwise difficult to interrogate.

## Discussion

Ribosome profiling has established that thousands of smORFs are actively translated. However, extending these catalogs to direct protein-level evidence in aged human tissue and neurodegeneration has remained largely unaddressed. The atlas presented in this article integrates ribosome profiling, MS and a four-tier spectral grading with genomic annotation in the aged human DLPFC. This atlas provides an evidence-graded resource that can directly inform hypothesis-driven experiments on brain MPs in aging and AD.

Our proteogenomic framework offers a scalable model for future MP discovery. Unlike prior studies that often pair transcriptomic and proteomic data from unrelated tissues and donors, our analyses are strictly matched to the same brain region. Searching for MS evidence across more than 600 DLPFC samples using a reference database built from 12 native DLPFC long-read transcriptomes should reduce the risk of unexpressed transcripts inflating the FDR penalty. Verifying RNA expression of MP-coding smORFs in the same donor samples further restricted the search space. This pipeline identified over 3,000 MPs not reviewed in UniProt/Swiss-Prot (Fig. 1), over 1,000 of which strongly match PROSIT-predicted spectra (Fig. 2).

Future workflows can leverage the PROSIT-based spectral grading framework established in this study. False positives remain a major challenge in large-scale proteomics, yet manually inspecting individual PSMs is impractical at scale. Our pipeline instead grades thousands of PSMs empirically, across nearly 2,000 raw files from ROSMAP TMT-MS data. This is especially valuable for MPs, which often lack multiple tryptic peptides. Conventional guidelines require two unique peptides per protein, but MPs rarely meet this threshold given their short length (Fig. 2). By identifying over 800 single-peptide strong-grade unreviewed MPs, our framework improves detection confidence without sacrificing sensitivity. Ribosome footprint quantitation adds orthogonal evidence of translation. This matters most at challenging loci, where read multi-mapping and peptide non-uniqueness complicate detection, as shown for pseudogenes through convergent long-read transcriptomic, ribosome profiling and MS evidence.

Most MPs in this atlas are encoded by loci with existing protein-coding annotations, which is consistent with prior observations across organisms^8,20,38,39^, yet these additional products can reveal regulatory complexity missed by gene-centric analyses. The *NYAP1* locus, encoding an adapter protein in PI3K/Akt signaling and neuron projection morphogenesis, also produces a distinct uoORF-encoded MP with strong spectral evidence (Fig. 2) that carries a predicted TDP-43 (*TARDBP*) splicing motif. Given TDP-43’s central role in frontotemporal dementia and amyotrophic lateral sclerosis (ALS)^26,40^, age-related changes in its activity may alter isoform usage at neurodegeneration-relevant loci in ways standard analyses miss. Similarly, the BCL3 iORF is downregulated in sym-AD at the exploratory tier while canonical BCL3 is unchanged (Fig. 3), showing MPs can be independently regulated and serve as biomarkers distinct from their parent genes, consistent with iORFs representing an understudied source of bioactive peptides^41^.

A reproducible feature of this atlas is that a gene’s smORF-containing transcripts can be expressed independently of the transcripts carrying its main ORF. This pattern held across brain regions, cohorts, and workflows when analyzing ROSMAP and MSBB transcriptomics data (Fig. 4). Alternative promoters explain independent expression for some uORFs separate from their mORF^42^; however, in our studies, nearly three-quarters of MPs share promoters with their main ORFs (Extended Data Fig. 5), pointing instead to posttranscriptional mechanisms. Accordingly, polyadenylation and 3’ splice site factors were enriched at dORF junctions, and GC-rich splicing regulators at short isoform junctions. This suggests isoform-selective RBP activity shapes which transcripts retain smORFs (Fig. 5)^43–45^.

Two interpretive paradigms apply to MPs with evidence of expression. First, an MP may reflect altered transcription or splicing of its encoding gene, serving as a marker of regulatory disruption rather than a driver of disease processes^46–48^. Second, it may function as an independent bioactive polypeptide with activities distinct from its gene’s main canonical protein product. These possibilities are not mutually exclusive and will require endogenous perturbation approaches to distinguish between them. Regardless of the independent functions of MPs, MP-producing loci reveal an additional layer of genomic output that is obscured by conventional gene-level analyses.

The *MKKS* locus illustrates the second paradigm in which the MP itself has independent biology. In human DLPFC, the canonical 570-aa MKKS protein shows minimal ribosome profiling support (Fig. 6) and is largely undetected in both ROSMAP TMT-MS data and the PepCentric meta-database of 66,700 public MS runs (Extended Data Fig. 6). Instead, micro-MKKS63 is the locus’s dominant proteomic product, is downregulated in symptomatic AD, localizes to mitochondria and is required for microglial oxidative phosphorylation (Fig. 7). Although the main MKKS protein product is clinically associated with McKusick–Kaufman syndrome^29,49^, a developmental ciliopathy, its mechanisms may not reflect those of the aged brain. This highlights a broader annotation challenge, whereby the most biologically relevant protein at a locus can vary according to tissue and differ from the canonical annotated product. How often this occurs, and whether some disease-associated loci are shaped by co-encoded MP biology, remains unknown.

Confidence in MP identity scales with the convergence of independent evidence rather than any single criterion. Ribosome profiling establishes translational engagement, MS provides direct peptide-level detection and spectral grading separates reliable identifications from unreliable ones. No single readout is sufficient on its own. Peptide-count thresholds alone would have excluded most bona fide MPs (Fig. 2). Gene-level transcript counts alone would have missed the isoform-specific regulation uncovered for BCL3, RAB3C and MKKS (Figs. 3–5). Pseudogene-derived MPs may also be overlooked (Fig. 8). Integrating these methods enables confident calls where any single one would fail; we expect this convergence-based framework to generalize to MP discovery beyond the brain and beyond AD.

This atlas provides the foundation to test the role of MP-encoding genes as an additional layer of biological regulation relevant to neurodegeneration. The expression profiles, spectral grading outputs and processing pipelines described in this study are publicly available as an evidence-graded reference and template for future MP discovery.

### Technical considerations and limitations

The following technical limitations should be considered for the MP detection workflow used here. First, the absence of MS detection does not imply absence of expression. For instance, over one-third of reviewed MPs lacked MS support despite evidence of RNA expression and translation. Moreover, our MS search database that was built from 12 native transcriptomes may have missed some brain-expressed MPs from unassembled transcript isoforms. Future work should resolve which transcript isoform each detectable MP originates from and examine whether the sequences reported arise from alternative transcripts and/or ribosome slippage, which may present isoforms that our models cannot capture. In addition, our smORF-mORF analyses relied on short-read RNA-seq, which cannot fully resolve isoform-level expression at complex loci; scaling native transcriptomes in future work can help address this. MP nomenclature also remains unstandardized, reflecting each discovery’s historical context rather than any intrinsic difference between proteins. For example, somatostatin meets the size criterion but predates the label by decades, while ELABELA’s inclusion reflects its host transcript’s initial noncoding annotation^50^. Positional labels such as dORF or uORF assume that a canonical ORF exists at the locus, which is not always the case, as at SLC35A4 (ref. ^51^). We retain the MP designation per convention, although terminology may change as standards mature.

### Broader implications

The brain proteome is more complex than current gene models reflect. Analyses anchored to canonical annotations risk overlooking MPs by collapsing transcriptomic reads from the mORF and smORF into a single gene body, as illustrated by *MKKS*. Micro-MKKS63 raises a broader question: if an MP, rather than the canonical protein, is a locus’s main translational output in a given tissue, then functional studies based on canonical annotation may target the less abundant, and potentially less disease-relevant, protein. This framework, integrating ribosome profiling, MS and spectral grading, is not specific to the DLPFC or AD and could support MP discovery elsewhere.

## Methods

### Human postmortem brain cohorts

Human postmortem brain tissue and associated data were collected under institutional review board-approved protocols at the institution of origin. Four autopsy collections were included: the MSBB (AMP-AD Knowledge Portal, Synapse ID: syn3159438), the ROSMAP (AMP-AD Knowledge Portal, Synapse ID: syn3219045), the Sanders-Brown Center on Aging at the University of Kentucky (long-read sequencing data deposited under Synapse ID: syn52047893) and the UC San Diego (UCSD) Shiley-Marcos Alzheimer’s Disease Research Center (DIA-MS data deposited at the PRIDE Archive under accession no. PXD071500). Long-read transcriptomic data were generated from DLPFC (Brodmann area 9/46) samples from the Sanders-Brown cohort using Oxford Nanopore sequencing (*n* = 12; six control, six AD; 50% female; mean age 84.4 ± 6.2 years). These data were used to characterize transcript structures and smORF-containing isoforms. The primary TMT-MS proteomics cohort consisted of ROSMAP DLPFC samples. A total of 610 samples were processed through the proteogenomic pipeline and 608 survived quality thresholds (round 1, *n* = 400; round 2, *n* = 208; 69.1% female; mean age 87.8 ± 4.4 years). Based on neuropathological diagnosis and principal component analysis-based outlier removal, 480 samples were retained for differential peptide analysis (111 non-AD, 182 asym-AD, 187 sym-AD; 69.8% female; mean age 87.9 ± 4.3 years). These samples were used to generate residualized peptide abundance values for statistical analysis. A matched ROSMAP bulk RNA-seq cohort (*n* = 373; 90 non-AD, 141 asym-AD, 142 sym-AD; 66.2% female; mean age 88.0 ± 4.4 years) was used for the transcriptomic analyses corresponding to the TMT-MS cohort. An independent replication RNA-seq cohort was obtained from MSBB parahippocampal gyrus tissue (Brodmann area 36; *n* = 258; 57 non-AD, 22 asym-AD, 179 sym-AD; 64.3% female; mean age 83.8 ± 8.0 years). Fresh-frozen frontal cortex tissue from the UCSD cohort was used for DIA proteomics (*n* = 4; mean age 82.5 years; two with AD, one with amnestic mild cognitive impairment, one cognitively normal). In addition, publicly available ribosome profiling data from Duffy et al.^5^ were re-analyzed to provide an independent translation dataset (*n* = 43 adult brain samples; 41.9% female; mean age 39.6 ± 14.7 years). Neuropathological evaluation used Consortium to Establish a Registry for Alzheimer’s Disease (CERAD) criteria to assess neuritic plaque burden and Braak staging to evaluate neurofibrillary tangle progression. Additional neuropathological diagnoses were assigned according to established criteria and guidelines^52–55^. ROSMAP and MSBB cases were classified using a cross-cohort harmonization framework^23^: non-AD, CERAD 0–1 and Braak 0–3 without dementia at last evaluation (with CERAD required to equal 0 when Braak = 3); asym-AD, CERAD 1–3 and Braak 3–6 without dementia; and symptomatic AD, CERAD 2–3 and Braak 3–6 with dementia. Dementia was defined as a Mini-Mental State Examination < 24 or Clinical Dementia Rating ≥ 1. Per-case demographic and clinical variables are provided in Supplementary Table 17.

### Long-read transcriptome assembly and putative ORF database construction

To support proteomic discovery of MPs, we built a custom putative ORF database by appending unannotated protein sequences absent from the reference human proteome (UniProtKB UP000005640). These sequences were identified using Oxford Nanopore long-read RNA-seq data from dataset syn52047893, generated by the Sanders-Brown Center on Aging at the University of Kentucky^16^. The dataset includes RNA from 12 postmortem brain samples (Brodmann area 9/46). Raw reads were aligned to GRCh38 (with External RNA Controls Consortium spike-ins) using minimap2 (v.2.26-r1175, -t 64 -ax splice -ub -k14 -w 4 --secondary=no), compressed with samtools (v.1.18) and sorted with sambamba (v.1.0.1). ESPRESSO (v.1.0) assembled transcript models and quantified isoforms via its S, C and Q steps against Gencode v.43, using --sort_buffer_size 450 G, --read_num_cutoff 2 and --read_ratio_cutoff 0 for the S and Q steps. The resulting Gene Transfer Format (GTF) and isoform count table (via expectation maximization) were sorted with GFF3sort for frame-preserving translation. We retained isoforms exceeding 0.5 CPM, three-frame translated them using our GTFtoFASTA tool^8^ and appended the resulting peptides to the canonical proteome (UniProtKB UP000005640, 105,486 Swiss-Prot/TrEMBL entries). This yielded 2,174,307 putative ORFs for downstream MS searches.

### TMT-based proteomics and protein identification analysis

ROSMAP TMT-MS data included TMT 10-plex and TMTpro 16-plex labeling, high-pH fractionation and liquid chromatography (LC)–MS/MS on Orbitrap-class instruments. Raw spectral data were analyzed using FragPipe (v.22.0), Philosopher (v.5.0) and IonQuant (v.1.8.5), with searches run in TMT mode using MS^2^ or MS^3^ quantification depending on the batch acquisition method. PSMs were identified via a two-pass search and rescoring workflow. The first pass searched the full proteogenomic database with a precursor and fragment mass tolerance of ±20 ppm, trypsin digestion allowing two missed cleavages and a minimum peptide length of seven amino acids. Carbamidomethylation of cysteine was fixed, while methionine oxidation and protein N-terminal acetylation were variable modifications. TMT reporter ion quantification used manufacturer-provided lot correction factors. Identifications were rescored with Percolator via FragPipe’s MSBooster module and filtered at FDR <0.01 at both PSM and protein levels using Philosopher’s decoy-based approach. The second pass re-searched proteins identified at FDR < 0.01 using the same parameters to improve sensitivity while limiting false positives. Only identifications supported in both passes at 1% FDR were retained.

### smORF classification according to genomic context and isoform homology

We classified smORF-encoded MPs using Ensembl transcript biotype annotation and BLASTP protein homology. Strand-specific overlaps between predicted smORFs and Ensembl models were identified using bedtools (v.2.30.0), with a custom parser assigning annotations according to feature type and biotype. Full smORF definitions are listed in Supplementary Table 18. Isoform-derived smORFs resembling canonical proteins were identified using BLASTP alignment against UniProtKB UP000005640 (E-value 1 × 10^−4^), with bit scores of 40 or greater classified as protein isoforms.

### Ribosome profiling re-analysis using Rp3 to assess translation of MS-identified MPs

To assess translational evidence for MS-detected MPs, we re-analyzed publicly available Ribo-seq data from 43 adult human DLPFC samples (Duffy et al.^5^; dbGaP accession no. phs002489.v1.p1) using our Rp3 framework (v.1.2.2)^39^, which combines proteogenomics searches with Ribo-seq read re-mapping. While RiboCode identifies translated smORFs via 3-nt periodicity and ribosome occupancy, Rp3’s ribocov mode re-maps reads with featureCounts under less stringent, multi-parameter conditions allowing multi-mapping and ambiguous mapping, recovering pseudogenes and multi-copy smORFs that RiboCode’s uniquely mapped read approach would discard. Ribocov trims reads with fastp (adapter/poly(A) removal), removes rRNA and tRNA contamination via STAR (--outSAMstrandField intronMotif, --outReadsUnmapped Fastx), then aligns unmapped reads with STAR (--outSAMstrandField intronMotif, --outFilterMismatchNmax 2, --outFilterMultimapNmax 9999, --chimScoreSeparation 10, --chimScoreMin 20, --chimSegmentMin 15, --outSAMattributes All, --clip5pNbases 3; library-prep specific), the 9,999 cap retaining reads at pseudogenes and complex regions. featureCounts was run four times (default, -M, -O, and -M and -O combined), with coverage plotted as heatmaps via pheatmap. To score the smORF coding potential, the ESPRESSO-generated GTF was input to TransDecoder to annotate CDS features and predict the longest ORF per transcript; a custom STAR (v.2.7.4a) index was built via --sjdbGTFfile alongside GRCh38. Rp3-trimmed reads were aligned to the transcriptome (as required by RiboCode) with STAR (--quantMode TranscriptomeSAM GeneCounts, --outFilterType bySJout, --alignEndsType EndToEnd). We inferred 3-nt periodicity via RiboCode’s prepare_transcripts, metaplots and RiboCode commands (default parameters, 1% FDR cutoff).

### Machine-learning-based classification of translated smORFs using ShortStop

To identify which smORFs detected as translated by RiboCode encode MPs with features of bioactivity, we used our previously reported ShortStop classifier^12^. ShortStop classifies smORFs based on physicochemical and sequence-derived features. The model was trained on two biologically grounded reference sets, with Swiss-Prot annotated proteins shorter than or equal to 150 aa serving as positive examples and physicochemically resembling in silico MPs serving as negative examples. Physicochemically resembling in silico MPs are synthetic smORFs that match the length, codon bias and amino acid composition of actively translated smORFs with stochastic generative sequence order.

### DIA MS of human DLPFC tissue

Human frontal cortex tissue (four 125-mg samples, UCSD Shiley-Marcos Alzheimer’s Disease Research Center) was lysed in 8 M urea in 100 mM sodium phosphate (pH 7.5) with Halt protease/phosphatase inhibitor cocktail (Thermo Fisher Scientific) using a Precellys Cryolys Evolution homogenizer (Bertin Technologies) in 2-ml CK14 tubes with 1.4-mm zirconium beads (3 × 20 s, 6,800 rpm, 30-s rests), then centrifuged (17,000*g*, 30 min, 4 °C). Supernatants were sonicated (3 × 5 s, 30% amplitude, 15 s on ice between cycles), centrifuged again identically and processed for MS; protein concentration was measured using a bicinchoninic acid assay. Extracts were reduced (dithiothreitol), alkylated (iodoacetamide), digested overnight (trypsin/Lys-C), quenched with formic acid, dried, desalted on C18 columns (Nest Group BioPureSPN Mini, cat. no. HUM S18V), dried again and spiked with PRTC standard (cat. no. 88320, Pierce) for normalization. Peptides were separated on a Vanquish Neo (Thermo Fisher Scientific) with an EASY-Spray PepMap Neo column (75 µm × 150 mm, ES75150PN; 75 µm × 500 mm, ES75500PN for 45–60-min runs; Buffer A, 0.1% formic acid and water; Buffer B, 80% acetonitrile and 0.1% formic acid), with MS/MS on an Orbitrap Astral (Thermo Fisher Scientific). All analysis used DIA-NN (v.2.2.0), with a spectral library from a custom FASTA (DDA-identified, RiboCode-identified and ShortStop-identified sequences appended to the human UniProt reference proteome). Search settings were: tryptic digestion (K/R cleavage, not before proline, one missed cleavage); peptide length 6–45 aa; precursor charge +2 to +4, precursor *m/z* 395–1,005, fragment *m/z* 200–1,800; fixed cysteine carbamidomethylation; variable methionine oxidation/N-terminal acetylation; methionine excision enabled; MS/MS tolerance 10 ppm; MS^1^ tolerance 4 ppm; and protein group level 1 reporting. All mzML files were searched simultaneously (parquet output), then filtered in R at *q* ≤ 0.01 and Global.PG *q* ≤ 0.01, requiring at least one proteotypic peptide.

### Spectral validation of MP PSMs using PROSIT

To validate MP PSMs, we compared observed and predicted fragment spectra according to SA. Fragment-ion intensities for all unique tryptic MP peptides were predicted with the Prosit_2020_intensity_TMT model via the Koina API (koina.wilhelmlab.org; NCE 38, HCD)^19^; observed spectra were retrieved from mzML files across 64 TMT batches using pyteomics. For each peptide, up to five candidate PSMs were collected, filtered according to spectral purity (≥ 0.5) and restricted to the rank-1 hit’s charge state and modified sequence; the representative spectrum was chosen by most matched fragment ions, then evidence tier, then lowest SA. Spectral similarity was the normalized SA = 1 − (2/π) × arccos(cos θ), where cos θ is the cosine similarity between matched predicted and observed intensity vectors at 20 ppm tolerance (SA bounded 0–1). Match coverage was the fraction of PROSIT-predicted fragments (normalized intensity > 1 × 10^−4^) with a matched observed peak within tolerance. Each peptide received an evidence label (strong, moderate, weak, insufficient) combining SA rating (high ≤ 20 degrees, moderate 20–45, poor 45–70, terrible > 70) and coverage category (majority > 50%, partial 25–50%, sparse < 25%), according to Wacholder et al.^20^.

### Normalization, covariate adjustment and differential expression analysis of TMT proteomics data

TMT reporter ion intensities were extracted using IonQuant and processed in R via a custom pipeline to integrate, normalize and model proteomic data across both acquisition rounds. Quantified FragPipe protein group tables (protein, peptide and PSM FDR 0.01) were merged across all TMT batches. Normalization used TMT Analysis via Median Polish on Repeated standards, adapted from Johnson et al.^23^ and Dammer et al.^56^, leveraging repeated global internal standards (GIS) to control batch effects while preserving the biological signal. Intensities were normalized to the pooled GIS channel by subtracting log_2_-transformed GIS intensity per sample, then harmonized across batches via median polish, removing batch-specific offsets. This was processed separately for rounds 1 (TMT 10-plex) and 2 (TMTpro 16-plex), then combined. Principal component analysis on the GIS-normalized matrix identified outliers (*z*-score > 3 along the first two principal components). Bootstrap-based linear modeling fitted age, sex, postmortem interval and diagnosis, taking median coefficients. Expression was adjusted by regressing out age, sex and postmortem interval, retaining diagnosis-associated variance for differential analysis, using the model:

$$\begin{array}{l}{\mathrm{Protein}}_{{ij}}={{\upbeta }}_{0}+{{\upbeta }}_{1}\times {\mathrm{Age}}_{j}+{{\upbeta }}_{2}\times {\mathrm{Sex}}_{j}+{{\upbeta }}_{3}\times {\mathrm{PMI}}_{j}\\\qquad\qquad\;+{{\upbeta }}_{4}\times {\mathrm{Batch}}_{j}+{{\upbeta }}_{5}\times {\mathrm{Round}}_{j}+{{\epsilon }}_{{ij}}\end{array}$$

The residual ${{{\epsilon }}}_{{ij}}$ was used for *t*-tests, and *P* values were corrected using Benjamini–Hochberg correction to control the FDR. Analyses used R with custom scripts. This pipeline reproduced previously reported fold changes for UniProt/Swiss-Prot proteins on the same dataset^23^.

### Bulk RNA-seq profiling and differential expression analysis

Transcriptomic profiling was performed on DLPFC tissue from ROSMAP tissues, matched according to individual ID and tissue block to TMT proteomics samples for multi-omic integration; only samples with high-quality RNA-seq and matching proteomics were retained. One subset used 150-bp paired-end reads aligned via STAR (v.2.7.10a) with a splice-aware index and --alignIntronMax 1000000, --outFilterMultimapNmax 2, --outFilterMismatchNoverReadLmax 0.04, producing coordinate-sorted BAMs; a second subset used pre-aligned BAMs directly. Gene expression was quantified with featureCounts (subread v.2.0.1, -O flag for multi-CDS assignment) against Gencode v.43 and a custom GTF appending smORFs of interest, merged into a unified matrix in R. Differential expression used DESeq2 on 373 matched samples (AD *n* = 142, asym-AD *n* = 141, non-AD *n* = 90), with median-of-ratio normalization, empirical Bayes dispersion shrinkage and negative binomial generalized linear models (GLMs) with Wald tests under a multi-factor design. The following model was used.

$$\mathrm{Expression}\sim \mathrm{RIN}+\mathrm{sequencing}\,\mathrm{Batch}+\mathrm{Sex}+\mathrm{Age}+\mathrm{PMI}+\mathrm{Diagnosis}$$

To test transcriptional coupling between MPs and host genes, we computed Pearson correlations between each smORF and parent gene separately in non-AD and sym-AD (VST reads; minimum three observations per group, single Ensembl identifier required). Correlation shift Δ-*r* = r(sym-AD) − r(non-AD) classified pairs as coupled (|Δ-*r*| ≤ 0.1) or non-coupled (|Δ-*r*| > 0.1), tested using Fisher’s *z*-test. Three nested models (null: smORF ~ mORF; additive: + diagnosis; interaction: × diagnosis) used likelihood ratio tests to assess independent AD effects and slope differences between groups. As a replication cohort, MSBB RNA-seq^25^ was processed identically using pre-aligned BAMs against hg19. Genes expressing a smORF and an mORF were assessed for coexpression by constructing Pearson’s correlation values on VST matrices. Cross-cohort concordance used Pearson correlation between ROSMAP and MSBB pair-wise *r* estimates. Analyses used R with DESeq2, limma, dplyr, ggplot2, psych and patchwork.

### snRNA-seq integration

To assess cell-type-specific associations between MP-coding genes and AD pathology, we mapped MS-detected MPs onto previously reported snRNA-seq differential expression data from ROSMAP DLPFC participants (2.3 million nuclei, 54 cell types)^24^. Four candidate genes (*SST*, *MDK*, *BCL3*, *RAB3C*) were examined in depth: log(fold change) and log(CPM) were extracted per cell type and visualized as a heatmap, with cell types grouped into broad classes (excitatory and inhibitory neurons, astrocytes, oligodendrocytes, microglia), marking genes reaching FDR significance (*P*_adj_ < 0.05) per cell type. Analyses used R with dplyr, ggplot2 and ggh4x.

### Transcriptional regulatory and RBP motif analyses

Promoter regions were defined per gene using a custom annotation combining Gencode v.43 protein-coding entries and MP loci, with windows spanning −1,000 to +100 bp relative to each TSS (1,100 bp per gene). Transcription factor motif enrichment used HOMER (v.4.11) findMotifsGenome.pl against a random genomic background, combining de novo discovery with enrichment testing against the HOMER-curated transcription factor database. Combined known and de novo motif models were scanned per gene with HOMER annotatePeaks.pl, applied separately to MP and canonical TSS windows to record motif counts and positions. For each smORF with an annotated parent gene, transcription factor motifs were compared between smORF and parent TSS windows and classified as unique to the smORF, unique to the parent or shared. RBP motif occurrences at splice-junction windows were identified with FIMO (MEME Suite v.5.5.9) against 98 position weight matrices from the CISBP-RNA human RBP database, using a uniform nucleotide background and *P* < 1 × 10^−4^. Each gene was assigned to one of four positional classes (downstream, upstream, internal or isoform) by its predominant smORF type. Statistical testing was two-stage: a pooled Fisher’s exact test (Benjamini–Hochberg FDR correction) compared all smORF-associated junctions against the no-smORF background, retaining RBPs at FDR < 0.05, followed by separate Fisher’s exact tests (independent Benjamini–Hochberg correction) for each smORF subtype against the same background. Top RBPs for visualization were selected according to cross-subtype variance in log_2_(odds ratio). RBP disease associations were queried via the Open Targets Platform GraphQL API (v.4), resolving each gene symbol to an Ensembl ID and retrieving up to 10,000 diseases per gene. Associations were filtered with 12 dementia-related and neurodegeneration-related keywords (dementia, Alzheimer, ALS, frontotemporal, neurodegeneration, Parkinson, Lewy body, Huntington, prion, tauopath, inclusion body, motor neuron disease), retaining the maximum Open Targets overall association score (0–1, integrating genetics, transcriptomics, animal models, literature mining and drug target evidence) across matched diseases per RBP.

### Differential expression analysis of long-read RNA-seq data

After ESPRESSO isoform assembly (v.1.0), expression values were extracted from the resulting .esp file (long-read RNA-seq, 12 DLPFC samples: six with AD, six controls), which reports unnormalized fractional isoform counts. Counts were rounded to integers and modeled in DESeq2 via negative binomial GLMs (Wald tests) on diagnosis and sex. psORF-encoded MPs were grouped according to parental gene family: actin-cytoskeleton (for example, *ACTB*, *ACTG1*), non-actin-cytoskeleton (for example, *TUBA1A/TUBA1B*, *SEPTIN7*, *BIN2*) and other, comparing fold change, expression trends and differential expression proportions across groups. Visualization used ggplot2 and patchwork.

### Cell culture, transient transfection and immunocytochemistry

HMC3 human microglial cells (cat. no. CRL-3304, ATCC) were transfected with pcDNA3.1 constructs bearing C-terminal FLAG or ALFA tags separated from the CDS by a GGSG linker. Constructs were ordered from GenScript and sequence-verified. Cells were transfected using the Neon Transfection System (Thermo Fisher Scientific): 1 × 10^6^ cells in 130 µl Neon Buffer R with 2 µg plasmid DNA, electroporated at 1,200 V, 20 ms, two pulses, then incubated 24 h before lysis or fixation. For immunocytochemistry, cells were seeded in µ-Slide chambers (25,000–75,000 per well, Ibidi), fixed in 4% paraformaldehyde (10 min), washed (2× Tris, 1× PBS), permeabilized (0.1% Triton X-100, 10 min) and blocked (2% BSA, 60 min). Primary antibodies included mouse anti-FLAG M2 (1:1,000 dilution, cat. no. F1804, Sigma-Aldrich), rabbit anti-Mitofilin (1:1,000 dilution, cat. no. 10179-1-AP, Proteintech), rabbit anti-ALFA polyclonal (1:500 dilution, cat. no. N1581, NanoTag) and FluoTag-X2 anti-ALFA 568 (1:500 dilution, cat. no. N1502, NanoTag), with phalloidin (1:400 dilution, Invitrogen) for F-actin. Cells were stained overnight (4 °C) with anti-FLAG M2 and anti-Mitofilin, or for 60 min with FluoTag-X2 anti-ALFA 568 and phalloidin. Secondary detection used anti-mouse Alexa Fluor 488 (cat. no. 115-545-003, Jackson ImmunoResearch) and anti-rabbit Alexa Fluor 594 (cat. no. 111-585-003, Jackson ImmunoResearch). Nuclei were stained with DAPI and fixed-cell preparations were mounted in Ibidi antifade mounting medium (cat. no. 50011). Imaging used a Nikon A1R confocal (×40–60 oil immersion, 488/561-nm, GaAsP PMT, 1.0 AU pinhole, NIS-Elements v.AR.6.10.02), processed in Fiji. Micro-MKKS63/IMMT colocalization was quantified using Otsu-thresholded Manders coefficients (M^1^, M^2^) and Pearson’s correlation within mitochondrial regions of interest, plus a line intensity profile at maximal overlap, using Python (NumPy, SciPy, scikit-image). F-actin distribution was quantified from ND2 images via an automated Python pipeline (GitHub: brain-microprotein-atlas). Nuclei were segmented from DAPI (Gaussian smoothing, Otsu thresholding, watershed, size/shape/circularity filtering); cell bodies were delineated from F-actin (Cy5) via seeded watershed matched to nuclei and area-ratio-filtered. Intensity was profiled along the minor axis in ten bins (nuclear centroid to cortex), with whole-cell and nuclear means extracted separately and assessed using a linear mixed-effects model.

### Acid-based extraction and LC–MS/MS analysis of HMC3 microglial cells

HMC3 microglial cells were lysed using acid-based extraction optimized for low-molecular-weight recovery (50 mM HCl, 0.1% β-mercaptoethanol, 0.05% Triton X-100). Centrifuged supernatants underwent solid-phase extraction on BondElut C8 cartridges (~100 mg C8 per 10 mg lysate protein, Agilent Technologies), conditioned with methanol and equilibrated with triethylammonium formate (TEAF) (pH 3.0). After loading and a TEAF wash, MP-enriched fractions were eluted in 75% acetonitrile/25% TEAF (pH 3.0) and lyophilized. Eluates were digested overnight at 37 °C in 2 M urea in 100 mM tetraethylammonium bicarbonate (pH 8.5) with sequencing-grade trypsin (Promega Corporation), quenched with 5% formic acid and desalted on C18 StageTips. Peptides were analyzed on an Orbitrap Fusion Lumos Tribrid (Thermo Fisher Scientific) with an Easy-nLC 1000, on a 25 cm × 100 µm in-house column (BEH 1.7 µm C18, Waters). Separation ran at 400 nl min^−1^ over 140 min (Buffer B, 0.1% formic acid/90% acetonitrile: 1–25% over 100 min, 25–40% per 20 min, 40–90% per 10 min, 90% per 10 min; Buffer A, 0.1% formic acid/water; 2.5 kV spray). The Lumos ran in data-dependent acquisition mode: full MS at 120,000 resolution (400–1,500 *m/z*, AGC 4 × 10^5^), with a top-speed 3-s cycle selecting precursors for CID in the ion trap. MS/MS used 1.6 *m/z* isolation, AGC 2 × 10^4^, a minimum intensity of 5,000 and injection times of 50 ms (MS^1^) and 35 ms (MS^2^), with monoisotopic precursor selection and 5-s dynamic exclusion. Raw spectra were searched with the standardized FragPipe pipeline and combined FASTA database used for the ROSMAP TMT-MS analysis.

### Coexpression and GO enrichment analysis

To investigate the transcriptional context of micro-MKKS63 relative to the canonical MKKS transcript, we performed coexpression analysis using RNA-seq data from DLPFC samples in the ROSMAP cohort, with count matrices generated as described for the manuscript’s differential expression analyses. VST-normalized expression values were used to compute Pearson correlations between each target transcript’s CDS (micro-MKKS63 at chr20:10420549–10420737 and canonical MKKS ENSG00000125863.20) and all other expressed genes (over 61,000 tested), separately for sym-AD, non-AD and combined groups. Genes with |*r*| > 0.7 were considered strongly coexpressed; network overlap between sym-AD and non-AD was quantified using Jaccard indices. GO enrichment analysis was performed using clusterProfiler with enrichGO() and the org.Hs.eg.db database, assessing the Biological Process, Cellular Component and Molecular Function domains, with terms considered significant at a Benjamini–Hochberg *P*_adj_< 0.05.

### Re-analysis of BioID proximity-labeling MS data

Raw MS data were processed in FragPipe (v.22.0). MSFragger searched the human proteome (UniProt UP000005640, with contaminants and decoys) using trypsin (two missed cleavages), 7–50 residue peptides, 20-ppm tolerances, fixed cysteine carbamidomethylation and variable methionine oxidation, and N-terminal acetylation. Results were filtered to 1% FDR (PSM/peptide/protein) using Percolator, ProteinProphet, and Philosopher, and quantified using IonQuant (MaxLFQ, normalized). Differential enrichment used the IonQuant MSstats output. For proteins quantified in both mito-FL and untransduced conditions (≥ 2 replicates per group, non-zero variance), Welch’s *t*-tests on log_2_ intensities gave Benjamini–Hochberg-corrected log_2_(fold changes) (FDR < 0.05 significant), shown as a volcano plot highlighting micro-MKKS63. Analyses used R (dplyr, broom, ggplot2 packages).

### CRISPR–Cas9 editing of micro-MKKS63 and mitochondrial stress testing

Lentivirus was produced in HEK 293FT cells (cat. no. R70007, Invitrogen) by transfecting pMD2.G (cat. no. 12259, Addgene), pMDLg/pRRE (cat. no. 12251), pRSV-Rev (cat. no. 12253) and either lentiGuide-Puro (cat. no. 52963, Addgene) or lentiCas9-Blast (cat. no. 52962) with PEI-Max (cat. no. 24765, Polysciences); supernatant was collected at 48 h, 0.45-µm-filtered and stored at −80 °C. Stable Cas9-expressing HMC3 cells were generated using transduction with lentiCas9-Blast (multiplicity of infection = 0.5) and blasticidin selection (10 µg ml^−1^), and used as the parental line for all sgRNA work.

Two sgRNAs targeting micro-MKKS63 were designed with CCTop^57^ (--pam NGG --fwdOverhang CACCG --revOverhang AAAC --maxOT 5): sgMicro-MKKS63(1), 5′-ATTCCTGAGCAAGATAGTCT-3′; and sgMicro-MKKS63(2), 5′-AGTCCCAGACTATCTTGCTC-3′. A nontargeting control from the Brie library^58^ (5′-AAAAAGCTTCCGCCTGATGG-3′) served as the negative control. Complementary sgRNA oligonucleotides (Eton Bioscience) containing BsmBI-compatible or Esp3I-compatible overhangs were annealed, phosphorylated with T4 PNK and cloned using Golden Gate assembly into an in-house lentiviral sgRNA backbone derived from lentiGuide-Puro. The puromycin-resistance cassette was replaced with BFP for nontargeting controls or mCherry for micro-MKKS63-targeting sgRNAs. Assemblies were transformed into Stbl3 *Escherichia coli*, selected on 100 µg ml^−1^ ampicillin; single colonies were expanded and sequence-verified. Cas9-expressing HMC3 cells were transduced (multiplicity of infection = 0.3), and polyclonal marker-positive populations were recovered using fluorescence-activated cell sorting against untransduced HMC3. Editing was confirmed using PCR across the cut site (forward, 5′-GTAATGGTAAGGAGTCAGGGGCT-3′; reverse, 5′-TGTTGCCAAAAAGTCAGTATTGC-3′; ~700 bp) followed by Sanger sequencing and TIDE analysis^59^. Constructs generated in this study are available from the corresponding author upon request. Cas9/sgRNA HMC3 cells were seeded at 40,000 per well in Seahorse XF96 plates and assayed with the XF Mito Stress Test (oligomycin 2 µM, CCCP 3 µM, rotenone and antimycin A 1 µM each) on a Seahorse XF96 Analyzer. The experiment was performed in two polyclonal micro-MKKS63 cell lines using two sgRNAs across three independent biological replicates per day with similar results. OCR was normalized to total protein per well (bicinchoninic acid assay) and expressed as OCR per mg per experiment.

### Statistics and reproducibility

Sample sizes were not statistically predetermined but are consistent with previous publications; our TMT-MS workflow reproduced the direction and magnitude of previously reported effects on the same ROSMAP datasets^23^. Cohort sizes reflect donors with high-quality data (12 long-read RNA-seq, 610 TMT proteomics, 373 matched bulk RNA-seq); in vitro sizes followed standard practice. For TMT proteomics, technical outliers (|*z*-score| > 3 along the first two GIS-normalized principal components) were excluded, yielding 480 individuals; no other data were excluded. Data collection and analysis were not blinded; cohort studies were observational, with diagnoses by established criteria (CERAD, Braak, Mini-Mental State Examination); cell and CRISPR experiments compared genotypes and treatments without randomization. Statistical tests, detailed in the Methods, spanned two-sided *t*-tests, negative binomial GLM Wald tests (DESeq2), Fisher’s exact tests, Fisher *z* and likelihood ratio tests, Wilcoxon rank-sum tests and one-way analysis of variance with post hoc testing. Normality was assumed but not formally tested; parametric analysis figures show box plots, violin plots and individual points. Multiple testing was Benjamini–Hochberg-corrected (FDR/*P*_adj_< 0.05 unless otherwise stated). Analyses used R (v.4.4.1) and, where noted, Python (NumPy v.2.0.2, SciPy v.1.13.1, scikit-image v.0.24.0). Analyses used R with DESeq2 (v.1.44.0), limma (v.3.60.6), dplyr (v.1.1.4), ggplot2 (v.4.0.0), psych (v.2.6.1), patchwork (v.1.3.2), ggh4x (v.0.3.1), broom (v.1.0.10) and clusterProfiler (v.4.12.6), and, where noted, Python (NumPy v.2.0.2, SciPy v.1.13.1, scikit-image v.0.24.0).

### Reporting summary

Further information on research design is available in the Nature Portfolio Reporting Summary linked to this article.

## Supplementary information

Supplementary Information 1. All data and code generated in this study are publicly available across four repositories. 2. Source data for all figures are provided.

Reporting Summary

Peer Review file

Supplementary Tables 1–18.

## Source data

Source Data Fig. 1 Statistical source data.

Source Data Fig. 2 Statistical source data.

Source Data Fig. 3 Statistical source data.

Source Data Fig. 4 Statistical source data.

Source Data Fig. 5 Statistical source data.

Source Data Fig. 6 Statistical source data.

Source Data Fig. 7 Statistical source data.

Source Data Fig. 8 Statistical source data.

Source Data Extended Data Fig. 1 Statistical source data.

Source Data Extended Data Fig. 2 Statistical source data.

Source Data Extended Data Fig. 3 Statistical source data.

Source Data Extended Data Fig. 4 Statistical source data.

Source Data Extended Data Fig. 5 Statistical source data.

Source Data Extended Data Fig. 6 Statistical source data.

Source Data Extended Data Fig. 7 Statistical source data.

Source Data Extended Data Fig. 8 Statistical source data.

## Data Availability

The data used in this study were in part obtained through the AD Knowledge Portal (https://doi.org/10.7303/9618238). Transcriptomics and associative data were provided by the Rush Alzheimer’s Disease Center, Rush University Medical Center; additional phenotypic data can be requested from www.radc.rush.edu. Transcriptomics data were also generated from postmortem brain tissue collected through the Mount Sinai VA Medical Center Brain Bank and provided by E. Schadt from Mount Sinai School of Medicine. The TMT proteomics data were based on samples provided by the Rush Alzheimer’s Disease Center, Rush University Medical Center. The raw and processed data used in this study are available through multiple public repositories. Data from the AMP-AD Knowledge Portal (https://adknowledgeportal.synapse.org), including the raw and processed datasets from ROSMAP (Synapse ID: syn3219045) and MSBB (Synapse ID: syn3159438), and methods developed by the AMP-AD Target Discovery Program (supported by the NIA), are shared without publication embargo and are available for secondary analysis; access requires registration and adherence to data use and attribution guidelines (https://adknowledgeportal.synapse.org/#/DataAccess/Instructions). ROSMAP resources can also be requested directly through the Rush Alzheimer’s Disease Center (www.radc.rush.edu). All raw long-read sequencing data generated from 12 postmortem human brain samples are available through Synapse (ID: syn52047893) under restricted access, requiring a free Synapse account. The Ribo-seq data from Duffy et al.^5^ are available through dbGaP under accession no. phs002489.v1.p1. The UniProtKB human proteome database (Swiss-Prot and TrEMBL reference sequences, UP000005640, downloaded on 20 January 2025) used for the spectral searches is available at https://ftp.uniprot.org/pub/databases/uniprot/previous_releases/. Raw MS data for the HMC3 studies and the UCSD DIA-MS cohort have been deposited at the PRIDE Archive under accession no. PXD071500.

## Code availability

Code is available on GitHub at https://github.com/brendan-miller-salk/brain-microprotein-atlas. The repository includes analysis scripts, custom pipelines, results, BED/GTF/FASTA files and tools for MP annotation and visualization. The code and data can be further visualized and browsed at https://huggingface.co/spaces/brmiller/brain-microprotein-atlas-app.
