# Supplementary material for: A microprotein atlas of the human frontal cortex in Alzheimer’s disease
Source: Nat Aging. 2026 Sep 14;6(9):1979–97. doi: 10.1038/s43587-026-01207-x (PMC13577902; doi:10.1038/s43587-026-01207-x)
Supplement: Supplementary file 1 [file 43587_2026_1207_MOESM1_ESM.pdf]

nature aging

Supplementary information https://doi.org/10.1038/s43587-026-01207-x

A microprotein atlas of the human frontal cortex in Alzheimer's disease

In the format provided by the authors and unedited

1. **All data and code generated in this study are publicly available across four repositories.**
   - Raw and processed mass spectrometry proteomics data are deposited at the PRIDE Archive (EMBL-EBI) under accession PXD071500 (https://www.ebi.ac.uk/pride/archive/projects/PXD071500).
   - Processed datasets and figure source files are available as a Hugging Face dataset (https://huggingface.co/datasets/brmiller/brain-microprotein-atlas).
   - An interactive, browsable dashboard for exploring the atlas is hosted as a Hugging Face Space (https://huggingface.co/spaces/brmiller/brain-microprotein-atlas-app).
   - A permanently archived, citable snapshot of all data is deposited at Zenodo (DOI: 10.5281/zenodo.20045161; https://doi.org/10.5281/zenodo.20045161). It can be cloned locally to enable GitHub repo analysis.

2. **Source data for all figures is made available:**

File name: Source_Data_Figure1.xlsx
Description:

Sheet 1 (C_ProteinLength): Fig. 1c, protein-length density of MS-detected microproteins. **Columns:** sequence: microprotein amino acid sequence (row identifier); review_status: annotation tier ("Reviewed MPs" = Swiss-Prot, manually curated; "Unreviewed MPs" = TrEMBL plus novel transcriptome-reconstructed smORFs); gene_symbol: host/parent gene symbol; smorf_type: fine-grained smORF class (e.g. dORF, uORF, iORF, uoORF, lncRNA, psORF, TrEMBL, N-Iso); protein_length: microprotein length in amino acids (<=150).
Sheet 2 (D_smORF_Location): Fig. 1d, smORF location and class counts. **Columns:** sequence: microprotein amino acid sequence (row identifier); review_status: annotation tier ("Reviewed MPs" = Swiss-Prot, manually curated; "Unreviewed MPs" = TrEMBL plus novel transcriptome-reconstructed smORFs); gene_symbol: host/parent gene symbol; smorf_type: fine-grained smORF class (e.g. dORF, uORF, iORF, uoORF, lncRNA, psORF, TrEMBL, N-Iso); consolidated_type: collapsed display class (Reviewed MPs, Downstream ORF, Short-Isoform, TrEMBL/altORF, Internal ORF, lncRNA, Upstream ORF, psORF).
Sheet 3 (E_SpectralCounts_byType): Fig. 1e, spectral counts by smORF type. **Columns:** sequence: microprotein amino acid sequence (row identifier); review_status: annotation tier ("Reviewed MPs" = Swiss-Prot, manually curated; "Unreviewed MPs" = TrEMBL plus novel transcriptome-reconstructed smORFs); gene_symbol: host/parent gene symbol; smorf_type: fine-grained smORF class (e.g. dORF, uORF, iORF, uoORF, lncRNA, psORF, TrEMBL, N-Iso); consolidated_type: collapsed display class (Reviewed MPs, Downstream ORF, Short-Isoform, TrEMBL/altORF, Internal ORF, lncRNA, Upstream ORF, psORF); total_unique_spectral_counts: number of peptide-spectrum matches (PSMs) from uniquely-mapping peptides; total_razor_spectral_counts: number of PSMs including razor (shared) peptides; log10_psm: log10 of the spectral count.
Sheet 4 (F_SpectralCount_Zscore): Fig. 1f, spectral-count z-score distribution. **Columns:** sequence: microprotein amino acid sequence (row identifier); review_status: annotation tier ("Reviewed MPs" = Swiss-Prot, manually curated; "Unreviewed MPs" = TrEMBL plus novel transcriptome-reconstructed smORFs); gene_symbol: host/parent gene symbol; smorf_type: fine-grained smORF class (e.g.

dORF, uORF, iORF, uoORF, lncRNA, psORF, TrEMBL, N-Iso); db_group: database group ("Reviewed MPs (Swiss-Prot)" or "Unreviewed MPs (TrEMBL + Novel)"); total_unique_spectral_counts: unique-peptide PSM count; zscore: standardized spectral count (z = (x - mean)/SD).

Sheet 5 (G_RNA_vs_Protein): Fig. 1g, RNA vs protein abundance correlation. **Columns:** sequence: microprotein amino acid sequence (row identifier); review_status: annotation tier ("Reviewed MPs" = Swiss-Prot, manually curated; "Unreviewed MPs" = TrEMBL plus novel transcriptome-reconstructed smORFs); gene_symbol: host/parent gene symbol; smorf_type: fine-grained smORF class (e.g. dORF, uORF, iORF, uoORF, lncRNA, psORF, TrEMBL, N-Iso); db_group: Reviewed / Unreviewed database group; spectral_counts: protein abundance (TMT-MS spectral counts); ROSMAP_BulkRNAseq_CPM: bulk RNA-seq abundance in counts-per-million (ROSMAP); log10_spectral_counts: log10 of spectral_counts; log10_CPM_plus1: log10(CPM + 1); spectral_count_bin: unique-peptide bin ("0-1", "1-2", ">2").

Sheet 6 (I_DetectionEvidence): Fig. 1i, microproteins by detection evidence. **Columns:** sequence: microprotein amino acid sequence (row identifier); review_status: annotation tier ("Reviewed MPs" = Swiss-Prot, manually curated; "Unreviewed MPs" = TrEMBL plus novel transcriptome-reconstructed smORFs); gene_symbol: host/parent gene symbol; smorf_type: fine-grained smORF class (e.g. dORF, uORF, iORF, uoORF, lncRNA, psORF, TrEMBL, N-Iso); detection_evidence: "MS-Detected" or "Ribo-Seq Only".

Sheet 7 (J_RiboSeq_Expression): Fig. 1j, ribosome occupancy by detection evidence. **Columns:** sequence: microprotein amino acid sequence (row identifier); review_status: annotation tier ("Reviewed MPs" = Swiss-Prot, manually curated; "Unreviewed MPs" = TrEMBL plus novel transcriptome-reconstructed smORFs); gene_symbol: host/parent gene symbol; smorf_type: fine-grained smORF class (e.g. dORF, uORF, iORF, uoORF, lncRNA, psORF, TrEMBL, N-Iso); evidence_group: Reviewed/Unreviewed x MS-Det./Ribo-Only ("Reviewed MS-Det.", "Reviewed Ribo-Only", "Unreviewed MS-Det.", "Unreviewed Ribo-Only"); RP3_Default: ribosome-profiling expression (RPKM) from 42 NonAD DLPFC brains; log10_RP3_plus1: log10(RPKM + 1).

File name: Source_Data_Figure2.xlsx
Description:

Sheet 1 (B_PeptideGradeMatrix): Fig. 2b, peptide grade matrix (one row per NC peptide). **Columns:** sequence: microprotein amino acid sequence (row identifier); review_status: annotation tier ("Reviewed MPs" = Swiss-Prot, manually curated; "Unreviewed MPs" = TrEMBL plus novel transcriptome-reconstructed smORFs); tryptic_peptide: observed tryptic peptide sequence; SA_rating: spectral-angle tier (High >0.78, Moderate 0.50-0.78, Poor 0.22-0.50, Terrible <0.22); Match_coverage: fragment-ion coverage tier (Sparse <25%, Partial 25-50%, Majority >50%); Confidence: assigned grade (Strong, Moderate, Weak, Insufficient).

Sheet 2 (C_SpectralAngle_byConf): Fig. 2c, per-protein spectral angle by confidence grade. **Columns:** sequence: microprotein amino acid sequence (row identifier); review_status: annotation tier ("Reviewed MPs" = Swiss-Prot, manually curated; "Unreviewed MPs" = TrEMBL plus novel transcriptome-reconstructed smORFs); smorf_type: fine-grained smORF class (e.g. dORF, uORF, iORF, uoORF, lncRNA, psORF, TrEMBL, N-Iso); Confidence: per-protein confidence grade; median_SA: median spectral angle across the protein's peptides.

Sheet 3 (D_Grade_bysmORFType): Fig. 2d, confidence grade by smORF type. **Columns:** sequence: microprotein amino acid sequence (row identifier); review_status: annotation tier ("Reviewed MPs" = Swiss-Prot, manually curated; "Unreviewed MPs" = TrEMBL plus novel transcriptome-reconstructed smORFs); smorf_type: fine-grained smORF class (e.g. dORF, uORF, iORF, uoORF, lncRNA, psORF, TrEMBL, N-Iso); smorf_label: collapsed display class (Reviewed MPs, Downstream ORF, Short-Isoform, TrEMBL/altORF, Internal ORF, lncRNA, Upstream ORF, psORF); Confidence: highest-confidence uniquely-mapped grade for the protein.

Sheet 4 (E_TrypticCapacity): Fig. 2e, theoretical tryptic-peptide capacity. **Columns:** sequence: microprotein amino acid sequence (row identifier); review_status: annotation tier ("Reviewed MPs" = Swiss-Prot, manually curated; "Unreviewed MPs" = TrEMBL plus novel transcriptome-reconstructed smORFs); n_theoretical: number of unique tryptic peptides predicted from the sequence; capacity: binned capacity (0, 1, 2, 3, 4+).

Sheet 5 (F_TrypticSupport): Fig. 2f, observed tryptic-peptide support. **Columns:** sequence: microprotein amino acid sequence (row identifier); review_status: annotation tier ("Reviewed MPs" = Swiss-Prot, manually curated; "Unreviewed MPs" = TrEMBL plus novel transcriptome-reconstructed smORFs); Confidence: confidence grade; n_pep: number of observed unique tryptic peptides; pep_group: "1 peptide" or ">=2 peptides".

File name: Source_Data_Figure3.xlsx
Description:

Sheet 1 (A_Stringent_50pct): Fig. 3a, stringent model (detected in >=50% of donors). **Columns:** sequence: microprotein amino acid sequence (row identifier); review_status: annotation tier ("Reviewed MPs" = Swiss-Prot, manually curated; "Unreviewed MPs" = TrEMBL plus novel transcriptome-reconstructed smORFs); gene_symbol: host/parent gene symbol; smorf_type: fine-grained smORF class (e.g. dORF, uORF, iORF, uoORF, lncRNA, psORF, TrEMBL, N-Iso); smorf_label: collapsed display class (Reviewed MPs, Downstream ORF, Short-Isoform, TrEMBL/altORF, Internal ORF, lncRNA, Upstream ORF, psORF); log2fc: log2 fold change, SymAD vs NonAD (TMT-MS); qvalue: Benjamini-Hochberg FDR; sig_tier: "FDR < 0.05", "FDR 0.05-0.2", or "NS"; n_detected: number of donors in which the microprotein was quantified.

Sheet 2 (B_NotStringent_all): Fig. 3b, relaxed model (>=1 donor per condition). **Columns:** sequence: microprotein amino acid sequence (row identifier); review_status: annotation tier ("Reviewed MPs" = Swiss-Prot, manually curated; "Unreviewed MPs" = TrEMBL plus novel transcriptome-reconstructed smORFs); gene_symbol: host/parent gene symbol; smorf_type: fine-grained smORF class (e.g. dORF, uORF, iORF, uoORF, lncRNA, psORF, TrEMBL, N-Iso); smorf_label: collapsed display class (Reviewed MPs, Downstream ORF, Short-Isoform, TrEMBL/altORF, Internal ORF, lncRNA, Upstream ORF, psORF); log2fc: log2 fold change, SymAD vs NonAD (TMT-MS); qvalue: Benjamini-Hochberg FDR; sig_tier: "FDR < 0.05", "FDR 0.05-0.2", or "NS"; n_detected: number of donors in which the microprotein was quantified.

File name: Source_Data_Figure4.xlsx
Description:

Sheet 1 (B_smORF_Type_DESeq_sig): Fig. 4b, dysregulated smORF types (FDR<0.05). **Columns:** sequence: microprotein amino acid sequence (row identifier);

review_status: annotation tier ("Reviewed MPs" = Swiss-Prot, manually curated; "Unreviewed MPs" = TrEMBL plus novel transcriptome-reconstructed smORFs); gene_symbol: host/parent gene symbol; smorf_type: fine-grained smORF class (e.g. dORF, uORF, iORF, uoORF, lncRNA, psORF, TrEMBL, N-Iso); smorf_label: collapsed display class (Reviewed MPs, Downstream ORF, Short-Isoform, TrEMBL/altORF, Internal ORF, lncRNA, Upstream ORF, psORF); rosmapRNA_log2FoldChange: DESeq2 log2 fold change, SymAD vs NonAD (ROSMAP RNA); rosmapRNA_padj: DESeq2 Benjamini-Hochberg FDR; is_decoupled: TRUE if regulated independently of the parent mORF (additive LRT P<0.05).

Sheet 2 (C_Volcano_DownstreamORF): Fig. 4c, per-smORF-class volcano (Downstream ORF). **Columns:** sequence: microprotein amino acid sequence (row identifier); review_status: annotation tier ("Reviewed MPs" = Swiss-Prot, manually curated; "Unreviewed MPs" = TrEMBL plus novel transcriptome-reconstructed smORFs); gene_symbol: host/parent gene symbol; smorf_type: fine-grained smORF class (e.g. dORF, uORF, iORF, uoORF, lncRNA, psORF, TrEMBL, N-Iso); smorf_label: collapsed display class (Reviewed MPs, Downstream ORF, Short-Isoform, TrEMBL/altORF, Internal ORF, lncRNA, Upstream ORF, psORF); rosmapRNA_log2FoldChange: DESeq2 log2 fold change, SymAD vs NonAD (ROSMAP RNA); rosmapRNA_padj: DESeq2 Benjamini-Hochberg FDR; point_class: "ns", "sig" (FDR<0.05), or "decoupled_sig" (FDR<0.05 and decoupled); is_decoupled: TRUE if regulated independently of the parent mORF (additive LRT P<0.05).

Sheet 3 (D_Volcano_ShortIsoform): Fig. 4d, per-smORF-class volcano (Short-Isoform). **Columns:** sequence: microprotein amino acid sequence (row identifier); review_status: annotation tier ("Reviewed MPs" = Swiss-Prot, manually curated; "Unreviewed MPs" = TrEMBL plus novel transcriptome-reconstructed smORFs); gene_symbol: host/parent gene symbol; smorf_type: fine-grained smORF class (e.g. dORF, uORF, iORF, uoORF, lncRNA, psORF, TrEMBL, N-Iso); smorf_label: collapsed display class (Reviewed MPs, Downstream ORF, Short-Isoform, TrEMBL/altORF, Internal ORF, lncRNA, Upstream ORF, psORF); rosmapRNA_log2FoldChange: DESeq2 log2 fold change, SymAD vs NonAD (ROSMAP RNA); rosmapRNA_padj: DESeq2 Benjamini-Hochberg FDR; point_class: "ns", "sig" (FDR<0.05), or "decoupled_sig" (FDR<0.05 and decoupled); is_decoupled: TRUE if regulated independently of the parent mORF (additive LRT P<0.05).

Sheet 4 (E_Volcano_TrEMBL_altORF): Fig. 4e, per-smORF-class volcano (TrEMBL/altORF). **Columns:** sequence: microprotein amino acid sequence (row identifier); review_status: annotation tier ("Reviewed MPs" = Swiss-Prot, manually curated; "Unreviewed MPs" = TrEMBL plus novel transcriptome-reconstructed smORFs); gene_symbol: host/parent gene symbol; smorf_type: fine-grained smORF class (e.g. dORF, uORF, iORF, uoORF, lncRNA, psORF, TrEMBL, N-Iso); smorf_label: collapsed display class (Reviewed MPs, Downstream ORF, Short-Isoform, TrEMBL/altORF, Internal ORF, lncRNA, Upstream ORF, psORF); rosmapRNA_log2FoldChange: DESeq2 log2 fold change, SymAD vs NonAD (ROSMAP RNA); rosmapRNA_padj: DESeq2 Benjamini-Hochberg FDR; point_class: "ns", "sig" (FDR<0.05), or "decoupled_sig" (FDR<0.05 and decoupled); is_decoupled: TRUE if regulated independently of the parent mORF (additive LRT P<0.05).

Sheet 5 (F_Volcano_lncRNA): Fig. 4f, per-smORF-class volcano (lncRNA). **Columns:** sequence: microprotein amino acid sequence (row identifier); review_status: annotation tier ("Reviewed MPs" = Swiss-Prot, manually curated; "Unreviewed MPs" = TrEMBL plus novel transcriptome-reconstructed smORFs); gene_symbol: host/parent gene symbol; smorf_type: fine-grained smORF class (e.g.

dORF, uORF, iORF, uoORF, lncRNA, psORF, TrEMBL, N-Iso); smorf_label: collapsed display class (Reviewed MPs, Downstream ORF, Short-Isoform, TrEMBL/altORF, Internal ORF, lncRNA, Upstream ORF, psORF); rosmapRNA_log2FoldChange: DESeq2 log2 fold change, SymAD vs NonAD (ROSMAP RNA); rosmapRNA_padj: DESeq2 Benjamini-Hochberg FDR; point_class: "ns", "sig" (FDR<0.05), or "decoupled_sig" (FDR<0.05 and decoupled); is_decoupled: TRUE if regulated independently of the parent mORF (additive LRT P<0.05).

Sheet 6 (G_Volcano_InternalORF): Fig. 4g, per-smORF-class volcano (Internal ORF). **Columns:** sequence: microprotein amino acid sequence (row identifier); review_status: annotation tier ("Reviewed MPs" = Swiss-Prot, manually curated; "Unreviewed MPs" = TrEMBL plus novel transcriptome-reconstructed smORFs); gene_symbol: host/parent gene symbol; smorf_type: fine-grained smORF class (e.g. dORF, uORF, iORF, uoORF, lncRNA, psORF, TrEMBL, N-Iso); smorf_label: collapsed display class (Reviewed MPs, Downstream ORF, Short-Isoform, TrEMBL/altORF, Internal ORF, lncRNA, Upstream ORF, psORF); rosmapRNA_log2FoldChange: DESeq2 log2 fold change, SymAD vs NonAD (ROSMAP RNA); rosmapRNA_padj: DESeq2 Benjamini-Hochberg FDR; point_class: "ns", "sig" (FDR<0.05), or "decoupled_sig" (FDR<0.05 and decoupled); is_decoupled: TRUE if regulated independently of the parent mORF (additive LRT P<0.05).

Sheet 7 (H_Volcano_UpstreamORF): Fig. 4h, per-smORF-class volcano (Upstream ORF). **Columns:** sequence: microprotein amino acid sequence (row identifier); review_status: annotation tier ("Reviewed MPs" = Swiss-Prot, manually curated; "Unreviewed MPs" = TrEMBL plus novel transcriptome-reconstructed smORFs); gene_symbol: host/parent gene symbol; smorf_type: fine-grained smORF class (e.g. dORF, uORF, iORF, uoORF, lncRNA, psORF, TrEMBL, N-Iso); smorf_label: collapsed display class (Reviewed MPs, Downstream ORF, Short-Isoform, TrEMBL/altORF, Internal ORF, lncRNA, Upstream ORF, psORF); rosmapRNA_log2FoldChange: DESeq2 log2 fold change, SymAD vs NonAD (ROSMAP RNA); rosmapRNA_padj: DESeq2 Benjamini-Hochberg FDR; point_class: "ns", "sig" (FDR<0.05), or "decoupled_sig" (FDR<0.05 and decoupled); is_decoupled: TRUE if regulated independently of the parent mORF (additive LRT P<0.05).

Sheet 8 (I_Volcano_psORF): Fig. 4i, per-smORF-class volcano (psORF). **Columns:** sequence: microprotein amino acid sequence (row identifier); review_status: annotation tier ("Reviewed MPs" = Swiss-Prot, manually curated; "Unreviewed MPs" = TrEMBL plus novel transcriptome-reconstructed smORFs); gene_symbol: host/parent gene symbol; smorf_type: fine-grained smORF class (e.g. dORF, uORF, iORF, uoORF, lncRNA, psORF, TrEMBL, N-Iso); smorf_label: collapsed display class (Reviewed MPs, Downstream ORF, Short-Isoform, TrEMBL/altORF, Internal ORF, lncRNA, Upstream ORF, psORF); rosmapRNA_log2FoldChange: DESeq2 log2 fold change, SymAD vs NonAD (ROSMAP RNA); rosmapRNA_padj: DESeq2 Benjamini-Hochberg FDR; point_class: "ns", "sig" (FDR<0.05), or "decoupled_sig" (FDR<0.05 and decoupled); is_decoupled: TRUE if regulated independently of the parent mORF (additive LRT P<0.05).

Sheet 9 (M_CrossCohort_Concordance): Fig. 4m, ROSMAP vs MSBB co-expression concordance. **Columns:** pair_id: smORF:mORF transcript pair identifier; r_rosmap: Pearson r (smORF vs mORF) in ROSMAP; r_msbb: Pearson r (smORF vs mORF) in MSBB; color_class: "strong" or "weak" co-expression.

File name: Source_Data_Figure5.xlsx
Description:

Sheet 1 (B_RBP_Enrichment_byType): Fig. 5b, RBP motif enrichment at smORF splice junctions. **Columns:** group: smORF class (Downstream, Isoform, Overlapping, Upstream); rbp: RNA-binding-protein recognition motif tested; a: smORF-class junctions with the motif (2x2 contingency cell a); b: smORF-class junctions without the motif (cell b); c: background junctions with the motif (cell c); d: background junctions without the motif (cell d); rate_in: motif rate among smORF-class junctions, a/(a+b); rate_bg: motif rate among background junctions, c/(c+d); odds: odds ratio (enrichment); p: two-sided Fisher's exact test p-value; fdr: Benjamini-Hochberg FDR within the smORF class.

File name: Source_Data_Figure6.xlsx
Description:

Sheet 1 (D_GO_CellularComponent): Fig. 6d, GO over-representation, Cellular Component. **Columns:** panel: figure panel letter (D/E/F); ontology: GO domain (CC, BP, MF); group: diagnosis network (NonAD or SymAD); GO_ID: Gene Ontology term identifier; description: GO term name; GeneRatio: gene ratio as a "k/n" string; gene_ratio: numeric gene ratio (plot x-axis); Count: number of network genes in the term (dot size); FoldEnrichment: fold enrichment over background; pvalue: hypergeometric test p-value; p.adjust: Benjamini-Hochberg-adjusted p-value; qvalue: q-value; geneID: member genes driving the term (slash-separated).

Sheet 2 (E_GO_BiologicalProcess): Fig. 6e, GO over-representation, Biological Process. **Columns:** panel: figure panel letter (D/E/F); ontology: GO domain (CC, BP, MF); group: diagnosis network (NonAD or SymAD); GO_ID: Gene Ontology term identifier; description: GO term name; GeneRatio: gene ratio as a "k/n" string; gene_ratio: numeric gene ratio (plot x-axis); Count: number of network genes in the term (dot size); FoldEnrichment: fold enrichment over background; pvalue: hypergeometric test p-value; p.adjust: Benjamini-Hochberg-adjusted p-value; qvalue: q-value; geneID: member genes driving the term (slash-separated).

Sheet 3 (F_GO_MolecularFunction): Fig. 6f, GO over-representation, Molecular Function. **Columns:** panel: figure panel letter (D/E/F); ontology: GO domain (CC, BP, MF); group: diagnosis network (NonAD or SymAD); GO_ID: Gene Ontology term identifier; description: GO term name; GeneRatio: gene ratio as a "k/n" string; gene_ratio: numeric gene ratio (plot x-axis); Count: number of network genes in the term (dot size); FoldEnrichment: fold enrichment over background; pvalue: hypergeometric test p-value; p.adjust: Benjamini-Hochberg-adjusted p-value; qvalue: q-value; geneID: member genes driving the term (slash-separated).

Sheet 4 (DEF_MKKS_Correlations): Input to Fig. 6d-f, genes co-expressed with Micro-MKKS63 (smORF CDS). **Columns:** group: NonAD or SymAD network; gene: gene symbol; ensembl_gene: Ensembl gene ID; correlation: Pearson r of the gene vs Micro-MKKS63 (smORF CDS); abs_corr: absolute value of correlation; GO_over_R_0.7: "Yes" if |r|>0.7 (included in the GO analysis), else "No".

Sheet 5 (G_MKKS_mORF_Correlation): Fig. 6g, genes co-expressed with the canonical MKKS mORF (Q9NPJ1). **Columns:** group: NonAD or SymAD network; gene: gene symbol; ensembl_gene: Ensembl gene ID; correlation: Pearson r of the gene vs the canonical MKKS mORF (Q9NPJ1); abs_corr: absolute value of correlation; GO_over_R_0.7: "Yes" if |r|>0.7 (included in the GO analysis), else "No".

File name: Source_Data_Figure7.xlsx
Description:

Sheet 1 (F_OMM_Volcano): Fig. 7f, outer-mitochondrial-membrane (OMM) APEX proteomics enrichment volcano, mito-FL vs untransduced (one row per tested

protein). **Columns**: ProteinName: UniProt-style protein identifier (Micro-MKKS63 / uORF-MKKS = tr|Q9HB66|Q9HB66_HUMAN); log2FC: log2 fold-change of protein abundance (mito-FL − untransduced; volcano x-axis); log2FC_CI_low: lower bound of the 95% CI on log2FC; log2FC_CI_high: upper bound of the 95% CI on log2FC; pval: two-sided Welch t-test p-value on log2 intensity; FDR: Benjamini–Hochberg adjusted p-value across all tested proteins; negLog10FDR: −log10(FDR) (volcano y-axis); is_MicroMKKS63: TRUE for the Micro-MKKS63 (uORF-MKKS) protein row.
Sheet 2 (G_OCR_Timecourse): Fig. 7g, Seahorse OCR time course (one row per well x timepoint). **Columns:** group: NTC (non-targeting control), Micro-MKKS63(1) or Micro-MKKS63(2) knockout line; Time: assay time (minutes); well_id: Seahorse plate well identifier; OCR: oxygen consumption rate (pmol/min).
Sheet 3 (H_Basal_OCR): Fig. 7h, basal OCR (one row per well). **Columns:** group: NTC (non-targeting control), Micro-MKKS63(1) or Micro-MKKS63(2) knockout line; basal_ocr: basal OCR per well (pmol/min).
Sheet 4 (I_Max_Capacity): Fig. 7i, maximal respiration (one row per well). **Columns:** group: NTC (non-targeting control), Micro-MKKS63(1) or Micro-MKKS63(2) knockout line; max_capacity: maximal respiration per well (pmol/min).
Sheet 5 (J_ATP_Linked_OCR): Fig. 7j, ATP-linked OCR (one row per well). **Columns:** group: NTC (non-targeting control), Micro-MKKS63(1) or Micro-MKKS63(2) knockout line; atp_linked_ocr: ATP-linked OCR per well (pmol/min).
Sheet 6 (K_Reserve_OCR): Fig. 7k, reserve capacity (one row per well). **Columns:** group: NTC (non-targeting control), Micro-MKKS63(1) or Micro-MKKS63(2) knockout line; reserver_capacity: reserve capacity per well (pmol/min).

File name: Source_Data_Figure8.xlsx
Description:
Sheet 1 (F_Actin_Distribution): Fig. 8f, edge-to-center radial F-actin profile (one row per cell x bin). **Columns:** group: EV (empty vector) or ACTBP7-psORF; electroporation_experiment: transfection batch / biological replicate; cell: cell identifier; Bin: radial bin index (edge to center); Average: mean F-actin intensity in that radial bin.
Sheet 2 (G_WholeCell_Actin): Fig. 8g, whole-cell F-actin (one row per cell). **Columns:** group: EV (empty vector) or ACTBP7-psORF; electroporation_experiment: transfection batch / biological replicate; cell: cell identifier; AverageCellActin: mean whole-cell F-actin intensity (arbitrary units).
Sheet 3 (H_Nuclear_Actin): Fig. 8h, nuclear F-actin (one row per cell). **Columns:** group: EV (empty vector) or ACTBP7-psORF; electroporation_experiment: transfection batch / biological replicate; cell: cell identifier; AverageNuclearActin: mean nuclear F-actin intensity (arbitrary units).
Sheet 4 (I_NucToCell_Ratio): Fig. 8i, nuclear-to-whole-cell F-actin ratio (one row per cell). **Columns:** group: EV (empty vector) or ACTBP7-psORF; electroporation_experiment: transfection batch / biological replicate; cell: cell identifier; NucToCellRatio: nuclear / whole-cell F-actin intensity ratio.

File name: Source_Data_Extended_Data_Figure1.xlsx
Description:
Sheet 1 (PROSIT_Grading): Extended Data Fig. 1, manual vs PROSIT spectral grading (50 spectra x 2 reviewers). **Columns:** tryptic_peptide: peptide whose spectrum was graded; PROSIT_grade: spectral-angle-derived tier (reference); User_grade: manually assigned tier by the reviewer; User_ID: reviewer identifier (1 or 2).

File name: Source_Data_Extended_Data_Figure2.xlsx
Description:

Sheet 1 (PhyloCSF): Extended Data Fig. 2, PhyloCSF evolutionary conservation. **Columns:** sequence: microprotein amino acid sequence (row identifier); review_status: annotation tier ("Reviewed MPs" = Swiss-Prot, manually curated; "Unreviewed MPs" = TrEMBL plus novel transcriptome-reconstructed smORFs); gene_symbol: host/parent gene symbol; smorf_type: fine-grained smORF class (e.g. dORF, uORF, iORF, uoORF, lncRNA, psORF, TrEMBL, N-Iso); mean_phylocsf: mean PhyloCSF conservation score; max_phylocsf: maximum PhyloCSF score across frames/windows.

File name: Source_Data_Extended_Data_Figure3.xlsx
Description:

Sheet 1 (QuantificationRate): Extended Data Fig. 3, TMT cumulative quantification-rate (eCDF). **Columns:** sequence: microprotein amino acid sequence (row identifier); review_status: annotation tier ("Reviewed MPs" = Swiss-Prot, manually curated; "Unreviewed MPs" = TrEMBL plus novel transcriptome-reconstructed smORFs); n_control: number of Control samples with a quantified value; n_asymad: number of AsymAD samples quantified; n_ad: number of AD (SymAD) samples quantified; n_total_detected: number of samples with any detection (of 488); quantification_rate_pct: percent of samples in which the microprotein was quantified.

File name: Source_Data_Extended_Data_Figure4.xlsx
Description:

Sheet 1 (SwissProt_TMT_Volcano): Extended Data Fig. 4, Swiss-Prot protein TMT differential-expression volcano (one row per protein). **Columns:** gene_symbol: protein/gene symbol; Database: source database (Swiss-Prot); TMT_log2fc: log2 fold change, AD vs Control (TMT-MS); TMT_qvalue: Benjamini-Hochberg FDR (q-value); neg_log10_TMT_qvalue: -log10(q) (volcano y-axis); significant: TRUE if q <= 0.20 (plotted threshold), else FALSE.

File name: Source_Data_Extended_Data_Figure5.xlsx
Description:

Sheet 1 (B_MotifSharing_perPair): Extended Data Fig. 5b, promoter TF-motif sharing (one row per smORF-parent pair). **Columns:** smorf_id: smORF transcript identifier; parent_gene: parent (main-ORF) gene symbol; smorf_type: fine-grained smORF class (e.g. dORF, uORF, iORF, uoORF, lncRNA, psORF, TrEMBL, N-Iso); amino_acid_sequence: microprotein amino acid sequence; n_smorf_motifs: number of transcription-factor motifs in the smORF promoter; n_parent_motifs: number of TF motifs in the parent (main-ORF) promoter; n_shared: number of motifs shared between smORF and parent promoters; n_unique_to_smorf: number of motifs present only in the smORF promoter; n_unique_to_parent: number of motifs present only in the parent promoter.

File name: Source_Data_Extended_Data_Figure6.xlsx
Description:

Sheet 1 (MKKS_PeptideCentric): Extended Data Fig. 6, PepCentric peptide-level PSM coverage on MKKS. **Columns:** Protein: "Main MKKS ORF" (canonical Q9NPJ1) or "uORF-Microprotein on MKKS" (Micro-MKKS63); Peptide: tryptic peptide sequence; start_position: peptide start position in the protein; end_position: peptide

end position in the protein; PSM_Count: number of PSMs across the 66,700-run PepCentric meta-database; EvidenceType: evidence type (MS).

File name: Source_Data_Extended_Data_Figure7.xlsx
Description:

Sheet 1 (A_Nanopore_Volcano): Extended Data Fig. 7a, psORF long-read RNA differential-expression volcano (one row per transcript). **Columns:** sequence: microprotein amino acid sequence (row identifier); review_status: annotation tier ("Reviewed MPs" = Swiss-Prot, manually curated; "Unreviewed MPs" = TrEMBL plus novel transcriptome-reconstructed smORFs); gene_symbol: host/parent gene symbol; smorf_type: fine-grained smORF class (e.g. dORF, uORF, iORF, uoORF, lncRNA, psORF, TrEMBL, N-Iso); parent_gene: parent (main-ORF) gene symbol; parent_category: "Actin-Cytoskeleton psORF-MPs", "Non-Actin-Cytoskeleton psORF-MPs", or "Other"; nanopore_log2FoldChange: DESeq2 log2 fold change, AD vs control (long-read RNA); nanopore_pvalue: DESeq2 Wald test p-value (unadjusted); neg_log10_pvalue: -log10(p) (volcano y-axis); significant: TRUE if p<0.05 and |log2FC|>1 (colored points), else FALSE.

Sheet 2 (B_ACTB_ACTG1_Boxplot): Extended Data Fig. 7b, actin-related psORF CPM by diagnosis (one row per gene x sample). **Columns:** sequence: microprotein amino acid sequence (row identifier); review_status: annotation tier ("Reviewed MPs" = Swiss-Prot, manually curated; "Unreviewed MPs" = TrEMBL plus novel transcriptome-reconstructed smORFs); gene_symbol: host/parent gene symbol; parent_gene: parent (main-ORF) gene symbol; parent_category: parent gene family category; sample: brain-sample identifier; condition: "Control" or "AD"; cpm: expression in counts-per-million (plotted as CPM + 1).

File name: Source_Data_Extended_Data_Figure8.xlsx
Description:

Sheet 1 (D_Actin_Distribution): Extended Data Fig. 8d, edge-to-center radial F-actin profile (one row per cell x bin). **Columns:** group: EV (empty vector) or ACTBP11-smORF; electroporation_experiment: transfection batch / biological replicate; cell: cell identifier; Bin: radial bin index (edge to center); Average: mean F-actin intensity in that radial bin.

Sheet 2 (E_WholeCell_Actin): Extended Data Fig. 8e, whole-cell F-actin (one row per cell). **Columns:** group: EV (empty vector) or ACTBP11-smORF; electroporation_experiment: transfection batch / biological replicate; cell: cell identifier; AverageCellActin: mean whole-cell F-actin intensity (arbitrary units).

Sheet 3 (E_Nuclear_Actin): Extended Data Fig. 8e, nuclear F-actin (one row per cell). **Columns:** group: EV (empty vector) or ACTBP11-smORF; electroporation_experiment: transfection batch / biological replicate; cell: cell identifier; AverageNuclearActin: mean nuclear F-actin intensity (arbitrary units).

Sheet 4 (E_NucToCell_Ratio): Extended Data Fig. 8e, nuclear-to-whole-cell F-actin ratio (one row per cell). **Columns:** group: EV (empty vector) or ACTBP11-smORF; electroporation_experiment: transfection batch / biological replicate; cell: cell identifier; NucToCellRatio: nuclear / whole-cell F-actin intensity ratio.
