# Supplementary material for: A microprotein atlas of the human frontal cortex in Alzheimer’s disease
Source: Nat Aging. 2026 Sep 14;6(9):1979–97. doi: 10.1038/s43587-026-01207-x (PMC13577902; doi:10.1038/s43587-026-01207-x)
Supplement: Supplementary file 2 [file 43587_2026_1207_MOESM2_ESM.pdf]

nature portfolio

Corresponding author(s): Brendan Miller, Alan Saghatelian

Last updated by author(s): 07/08/2026

Reporting Summary

Nature Portfolio wishes to improve the reproducibility of the work that we publish. This form provides structure for consistency and transparency in reporting. For further information on Nature Portfolio policies, see our Editorial Policies and the Editorial Policy Checklist.

Statistics

For all statistical analyses, confirm that the following items are present in the figure legend, table legend, main text, or Methods section.

n/a	Confirmed	
☐	☒	The exact sample size (*n*) for each experimental group/condition, given as a discrete number and unit of measurement
☐	☒	A statement on whether measurements were taken from distinct samples or whether the same sample was measured repeatedly
☐	☒	The statistical test(s) used AND whether they are one- or two-sided<br>*Only common tests should be described solely by name; describe more complex techniques in the Methods section.*
☐	☒	A description of all covariates tested
☐	☒	A description of any assumptions or corrections, such as tests of normality and adjustment for multiple comparisons
☐	☒	A full description of the statistical parameters including central tendency (e.g. means) or other basic estimates (e.g. regression coefficient) AND variation (e.g. standard deviation) or associated estimates of uncertainty (e.g. confidence intervals)
☐	☒	For null hypothesis testing, the test statistic (e.g. *F*, *t*, *r*) with confidence intervals, effect sizes, degrees of freedom and *P* value noted<br>*Give P values as exact values whenever suitable.*
☒	☐	For Bayesian analysis, information on the choice of priors and Markov chain Monte Carlo settings
☒	☐	For hierarchical and complex designs, identification of the appropriate level for tests and full reporting of outcomes
☐	☒	Estimates of effect sizes (e.g. Cohen's *d*, Pearson's *r*), indicating how they were calculated

Our web collection on statistics for biologists contains articles on many of the points above.

Software and code

Policy information about availability of computer code

Data collection	New data-independent acquisition (DIA) data were acquired on a Thermo Scientific Orbitrap Astral coupled to a Vanquish NEO LC system; HMC3 microglial cell proteomics were acquired on an Orbitrap Fusion Lumos Tribrid coupled to an Easy-nLC 1000 (Thermo Fisher Scientific). Mitochondrial respiration was measured on a Seahorse XF96 Analyzer (Agilent) with the Seahorse XF Mito Stress Test kit; raw oxygen consumption rate (OCR) traces were collected and processed in Wave software. Confocal images were acquired on a Nikon A1R laser-scanning confocal microscope using NIS-Elements (vAR.6.10.02 64-bit), generating .ND2 files.

April 2023

Data analysis

Data analysis integrated open-source software, commercial tools, and standardized proteomics workflows. Statistical analyses, data structuring, visualization, and figure generation were performed in R (v4.4.1), using DESeq2 (v1.44.0), limma (v3.60.6), dplyr (v1.1.4), ggplot2 (v4.0.0), patchwork (v1.3.2), psych (v2.6.1), ggh4x (v0.3.1), and clusterProfiler (v4.12.6) with org.Hs.eg.db for Gene Ontology enrichment. Long-read transcriptomes were aligned with minimap2 (v2.26-r1175), compressed with samtools (v1.18), sorted with sambamba (v1.0.1), and assembled with ESPRESSO (v1.0) against GENCODE v43; GTFs were sorted with GFF3sort and three-frame translated with GTFtoFASTA. TMT proteomic data were searched with FragPipe (v20.0), which integrates MSFragger (v22.0), Philosopher (v5.0), and IonQuant (v1.8.5), with Percolator rescoring via MSBooster. smORF annotation used bedtools (v2.30.0) and BLASTP. Ribosome profiling was processed with the custom RP3 framework using STAR (v2.7.4a), the FastX Toolkit (v0.0.13), TransDecoder (v5.7.1), RibORF, RiboCode, and PRICE; translated smORFs were classified with the ShortStop machine-learning classifier. DIA data were analyzed with DIA-NN (v2.2.0). Spectral validation used PROSIT (Prosit_2020_intensity_TMT) via the Koina API with pyteomics for spectrum retrieval. Batch normalization used TAMPOR (https://github.com/edammer/TAMPOR). Short-read RNA-seq used STAR (v2.7.10a), featureCounts (Subread v2.0.1), and DESeq2 (v1.44.0). Transcription factor and RNA-binding protein motif analyses used HOMER (v4.11) and FIMO (MEME Suite v5.5.9); disease associations were queried via the Open Targets Platform GraphQL API (v4). sgRNA design used CCTop and editing efficiency was quantified with TIDE. Image analysis used Fiji/ImageJ and custom Python scripts with NumPy (v2.0.2), SciPy (v1.13.1), and scikit-image (v0.24.0). Seahorse statistics were performed in GraphPad Prism (v10). All custom code is publicly available at https://github.com/brendan-miller-salk/brain-microprotein-atlas.

For manuscripts utilizing custom algorithms or software that are central to the research but not yet described in published literature, software must be made available to editors and reviewers. We strongly encourage code deposition in a community repository (e.g. GitHub). See the Nature Portfolio guidelines for submitting code & software for further information.

Data

Policy information about availability of data

All manuscripts must include a data availability statement. This statement should provide the following information, where applicable:

- Accession codes, unique identifiers, or web links for publicly available datasets
- A description of any restrictions on data availability
- For clinical datasets or third party data, please ensure that the statement adheres to our policy

This study combines newly generated datasets with publicly available multi-omic resources. Raw mass-spectrometry data generated in this study (human brain DIA and HMC3 microprotein-enrichment experiments) have been deposited in the PRIDE Archive under accession PXD071500. Oxford Nanopore long-read transcriptomic data from human dorsolateral prefrontal cortex generated by the Sanders-Brown Center on Aging (source dataset syn52047893) are available through Synapse under restricted access (https://www.synapse.org/#!Synapse:syn51923506); a free Synapse account is required. Re-analyzed ribosome profiling data (Duffy et al.) are available under dbGaP accession phs002489.v1.p1. A published BioID proximity-labelling dataset re-analyzed here is available under PRIDE accession PXD060912. Additional datasets were obtained from the AMP-AD Knowledge Portal (https://adknowledgeportal.synapse.org) under standard AMP-AD data-use terms, shared without publication embargo. ROSMAP cohort resources can be requested through the Rush Alzheimer's Disease Center (www.radc.rush.edu). The UniProtKB human proteome database (Swiss-Prot + TrEMBL; UP000005640, downloaded January 20, 2025) was used for all proteomic searches and is publicly accessible at https://ftp.uniprot.org/pub/databases/uniprot/previous_releases/.

Research involving human participants, their data, or biological material

Policy information about studies with human participants or human data. See also policy information about sex, gender (identity/presentation), and sexual orientation and race, ethnicity and racism.

Reporting on sex and gender

Sex was recorded from de-identified clinical and demographic records for all cohorts (ROSMAP, MSBB, Sanders-Brown, Duffy et al., and UCSD), each of which included both male and female participants at proportions reported and was included as a covariate in relevant differential expression and proteomic models.

Reporting on race, ethnicity, or other socially relevant groupings

The manuscript controls for race when captured by respective cohort's metadata through Synapse, following harmonized analytic workflows from previous publications using this data.

Population characteristics

Across cohorts, the ROSMAP TMT proteomics dataset comprised 608 participants (69.1% female; mean age 87.8 ± 4.4 years) across two acquisition rounds (Round 1, n=400, 70.2% female, 87.5 ± 4.6 years; Round 2, n=208, 66.8% female, 88.3 ± 3.9 years), of which 480 were retained after diagnostic classification (69.8% female; 87.9 ± 4.3 years: NonAD n=111, 58.6% female, 85.4 ± 5.3 years; AsymAD n=182, 72.5% female, 88.2 ± 3.9 years; SymAD n=187, 73.8% female, 89.1 ± 3.2 years). The matched ROSMAP bulk RNA-Seq cohort included 373 participants (66.2% female; 88.0 ± 4.4 years: NonAD n=90, 54.4% female, 85.1 ± 5.4 years; AsymAD n=141, 69.5% female, 88.5 ± 3.9 years; SymAD n=142, 70.4% female, 89.2 ± 3.1 years). The MSBB replication cohort comprised 258 participants (64.3% female; 83.8 ± 8.0 years: NonAD n=57, 49.1% female, 79.1 ± 9.5 years; AsymAD n=22, 77.3% female, 87.8 ± 4.9 years; SymAD n=179, 67.6% female, 84.8 ± 7.2 years). The Sanders-Brown long-read RNA-Seq cohort included 12 participants (50% female; 84.4 ± 6.2 years; 6 AD, 6 control, each 50% female, mean ages 86.2 ± 4.4 and 82.7 ± 7.6 years, respectively). The Duffy et al. Ribo-seq cohort, used for translational re-analysis rather than diagnostic comparison, included 43 adult participants (41.9% female; 39.6 ± 14.7 years). Finally, the UCSD DIA proteomics cohort comprised 4 participants (mean age 82.5 years; male; two AD, one amnestic MCI, one cognitively normal).

Recruitment

All research participants and postmortem brain donors provided written informed consent prior to enrollment.

Ethics oversight

Cohorts received ethical approval from respective Institutional Review Boards as previously reported.

Note that full information on the approval of the study protocol must also be provided in the manuscript.

Field-specific reporting

Please select the one below that is the best fit for your research. If you are not sure, read the appropriate sections before making your selection.

☒ Life sciences ☐ Behavioural & social sciences ☐ Ecological, evolutionary & environmental sciences

For a reference copy of the document with all sections, see nature.com/documents/nr-reporting-summary-flat.pdf

Life sciences study design

All studies must disclose on these points even when the disclosure is negative.

Sample size	No sample size calculations were performed. Data from samples with brain proteomic, transcriptomic, and phenotypic data were included that passed quality control.
Data exclusions	To assess global structure and detect outliers in proteomic data, we performed principal component analysis (PCA) on the normalized protein expression matrix. Outliers were identified based on extreme deviation along the first two principal components (z score absolute value greater than 3). Transcriptomic analyses were carried out only on matched proteomics donor tissues.
Replication	For ROS and MAP data, external replication across independent cohorts was not performed for proteomics studies but was performed for transcriptomics studies using the the MSBB study. Identical preprocessing, normalization, and modeling steps across all samples ROS and MAP was applied as reported. For nanopore long transcriptome analysis, no similar data set is available. Functional experiments to assess Micro-MKKS63 knockout and psORF cytoskeleton was replicated three times; two individual polyclonal cell lines derived from two independent sgRNA sequences targeting the Micro-MKKS63 smORF.
Randomization	Original samples were randomized prior to data collection.
Blinding	No interventions were given.

Reporting for specific materials, systems and methods

We require information from authors about some types of materials, experimental systems and methods used in many studies. Here, indicate whether each material, system or method listed is relevant to your study. If you are not sure if a list item applies to your research, read the appropriate section before selecting a response.

Materials & experimental systems

n/a	Involved in the study	
☐	☒	Antibodies
☐	☒	Eukaryotic cell lines
☒	☐	Palaeontology and archaeology
☒	☐	Animals and other organisms
☒	☐	Clinical data
☒	☐	Dual use research of concern
☒	☐	Plants

Methods

n/a	Involved in the study	
☒	☐	ChIP-seq
☒	☐	Flow cytometry
☒	☐	MRI-based neuroimaging

Antibodies

Antibodies used	For immunocytochemistry and western blotting, several primary antibodies were used, including mouse anti-FLAG M2 (Sigma-Aldrich F1804), rabbit anti-Mitofilin (Proteintech 10179-1-AP), rabbit anti-ALFA polyclonal antibody (NanoTag N1581), and the FluoTag-X2 anti-ALFA 568 reagent (NanoTag N1502). Secondary detection relied on anti-mouse Jackson Alexa Fluor 488 (115-545-003) and anti-rabbit Alexa Fluor 594 (111-585-003) antibodies from Jackson ImmunoResearch. DAPI was used for nuclear staining, and all fixed-cell preparations were mounted using Ibidi antifade mounting medium (50011).
Validation	Commercial antibodies used in this study were selected based on vendor-provided validation data generated using recombinant protein standards and gene knockout models.

Eukaryotic cell lines

Policy information about cell lines and Sex and Gender in Research

Cell line source(s)	Human Microglia Clone 3; CHME-3; CHME3; HMC3, CRL-3304 (ATCC), sex unspecified, embryo age
Authentication	Cell lines were obtained from commercial sources and were not independently authenticated.

Mycoplasma contamination	Cell line testing verified for no mycoplasma contamination.
Commonly misidentified lines (See ICLAC register)	NA

Plants

Seed stocks	*Report on the source of all seed stocks or other plant material used. If applicable, state the seed stock centre and catalogue number. If plant specimens were collected from the field, describe the collection location, date and sampling procedures.*
Novel plant genotypes	*Describe the methods by which all novel plant genotypes were produced. This includes those generated by transgenic approaches, gene editing, chemical/radiation-based mutagenesis and hybridization. For transgenic lines, describe the transformation method, the number of independent lines analyzed and the generation upon which experiments were performed. For gene-edited lines, describe the editor used, the endogenous sequence targeted for editing, the targeting guide RNA sequence (if applicable) and how the editor was applied.*
Authentication	*Describe any authentication procedures for each seed stock used or novel genotype generated. Describe any experiments used to assess the effect of a mutation and, where applicable, how potential secondary effects (e.g. second site T-DNA insertions, mosiacism, off-target gene editing) were examined.*
