# Supplementary material for: A microprotein atlas of the human frontal cortex in Alzheimer’s disease
Source: Nat Aging. 2026 Sep 14;6(9):1979–97. doi: 10.1038/s43587-026-01207-x (PMC13577902; doi:10.1038/s43587-026-01207-x)
Supplement: Supplementary file 3 [file 43587_2026_1207_MOESM3_ESM.pdf]

nature portfolio

Peer Review File

A microprotein atlas of the human frontal cortex in Alzheimer's disease

Corresponding Author: Professor Alan Saghatelian

Version 0:

Reviewer comments:

Reviewer #1

(Remarks to the Author)
In this work, the authors present a multi-omics analysis of proteins below 150 aa by TMT-MS, DIA-MS and ribo-Seq in human brains. They complement and validate some of their findings using orthogonal methods such as immunofluorescence imaging, Western Blotting, RiboSeq and mitochondrial activity assays. The results are made available as a public atlas of microproteins in the context of aging and Alzheimer's disease. The majority of the data presented in the manuscript were previously published: The TMT-MS and ribo-Seq data were published previously and now re-analyzed. While the previous publication for this TMT-MS dataset did not focus on microproteins, the reprocessing of the data presented on this manuscript follows standard or previously published workflows. The ribo-Seq data was reanalyzed using ShortStop, a framework for microprotein discovery previously published by the authors. The provided code contains sufficient information to run it, including a list of requirements and readme files. The code availability on GitHub makes it compliant to good practices on data transparency.
The study is of broad interest due to the disease context in which microproteins are yet understudied, and as such would be suitable for publication in Nature Ageing. However, in the present form, the study contains a number of limitations that need to be addressed before further consideration for publication as outlined below:

Major Issues

Specifically, the statistical analyses carried out in this work require clarification. In line 131 and 134, a $q < 0.2$ is used as a significance threshold. If this q refers to a q-value from a multiple hypothesis testing, a threshold of 0.2 is unusually high, indicating that up to 20% of the values are false-positives. Similarly, the same q-value threshold was applied at Figure 1K to claim differential regulation of BCL3-oORF and RAB3C. Using a q-value threshold for significance of 0.2 is not appropriate and does not meet minimal standards for a statistical analysis.
Also, overall fold changes are relatively small (Figure 1G). We are surprised so many proteins passed the statistical significance threshold and assume this may be due to the high q value threshold (0.2), making the analysis relatively error-prone. The authors need to redo these analyses and apply a statistical test using thresholds that are accepted in the field (such as 0.05 or 0.01).
If the authors want to include a "low-fidelity" cohort of hits with high q value thresholds that would be ok, but these results need to be labeled as such and clearly separated from results that were acquired with commonly-used q value thresholds.

Furthermore, while the combined data present in the microprotein atlas will serve as a useful and important repository for future studies, the numbers stated in the manuscript include proteins supported by only one peptide and only one peptide-spectra-match (PSM). One-hit wonders are a known limitation in studies of small proteins as it is often not possible to detect more than one peptide for many of these proteins, making it hard to distinguish them from false-hits. The authors should consider approaches to manually curate and validate these identifications by e.g. only including one-hit-wonders with high-quality peptide spectra (i.e. with fragment spectra containing at least 5 consecutive b or y ions, as suggested in previous publications from this group, e. g. Slavoff et al., 2012). Alternatively, the authors could predict retention time and fragmentation patterns of the detected microprotein-derived peptides (both canonical and non-canonical) and evaluate the overlap between the data they acquired and the predicted spectra (using e.g. PROSIT by the Wilhelm lab).
Taken together, the claim that there is a hidden layer of thousands of noncanonical microproteins (Lines 67, 137 and 514) seems exaggerated as the majority of them could not be quantified nor are supported by more than 1 PSM, without any manual curation or validation. We also strongly recommended that spectra for proteins identified by a single peptide are made available either in the online atlas or as supplemental figures.

The presentation of the data in the current manuscript needs to be improved, as indicated below. While most figures are

well-structured, a number of figure panels are wrongly cross-referenced or missing, figure legends need to be improved to describe each panel, and larger font sizes are needed in many figure panels to improve readability. In general, we also ask for higher-resolution figures to be uploaded as for example the spectra in Figure 1H cannot be assessed properly at this resolution. We also found throughout the text a few sentences that are missing a reference and a few references that cite the wrong publication (see below).

Proteomics data need to be more transparent in this publication. While a PRIDE identifier was made available throughout the review process, the online Atlas should also contain the links to the raw data.

Minor issues

Line 38 – reference missing
Line 44 – The authors need to reference their definition of microproteins as "peptides shorter than 150 amino acids". First, it is important to highlight that "proteins" would be a better term for this sentence instead of "peptide" (see PMID 24606146). Furthermore, most publications in the field of microproteins consider the threshold to be 100 amino acids.
Line 90 – Reference seems to be incorrect. Reference 30 (Bennett et al. 2018) does not contain any information about the TMT dataset.
Line 92 – The fact that canonical microproteins generate more tryptic peptides and are generally longer is not surprising considered that the limitations in microprotein detection by MS are mostly observed for proteins < 100 aa, and are strikingly high for proteins < 50 aa. This is directly in line with our comment re. line 44.
Line 102 and Fig 1C – the horizontal spread around 0 on the violin plot for noncanonical proteins is much higher than for canonical proteins. This might indicate that some of them are not expressed at all. It would be interesting to see a correlation between transcriptomics data and MS-intensity / number of peptides/PSMs (shown similarly in Fig 1D). This might help filter out false-identifications.
Line 108-109 – The sentence does not read well. Perhaps the authors intended: "... coding sequences with different coding frames..."
Line 128 – We recommend reviewing and clarifying figure legends. Is Extended Data Fig 2D distinguishing canonical and noncanonical microproteins? While it feels intuitive given the consistent colour choices throughout the text, an actual clarification in the legends would be recommended.
Line 129 – It would be interesting to see an overlap between canonical and noncanonical microproteins in Figure 1G. We would also appreciate a comment from the authors on the observed fold-changes. -0.2 to +0.2 log2-fold changes seem very small. The spread of the volcano-plot in Figure 1G is also misleading, the width makes the fold-changes seem much bigger than they actually are.
Line 132 – Did the authors investigate why only 4.6% of the noncanonical microproteins (according to Extended Fig 2D) were found in >50% of the samples? This number is low compared to 37.6% for canonical microproteins. Are there any group of microproteins found consistently in only one group?
Also, these numbers may indicate that the majority of the noncanonical microproteins may be false-positive hits.
Figure 1A – For clarity, the authors should state the meaning of Asym-AD and Sym-AD. While intuitive, it is important that these abbreviations are noted in the figure legend.
Figure 1H – The spectra would be more meaningful if added to the extended figures in larger sizes. In this panel, they are simply too small to be properly interpreted and the axis labels are unreadable.
Line 167-169 – Several reviews are available in literature on the difficulties of detecting small and microproteins by MS. It would be relevant to cite at least one.
Line 172 (and other occurences) – The authors should reference here the original publication from which the Ribo-Seq data comes from.
Line 173 - if this homology independent algorithm is ShortStop, this should be noted here, instead of in line 183. The initial sentences of this paragraph and the next one seem repetitive.
Line 182 – It would be interesting to visualize the number of predicted tryptic peptides for these 1607 proteins in Extended Figure 1. In our experience, it is not unusual to find microproteins with 0 tryptic cleavage sites.
Line 182 – The entire paragraph is confusing. After multiple times reading it, it remains unclear if the panels in Figure 2 are wrongly labelled or if the cross-references in the text are referring to the wrong panels. In addition to checking the cross-references, we suggest an improvement in the sentence's clarity.
Line 195 – Do the authors mean "Undetected microproteins may fall outside..."? Could this indicate that some of these are simply false identifications? If they have low ribosome occupancy and no MS-data, there is not enough support for their expression.
Line 198 – As above, is this referring to Fig 2C?
Line 204 – typo in analysis
Line 207 – the authors should reference at least one of the studies showing that many microproteins originate de novo and are less conserved.
Line 212 and 217 – Panels F and G are missing, or wrongly cross-referenced.
Line 249 – The authors claim that at the peptide level, microproteins that map to neuronal-associative genes were lower abundance than canonical Swiss-Prot proteins (Fig 3C). This however was showed for the overall microproteome in Figure 1D. Did the authors compared the noncanonical neuronal-associative microproteins from Fig 3C, to a background of all other noncanonical microproteins to see if there was a difference?
Line 249 – Same as above – Figure 3C seem to indicate that non canonical small proteins have larger Log10PSM values than SwissProt for most cell types. This is conflicting to the claim in the text.
Line 251 – For astrocytes, the Log2-FC seem too heterogeneous to support the claim that they are generally upregulated in AD.

Line 280 – reference missing.
Line 284 – reference to RP3 missing.
Fig 6A – We recommend including an y-axis for the ribo-seq data to enable to visualization of the signal intensity.
Line 384 – can the authors comment on the biological significance of a -0.06 log2-fold change in the context of the project? While significant, this difference seem way too small to be meaningful.
Line 629 – Paragraph is duplicated.
Line 672 – Please highlight the number of sequences present in the downloaded UniProt proteome, and the date in which it was downloaded.
Line 708 – A reference for the RP3 pipeline is missing.
A few comments on the Streamlit App. These are not critical but might be considered as improvement points for next updates on the platform.

- When downloading a CSV, the emojis used in the web platform are downloaded as unformatted code, which makes sorting/filtering in excel or R a bit more complicated (e.g. “No” is viewed in excel as “âŒ No”.
- In the general noncanonical explorer, it would be interesting to see the fold-changes observed in TMT-MS / RNA instead of simply Yes/No.
- In the detailed individual analysis hub, when you have a large number of entries in the table, but are displaying less rows (e.g. showing 4,321 entries but displaying only 50 rows), any attempt to sort the table by a column only affects the 50 visible rows. For example, if I sort by TMT_log2foldchanges, it sorts only for those 50 values and it remains unclear how it looks for the rest.

(Remarks on code availability)
The provided code contains sufficient information to run it, including a list of requirements and readme files. The code availability on GitHub makes it compliant to good practices on data transparency.

Reviewer #2

(Remarks to the Author)
I co-reviewed this manuscript with one of the reviewers who provided the listed reports. This is part of the Nature Aging initiative to facilitate training in peer review and to provide appropriate recognition for Early Career Researchers who co-review manuscripts.

(Remarks on code availability)

Reviewer #3

(Remarks to the Author)
This manuscript presents an atlas of unannotated microproteins in the human brain, utilizing a multi-omics workflow to characterize these proteins in the context of Alzheimer's disease (AD). The authors integrated long-read transcriptomics, Ribo-Seq, and quantitative proteomics (TMT-MS) across more than 480 dorsal lateral prefrontal cortex samples to identify 3,217 noncanonical microproteins absent from standard reference databases. The authors find that several microproteins that are differentially expressed in AD are regulated independently of their canonical parent genes (e.g., EMC10-dORF, UBA1-uORF), indicating disease-specific regulatory mechanisms. The authors report that 215 loci annotated as "pseudogenes" are translated in the human brain and that there is coordinated repression of 13 actin-related pseudogenes in AD.
The most rigorously supported section of this study is in figures 5 and 6, where the authors claim that uORF1 is the dominant product of the MKKS locus, localizes to mitochondria, and controls mitochondrial respiration. The authors demonstrate a clear distinction between the gene locus (MKKS) and its microproteins. They show that while MKKS is a canonical gene, its primary output in the brain is the noncanonical uORF1 microprotein. The use of the "PepCentric" meta-database to show the absence of canonical MKKS across 66,700 public experiments provides strong external validity. Additionally, the controls for the CRISPR experiments are strong and the 40-50% reduction in respiration is biologically significant. As a whole, this manuscript nicely integrates multiple techniques (long-read RNA-seq, TMT-MS, Ribo-Seq, DIA-MS). The data quality is high, with clear MS/MS spectra, and immunofluorescence imaging data provided. Furthermore, the reporting is transparent, methods are detailed, and data and analysis code have been made available via public repositories.
This study is the first large-scale atlas of noncanonical microproteins in the aged and AD human brain and it provides a robust workflow for discovering noncanonical proteins in complex human tissues. The results are of interest to neurobiologists, genomics and proteomics researchers. The manuscript can be further strengthened after addressing the following comments:

1) The core of the study is the creation of a “high-quality” atlas of 3,217 noncanonical microproteins in the human brain. However, the implied reliability for all 3,217 targets is likely overstated even with a strict FDR cutoff in their analysis. Extended Data Fig. 1 reveals that noncanonical microproteins are significantly more likely to be identified by a single unique peptide compared to canonical proteins. These are known to carry a higher risk of being false positives. The distinction between a chemically detectable peptide and a biologically stable and functional protein is not fully addressed for the bulk of the atlas. The authors should consider stratifying the atlas into high-confidence (multi-peptide) and candidate (single-peptide) tiers. They should explicitly state the number and percentage of microproteins supported by ≥2 unique peptides versus those supported by only 1.
2) The immunofluorescence validation (e.g., Fig. 1J) should have statistical quantification of colocalization or expression

levels.
3) The authors claim that microproteins can be regulated independently of their canonical parent genes. However, the DE analysis relies on short-read RNA-seq (STAR/featureCounts) to quantify specific isoforms. Deconvoluting the expression of a dORF-containing isoform from the canonical isoform using short reads is prone to errors. The long-read sequencing data would be better for quantification, but has a very small sample size. To substantiate the claim of independent regulation, the authors should validate the isoform-specific expression changes for key examples (like EMC10) using qPCR using a larger sample size.
4) The authors claim that AD-associated microproteins are enriched in excitatory neurons and downregulated in glia. The phrasing of their claim implies that authors assume that the microproteins follow the gene's bulk cell-type specific expression patterns, which contradicts the authors' major claim that microproteins can be uncoupled from canonical gene regulation. The data in figure 3 more so supports that only the genes encoding them are. "Enrichment in excitatory neurons" should be rephrased to something like "Mapping to genes associated with excitatory neurons." If neurons simply make more RNA species in general they will naturally have more "candidate genes" for microproteins. This doesn't mean the microproteins are biologically specific to neurons. To demonstrate actual cell-type specificity, the authors should provide validation for candidates using immunofluorescence co-staining (e.g. staining for microprotein and excitatory neurons markers) or RNAscope targeting the smORF sequence.
5) The authors claim that there are >5,000 peptide spectrum matches for ACTBP11. This is supported by MS, long-read RNA seq and in vitro overexpression to look at protein stability. If a SNP in the abundant canonical protein mimics the pseudogene sequence, the entire identification could be an artifact. The authors must provide a visual showing multiple sequence alignment of ACTBP11 versus ACTB (and other actin paralogs) in the supplement. This alignment should highlight the tryptic peptide sequences identified by MS. In Figure 4 this is done with a few, but they should compare against SNP databases.
6) In Figure 5, the authors report that actin-related pseudogenes are actively translated. The data supporting this shows that overexpressed pseudogenes form stable proteins. The authors should consider endogenous knockdown strategies. While challenging due to sequence homology, targeting unique UTRs or specific junction sites using shRNAs or ASOs would provide proof that the endogenous pseudogene product is functional, rather than just the overexpressed construct.
7) The paper highlights the phenomenon of independent regulation, but offers no mechanistic explanation. Is it due to specific RNA-binding proteins or distinct promoter usage? The authors should perform bioinformatic motif analyses on the regulatory regions (UTRs, promoters) of the independently regulated transcripts. Identifying enriched motifs would provide a testable hypothesis for the mechanism of this uncoupling.
8) Validating the differential expression of key microproteins in an independent patient cohort or brain region using targeted MS would bolster the findings.
9) Lastly, increasing the sample size for the long-read transcriptomic analysis would improve statistical power for the pseudogene findings.

(Remarks on code availability)

Reviewer #4

(Remarks to the Author)
I co-reviewed this manuscript with one of the reviewers who provided the listed reports. This is part of the Nature Aging initiative to facilitate training in peer review and to provide appropriate recognition for Early Career Researchers who co-review manuscripts.

(Remarks on code availability)
The code is sufficiently detailed and includes the necessary instructions for the community to reproduce the results.

Reviewer #6

(Remarks to the Author)
This study by Miller et al. presents a microprotein atlas describing previously uncharacterized microproteins found via proteogenomic approaches after sampling of postmortem dorsolateral prefrontal cortexes from patients with and without Alzheimer’s disease. These proteins were identified via data independent acquisition proteomic approaches of the same dorsolateral prefrontal cortex samples, via a microprotein-proxy approach generating a set of Swiss-Prot analog microproteins based on physicochemical characteristics, and by ribosome profiling machine learning analysis (ShortStop). Transcript and peptide level changes of several microproteins were reported to correlate with Alzheimer’s disease. Lastly, the study describes a 63 amino acid mitochondrial microprotein encoded by a uORF at the MKKS locus (uORF1-MKKS) which exhibits Alzheimer’s-associated changes.

Alzheimer’s disease is known to impact the proteome and thus one would expect that proteins associated with Alzheimer’s disease would be extensively studied. However, due to the noted challenges associated with microprotein detection, many have been absent from reference databases and are thus of interest. Nevertheless, there are several interrelated issues with the current study.

1. Broadly, much of this paper is dense and a significant portion of the text describes lists, which provide limited insights, though the atlas will be a valuable resource. Further characterization of uORF1-MKKS might provide more understanding of microprotein contribution to Alzheimer’s disease.

2. “noncanonical”, which is just being used to refer to proteins that have not previously been detected, is not the best terminology (grouping alt-ORF, uORF, oORF and dORF, some without definition). The authors should be more precise. Why not use terminology “used by other reports”?

3. Page 3: How are microproteins with significant homology with the canonical protein but with extended or truncated termini synthesized? How is the N-terminally extended short isoform of RAB3C lacking the canonical C-terminus synthesized? Are these in the same or different frames? Is there evidence for alternative splicing or translational slippage? More explanation needs to be provided here.

4. Page 5: The authors need to better explain what they mean by proxy for microproteins.

5. Page 9: “When compared against the entire in silico protein database, although the smORF shared homology…” is unclear.

6. Approaches and abbreviations should be better defined for a general reader, who, for example, may not know the difference between DDA and DIA.

7. The authors need to be more precise and list actual numbers or fold differences. For example, on Page 3, “Many of these smORF-encoded proteins…”, “Finally, many smORFs are encoded…”, “microprotein was modestly downregulated…”, “isoform was modestly upregulated…”,

8. More care should be taken with the text and figures. For example:
--Lines 214 and 218 refer to Fig. 2f and 2g, respectively. These panels are not present in Fig. 2.
--Fig. 3b has a typographical error “Signifnificant”
--Lines 562-563: The following is not a complete sentence “The MKKS locus offers a striking example of how disease-relevant proteins without consideration of smORFs.”
--Line 604 “detection. In thi context, DIA” should be “In this context, DIA”

(Remarks on code availability)

Version 1:

Reviewer comments:

Reviewer #2

(Remarks to the Author)
The authors have addressed all of the Reviewers concerns, highlighting some of the study limitations in the text and significantly improving the manuscript. The adoption of the new 4-tier categorization based on spectral quality is an important improvement worth mentioning.
While the current version of the manuscript is already fit for publication, I would still suggest a few minor changes. These changes mostly intend to prevent an overestimation of their data, as the authors themselves acknowledge that large-scale proteomics studies are prone to false positives (Line 200).

Content suggestions:
Abstract – line 20: the authors claim to provide proteomic evidence of 4321 microproteins. > 450 proteins with Insufficient and > 350 proteins with Weak proteomic evidence according to their new categorization inflate this number. I suggest rewriting it to indicate these are protein candidates only.
Similarly, in line 125, we advise against claiming that all of these 4321 microproteins are “high-confidence identifications” based on FDR alone. The follow-up analysis show that only approximately 1000 are Strong identifications. The authors need to set a line between identifications and high-confidence identifications.
Methods – line 1013: the procedure for determining the spectral angle needs to clarified. A reference on the criteria for assigning the evidence labels is needed, or the authors need to explain why those values were chosen.

Textual corrections:
Line 55: please use a cross-reference to clarify that dORF means downstream ORF.
Figure 7 – Legend skips subpanel F.

(Remarks on code availability)
Available code is sufficient to understand the dataset and reanalyze it.

Reviewer #3

(Remarks to the Author)

I thank the authors for their thorough revision. The manuscript addresses the concerns I raised in the first round, and several of the changes go beyond what the rebuttal letter alone communicated. The revision substantially strengthens the paper through PROSIT-based confidence grading, MSBB cross-cohort replication, new mechanistic motif analyses and deepened MKKS characterization. The atlas will be a valuable resource for the community.

(Remarks on code availability)

Reviewer #4

(Remarks to the Author)
I co-reviewed this manuscript with one of the reviewers who provided the listed reports. This is part of the Nature Aging initiative to facilitate training in peer review and to provide appropriate recognition for Early Career Researchers who co-review manuscripts.

(Remarks on code availability)

Reviewer #6

(Remarks to the Author)
We appreciate the substantive effort the authors put into significantly revising their manuscript, which has improved as a result. Some limited issues that are still outstanding.

1. Accessibility to a broad audience:
--dORF is first used on line 56 in Mai section, following the use of downstream ORF on line 55. dORF is only explicitly defined as “downstream ORF” in the Results section on line 131.
-- The consistent use of “MS” (first used line 89) to mean mass spec is never defined as such and could prove confusing at first sight for someone coming from a field of with another abbreviation for MS.
--CDS is first defined as coding sequence on line 1267 but is used earlier throughout the manuscript.
--ROSMAP should be defined at first use.
--DLPFC (dorsolateral prefrontal cortex) may not be familiar to all the readers of Nature Aging (but is not defined anywhere) perhaps at use on line 107.
--Overuse of shorthand nomenclature may detract from the clarity of the manuscript.

2. Precision about numbers:
By what metric have the authors concluded that most smORFs are within genes already annotated?
On line 62, “Transcriptional evidence alone is insufficient to determine which smORFs are active because most are embedded within genes already annotated to encode canonical proteins (main ORFs), residing upstream, within, or downstream of the annotated coding sequence.” This sentence also is a little confusing as the authors use the term ‘within’ twice in two differing ways: within genes already annotated… residing upstream, within, or downstream of the annotated coding sequence.

3. Care with figure call outs and figures:
--Figure 7:
-Line 546 panel G is described as Proteomics analysis but displays basal OCR timecourse.
-Line 551 references panel L. Panel L does not exist.
--Figure 1:
-The orange background block and the dotted lines above and below parent gene in Figure 1D are distracting and not needed.

(Remarks on code availability)

Open Access This Peer Review File is licensed under a Creative Commons Attribution 4.0 International License, which permits use, sharing, adaptation, distribution and reproduction in any medium or format, as long as you give appropriate credit to the original author(s) and the source, provide a link to the Creative Commons license, and indicate if changes were made.
In cases where reviewers are anonymous, credit should be given to 'Anonymous Referee' and the source.
The images or other third party material in this Peer Review File are included in the article's Creative Commons license, unless indicated otherwise in a credit line to the material. If material is not included in the article's Creative Commons license and your intended use is not permitted by statutory regulation or exceeds the permitted use, you will need to obtain permission directly from the copyright holder.
To view a copy of this license, visit https://creativecommons.org/licenses/by/4.0/

Response to Reviewers

We thank the reviewers for their thorough and constructive critiques. We are genuinely grateful for their engagement, which has elevated this manuscript well beyond its original form. We have made substantial changes in direct response, and they are summarized in the bullet points below. We additionally provide a point-by-point response to each reviewer's comments.

- **New Figure 2** introduces a four-tier Microprotein Grading System (Strong, Moderate, Weak, Insufficient) based on PROSIT spectral angle agreement and fragment-ion coverage for all detected microproteins. Figure 2B shows the full grade matrix with counts. Figure 2C shows spectral angle distributions confirming tier separation. Figure 2D breaks down grades by smORF type. Figure 2F reports the number of microproteins supported by one versus two or more unique peptides, cross-referenced by confidence grade. Figure 2G–H presents a triadic evidence profile, comprising Ribo-seq coverage, parent gene schematic, and a PROSIT mirror plot, for a representative microproteins.
- **New Extended Data Figure 1** shows manual blind grading validation by two independent reviewers, with Spearman correlations and inter-rater agreement comparable to previously published standards.
- **New Figure 4** presents CDS-level quantification of smORF–main ORF coupling across 4,326 pairs in ROSMAP, revealing a bimodal architecture (mean r = 0.97 for co-expressed pairs; mean r = 0.52 for decoupled pairs). Figure 4B–I shows Likelihood Ratio Test results across 502 differentially expressed smORF-containing transcripts, with dORFs showing the highest rate of independent regulation (48%). Figure 4M–O shows cross-cohort replication in the MSBB parahippocampal gyrus cohort (r = 0.81).
- **New Figure 5** presents mechanistic analyses of smORF–main ORF RNA expression. Figure 5A–B shows class-specific RBP motif enrichment at smORF splice junctions. Figure 5C identifies a predicted PTBP1 binding site at the MKKS exon 2–3 junction. Figure 5D–F shows the MKKS locus decorrelation profile and AD-associated changes.
- **New Extended Data Figure 5** shows HOMER promoter motif analysis across 3,631 smORF–main ORF pairs, demonstrating shared promoter architecture and CpG island co-occurrence.
- **New Figure 8** reconstructs the pseudogene section. Figure 8A shows Ribo-seq coverage at the ACTBP7 locus. Figure 8B shows 24-nucleotide k-mer unique mappability. Figure 8C–D shows dual PROSIT mirror plots for two ACTBP7-derived tryptic peptides. Figure 8E–I shows F-actin disruption upon ACTBP7-smORF overexpression.

- **New Extended Data Figure 7** shows nominally significant RNA downregulation of actin-related pseudogene transcripts in AD from long-read RNA-seq (moved from main figures; communicated as exploratory).
- **New Extended Data Figure 8** shows the multiple sequence alignment of ACTBP7 tryptic peptides against 342 ACTB missense variants, confirming sequence uniqueness of the detected peptides.
- **Revised Streamlit web application** now provides every spectrum and PROSIT mirror plot for individual download, full-dataset table sorting, log2 fold-change values in place of binary indicators, and corrected UTF-8 CSV export. https://brain-microprotein-atlas.streamlit.app/. The password is "revision" (all lower case).
- **The manuscript writing** has been significantly revised based on these new analyses. We have additionally added substantially more references, adjusted nomenclature to fit within community norms (i.e., "Reviewed" and "Unreviewed"; smORF type (uORF, iORF, dORF), and provided greater guidance on interpreting the results.

Reviewers' Comments:

Reviewer #1 (Remarks to the Author):

In this work, the authors present a multi-omics analysis of proteins below 150 aa by TMT-MS, DIA-MS and ribo-Seq in human brains. They complement and validate some of their findings using orthogonal methods such as immunofluorescence imaging, Western Blotting, RiboSeq and mitochondrial activity assays. The results are made available as a public atlas of microproteins in the context of aging and Alzheimer's disease. The majority of the data presented in the manuscript were previously published: The TMT-MS and ribo-Seq data were published previously and now re-analyzed. While the previous publication for this TMT-MS dataset did not focus on microproteins, the reprocessing of the data presented on this manuscript follows standard or previously published workflows. The ribo-Seq data was reanalyzed using ShortStop, a framework for microprotein discovery previously published by the authors. The provided code contains sufficient information to run it, including a list of requirements and readme files. The code availability on GitHub makes it compliant to good practices on data transparency.

The study is of broad interest due to the disease context in which microproteins are yet understudied, and as such would be suitable for publication in Nature Ageing. However, in the present form, the study contains a number of limitations that need to be addressed before further consideration for publication as outlined below:

Major Issues

Specifically, the statistical analyses carried out in this work require clarification. In line 131 and 134, a q < 0.2 is used as a significance threshold. If this q refers to a q-value from a multiple hypothesis testing, a threshold of 0.2 is unusually high, indicating that up to 20% of the values are false-positives. Similarly, the same q-value threshold was applied at Figure 1K to claim differential regulation of BCL3-oORF and RAB3C. Using a q-value threshold for significance of 0.2 is not appropriate and does not meet minimal standards for a statistical analysis.

Also, overall fold changes are relatively small (Figure 1G). We are surprised so many proteins passed the statistical significance threshold and assume this may be due to the high q value threshold (0.2), making the analysis relatively error-prone. The authors need to redo these analyses and apply a statistical test using thresholds that are accepted in the field (such as 0.05 or 0.01).

If the authors want to include a “low-fidelity” cohort of hits with high q value thresholds that would be ok, but these results need to be labeled as such and clearly separated from results that were acquired with commonly-used q value thresholds.

Response: All differential analyses have been completely re-processed under a dual-tier reporting framework. The results are found in Supplemental Table 2 and presented in a revised Figure 3. The “Significant” tier applies a conventional FDR < 0.05 threshold and is clearly distinguished throughout the manuscript and figures. Results that fall between FDR = 0.05 and FDR = 0.2 are retained as an explicitly labeled “Exploratory” tier, consistent with the reviewer’s suggestion, as seen in our Streamlit app. In the stringent model (microproteins detected in >50% of donors, which is the standard threshold for previous ROSMAP TMT reported studies), we identified 22 Unreviewed microproteins at FDR < 0.05. Expanding to the FDR < 0.2 without the 50% threshold (see page 9 response to review on “missingness” of each microprotein, which we simultaneously address here), exploratory tier across both models yields 243 unique Unreviewed microproteins (40 from the stringent model and 203 additional from the relaxed model), spanning all smORF classes (69 dORFs, 26 iORFs, 21 uORFs, 25 lncRNA-derived, and 16 psORFs).

The user can therefore be stringent with conventional FDR 0.05 filtering with >50% missing threshold or exploratory with relaxed parameters of FDR <0.2 and at least detection in one donor per condition.

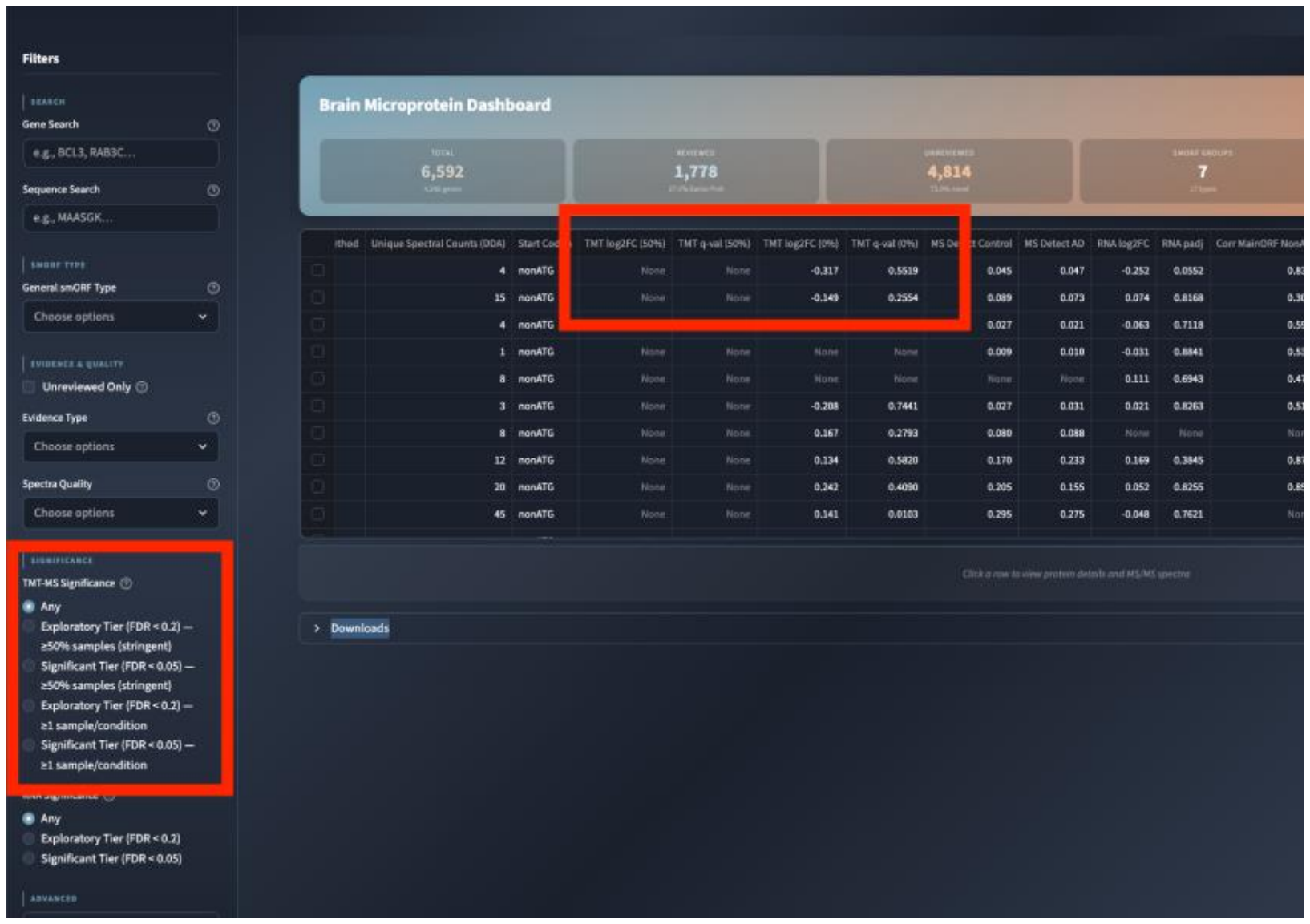

Furthermore, while the combined data present in the microprotein atlas will serve as a useful and important repository for future studies, the numbers stated in the manuscript include proteins supported by only one peptide and only one peptide-spectra-match (PSM). One-hit wonders are a known limitation in studies of small proteins as it is often not possible to detect more than one peptide for many of these proteins, making it hard to distinguish them from false-hits. The authors should consider approaches to manually curate and validate these identifications by e.g. only including one-hit-wonders with high-quality peptide spectra (i.e. with fragment spectra containing at least 5 consecutive b or y ions, as suggested in previous publications from this group, e. g. Slavoff et al., 2012). Alternatively, the authors could predict retention time and fragmentation patterns of the detected microprotein-derived peptides (both canonical and non-canonical) and evaluate the overlap between the data they acquired and the predicted spectra (using e.g. PROSIT by the Wilhelm lab).

Taken together, the claim that there is a hidden layer of thousands of noncanonical microproteins (Lines 67, 137 and 514) seems exaggerated as the majority of them could not be quantified nor are supported by more than 1 PSM, without any manual curation or validation. We also strongly recommended that spectra for proteins identified by a single peptide are made available either in the online atlas or as supplemental figures.

Response: We implemented the PROSIT-based approach. It underpins an entirely new Figure 2, which formalizes a 4-tier Microprotein Grading System (Insufficient, Weak, Moderate, Strong) based on two orthogonal criteria: (1) spectral angle agreement between observed and PROSIT-predicted MS/MS (threshold boundaries at 0.222 and 0.500) and (2) fragment-ion coverage (sparse <25%, partial 25–50%, majority >50%). We adopt the spectral angle nomenclature from the recently published Nature Communication (https://www.nature.com/articles/s41467-025-68002-x), and we go a

step further grading the peptide by ion coverage; Figure 2A reflects our system. Figure 2B shows the full grade matrix with counts for each combination of criteria. Figure 2C shows spectral angle distributions by confidence grade, confirming clear separation between tiers. We add a new Extended Data Fig. 1, in which we had two manual inspectors cross-validate our grading approach with significant Spearman correlation scores of ~0.6 and inter-rater of ~0.95, which is like that reported in the Nature Communication report. Figure 2D breaks down grades by smORF type (downstream ORF, TrEMBL/uORF, short-isoform, oORF, lncRNA, upstream ORF, psORF). We also directly quantify the structural basis for single-peptide identifications in new Figure 2E, which shows the theoretical tryptic peptide capacity for all Reviewed and Unreviewed microproteins. A substantial fraction of Unreviewed sequences yield only 1 tryptic peptide based on sequence length alone, confirming that single-peptide detection at this scale reflects a structural ceiling imposed by protein size, not an analytical failure. Figure 2F cross-references this with observed confidence grades, showing that the majority of single-peptide identifications with Moderate or Strong PROSIT grades represent genuinely detectable proteins constrained by biology.

Every single spectrum is now publicly available in our new Streamlit web application and can be bulk downloaded at https://huggingface.co/datasets/brmiller/brain-microprotein-atlas/tree/main/mirror_plots.

In addition, representative mirror plots for key reviewed microproteins (BCL3-smORF, RAB3C) are shown directly in Figure 3H-I, and Figure 2G shows a representative PROSIT mirror plot for the NYAP1 uoORF, illustrating the full triadic evidence profile, comprising Ribo-seq coverage, parent gene schematic, and PROSIT-validated MS/MS, for a microprotein assigned a Strong grade.

H

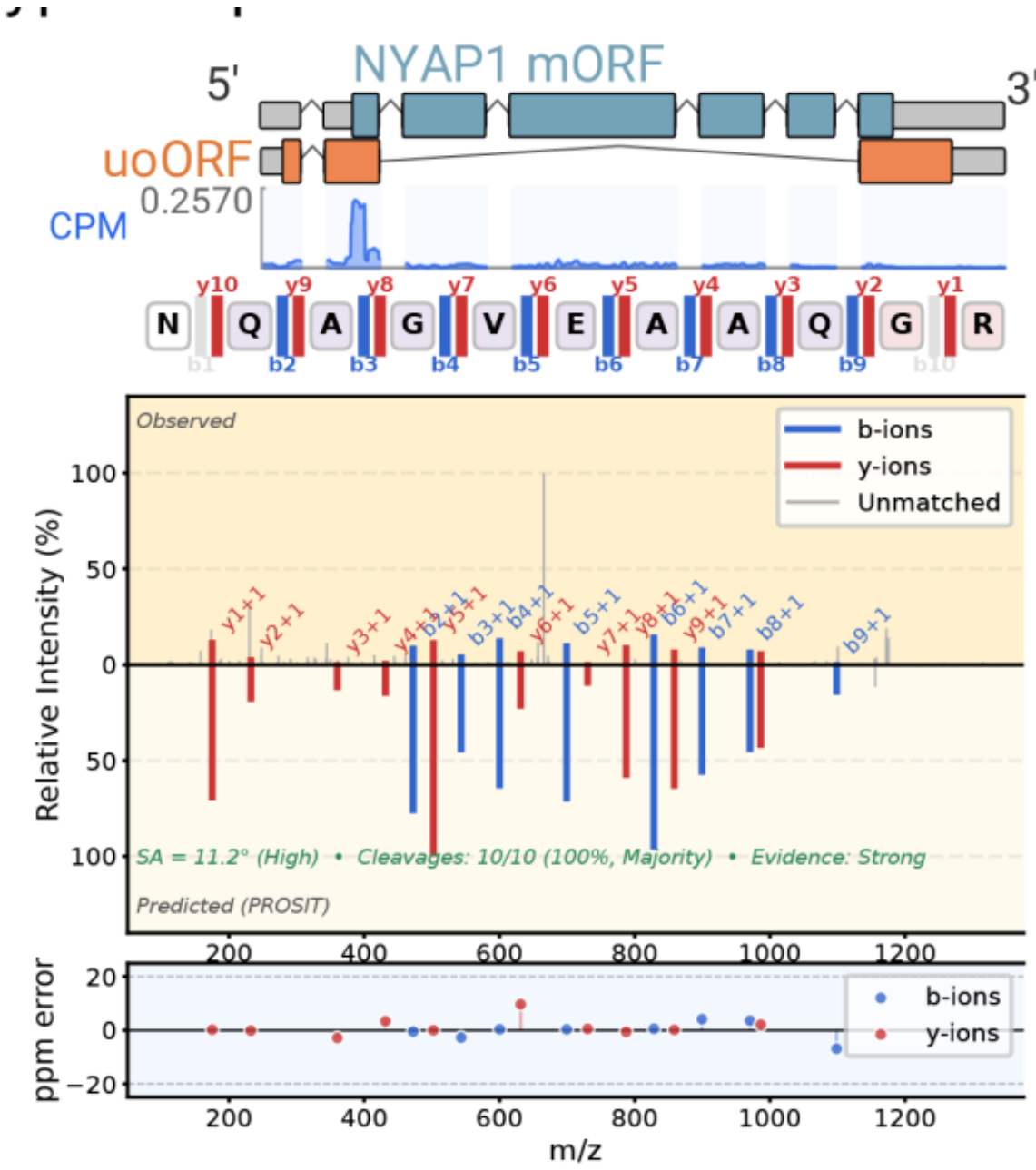

The presentation of the data in the current manuscript needs to be improved, as indicated below. While most figures are well-structured, a number of figure panels are wrongly cross-referenced or missing, figure legends need to be improved to describe each panel, and larger font sizes are needed in many figure panels to improve readability. In general, we also ask for higher-resolution figures to be uploaded as for example the spectra in Figure 1H cannot be assessed properly at this resolution. We also found throughout the text a few sentences that are missing a reference and a few references that cite the wrong publication (see below).

Proteomics data need to be more transparent in this publication. While a PRIDE identifier was made available throughout the review process, the online Atlas should also contain the links to the raw data.

Response: All figure cross-references have been verified and corrected throughout the manuscript. Resolution and font sizes have been increased across all panels, including the MS/MS spectra. The spectral validation approach has been overhauled. The original low-resolution spectra in the original document has been replaced by a new “triadic evidence” profile containing Ribo-seq tracks, exon architecture, MS/MS, and a mirror plot showing PROSIT prediction with grade. We consider this display and presentation a significant overhaul to how microproteins are communicated. All figure legends have been expanded to describe each panel explicitly. Missing references have been added and incorrect references corrected (see specific line-by-line responses below).

We’ve added four badges/links to the top of our GitHub repo: Zenodo containing all large files, the live dashboard streamlit app, Hugging Face deposited ~12,000 individual figures of high resolution panels for MS/MS PROSIT mirrors plots, co-expression

profiles, and transcript architecture cartoons for every microprotein, and the PRIDE accession as requested.

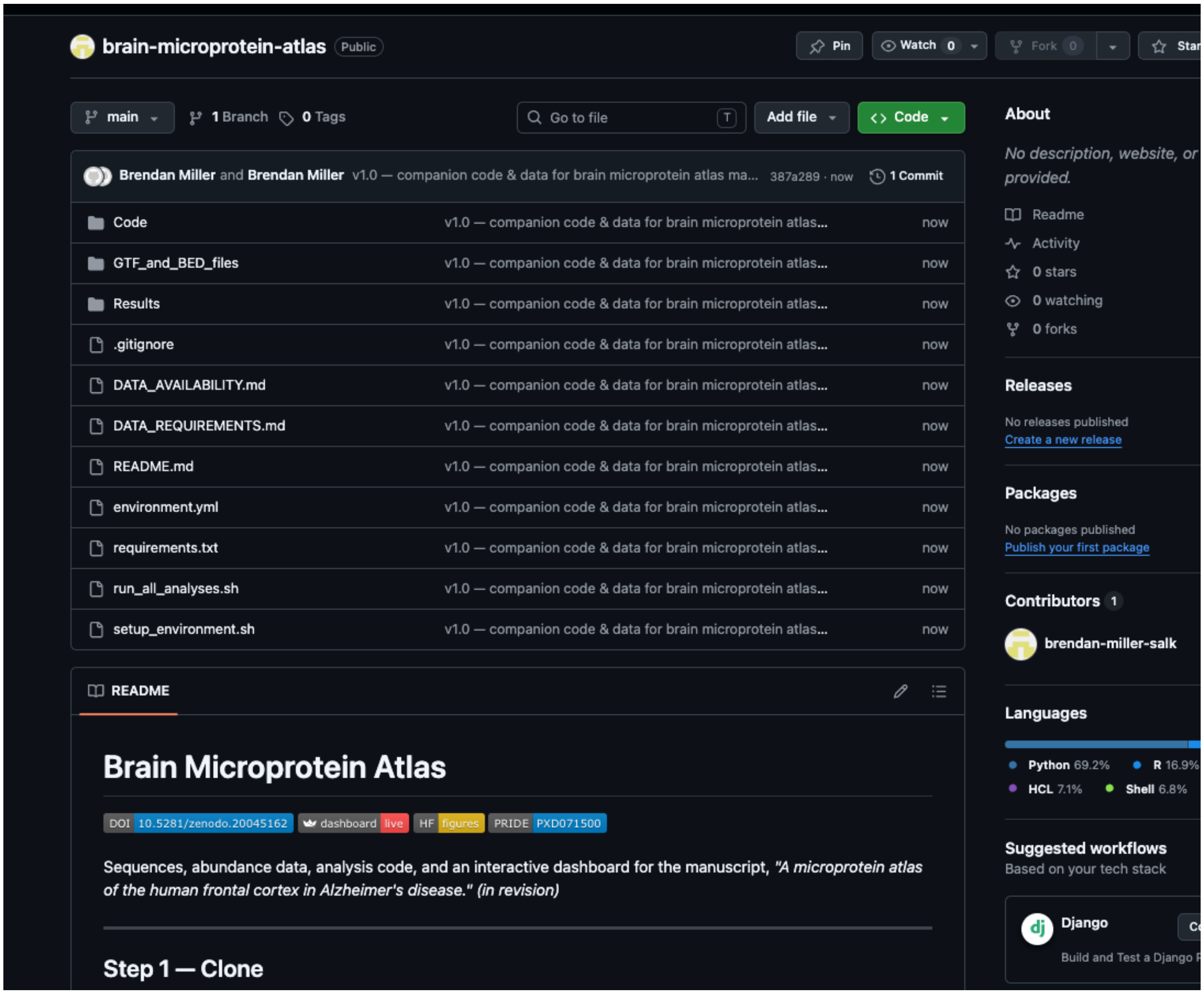

Minor issues

Line 38 – reference missing

Response: Reference added.

Line 44 – The authors need to reference their definition of microproteins as "peptides shorter than 150 amino acids". First, it is important to highlight that "proteins" would be a better term for this sentence instead of "peptide" (see PMID 24606146). Furthermore, most publications in the field of microproteins consider the threshold to be 100 amino acids.

Response: Corrected. 'Proteins' is now used. The term "peptides" is used with explicit mention to "tryptic peptide" to communicate the products detected through MS. The 100-150 amino acid boundary is indeed arbitrary. However, the TransCODE consortium

(a working group annotating smORFs) extends this boundary to 150 amino acids (https://www.nature.com/articles/s41586-026-10459-x#Sec52). We previously used the TransCODE Ribo-seq catalogue of smORFs to train our published machine learning model ShortStop: https://pubmed.ncbi.nlm.nih.gov/40756675/. We agree that nomenclature and boundaries are important, and so we devote a paragraph in the revised Discussion and as well as in the Technical Limitations. As the field continues to mature, the "microprotein" nomenclature may accordingly evolve.

Line 90 – Reference seems to be incorrect. Reference 30 (Bennett et al. 2018) does not contain any information about the TMT dataset.

Response: Corrected.

Line 92 – The fact that canonical microproteins generate more tryptic peptides and are generally longer is not surprising considered that the limitations in microprotein detection by MS are mostly observed for proteins < 100 aa, and are strikingly high for proteins < 50 aa. This is directly in line with our comment re. line 44.

Response: Agreed. We now directly demonstrate this in new Figure 2E, which plots the theoretical tryptic peptide capacity for all Reviewed and Unreviewed microproteins. This shows that single-peptide detection is mathematically expected for a substantial fraction of proteins in this size range. It is a consequence of limited tryptic space. Figure 2F then cross-references this with the observed peptide support by confidence tier.

Line 102 and Fig 1C – the horizontal spread around 0 on the violin plot for noncanonical proteins is much higher than for canonical proteins. This might indicate that some of them are not expressed at all. It would be interesting to see a correlation between transcriptomics data and MS-intensity / number of peptides/PSMs (shown similarly in Fig 1D). This might help filter out false-identifications.

Response: Agreed. We have carried out this analysis in new Fig. 1G. We show a modest Spearman correlation of ~0.3 for Reviewed microproteins between transcripts/spectral counts and a significantly lower correlation of 0.06 for Unreviewed microproteins.

Line 108-109 – The sentence does not read well. Perhaps the authors intended: "... coding sequences with different coding frames..."

Response: We have rewritten this passage and the surrounding paragraph to improve flow and clarity. Several other passages flagged in this and subsequent comments have also been revised.

Line 128 – We recommend reviewing and clarifying figure legends. Is Extended Data Fig 2D distinguishing canonical and noncanonical microproteins? While it feels intuitive

given the consistent colour choices throughout the text, an actual clarification in the legends would be recommended.

Response: We have edited all figure legends to improve clarity.

Line 129 – It would be interesting to see an overlap between canonical and noncanonical microproteins in Figure 1G. We would also appreciate a comment from the authors on the observed fold-changes. -0.2 to +0.2 log2-fold changes seem very small. The spread of the volcano-plot in Figure 1G is also misleading, the width makes the fold-changes seem much bigger than they actually are.

Response: We direct the reviewer to Extended Data Fig. 4 for all proteins observed in a matched volcano. We highlight that Extended Data Fig. 4 shows the viability of our pipeline because we reproduced the results reported by Johnston et al., 2022, which used the same ROSMAP dataset. Extended Data Fig. 4 also shows most proteins fall between -0.2 to 0.2 fold changes. This is to be expected and has been discussed in large-scale human proteomics studies. The effect sizes are best interpreted as directional disease associations rather than high-magnitude abundance shifts due to heterogeneity of bulk-processed tissues and covariate structures of post-mortem donors.

Line 132 – Did the authors investigate why only 4.6% of the noncanonical microproteins (according to Extended Fig 2D) were found in >50% of the samples? This number is low compared to 37.6% for canonical microproteins. Are there any group of microproteins found consistently in only one group? Also, these numbers may indicate that the majority of the noncanonical microproteins may be false-positive hits.

Response: We now directly address this with a degree-of-missingness analysis in Extended Data Fig 3D, and each microproteins contains a “missingness rate” by disease state and is accessible in new Supplemental Table 3 and within our Streamlit app, as well as visualized in new Fig. 3. These can further be filtered by our new PROSIT-based grading scale, which reveals a significant number of low-quantified proteins with moderate to strong grades. As discussed, proteins with only 1 theoretical tryptic peptide are inherently harder to quantify across large cohorts, independent of their biological reality. In essence, the user can download all microproteins based on any threshold and confidence rigor based on their questions.

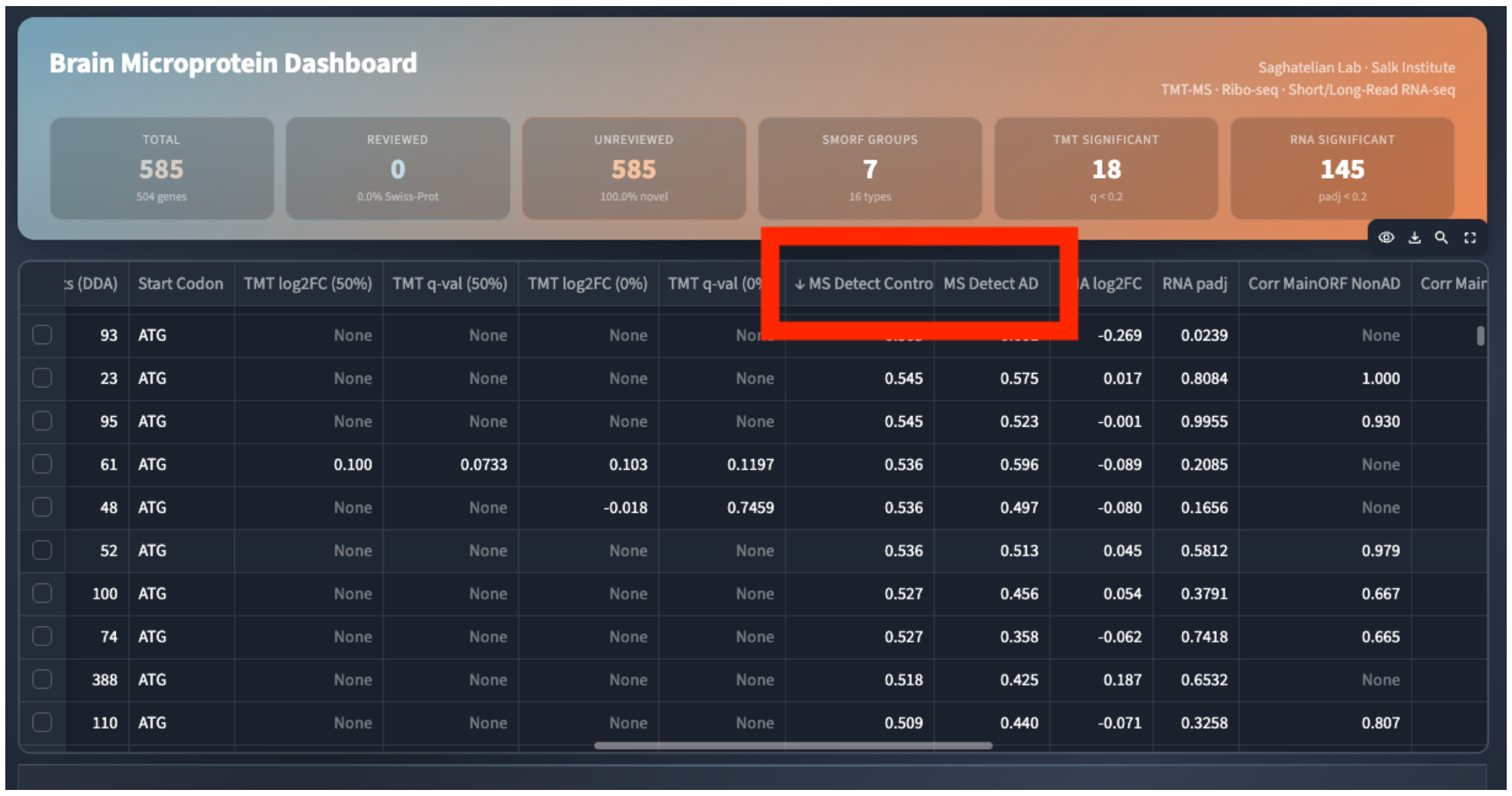

:s (DDA)	Start Codon	TMT log2FC (50%)	TMT q-val (50%)	TMT log2FC (0%)	TMT q-val (0%	↓ MS Detect Contro	MS Detect AD	A log2FC	RNA padj	Corr MainORF NonAD	Corr Mair
93	ATG	None	None	None	Nor	[illegible]	[illegible]	-0.269	0.0239	None	
23	ATG	None	None	None	None	0.545	0.575	0.017	0.8084	1.000	
95	ATG	None	None	None	None	0.545	0.523	-0.001	0.9955	0.930	
61	ATG	0.100	0.0733	0.103	0.1197	0.536	0.596	-0.089	0.2085	None	
48	ATG	None	None	-0.018	0.7459	0.536	0.497	-0.080	0.1656	None	
52	ATG	None	None	None	None	0.536	0.513	0.045	0.5812	0.979	
100	ATG	None	None	None	None	0.527	0.456	0.054	0.3791	0.667	
74	ATG	None	None	None	None	0.527	0.358	-0.062	0.7418	0.665	
388	ATG	None	None	None	None	0.518	0.425	0.187	0.6532	None	
110	ATG	None	None	None	None	0.509	0.440	-0.071	0.3258	0.807	

Figure 1A – For clarity, the authors should state the meaning of Asym-AD and Sym-AD. While intuitive, it is important that these abbreviations are noted in the figure legend.

Response: Definitions are explicitly stated in the results and significantly expanded on in the Methods Within the text we note that this is not our diagnostic criteria; it is the harmonized criteria reported by Johnston et al., 2022, which analyzed the same ROSMAP post-mortem data, whose results we replicated

Figure 1H – The spectra would be more meaningful if added to the extended figures in larger sizes. In this panel, they are simply too small to be properly interpreted and the axis labels are unreadable.

Response: The spectral presentation has been completely overhauled. Every single spectra plot can be downloaded in vectorized format from our streamlit app as well as here: https://huggingface.co/datasets/brmiller/brain-microprotein-atlas/tree/main/mirror_plots. They are 300 DPI and vectorized images.

Line 167-169 – Several reviews are available in literature on the difficulties of detecting small and microproteins by MS. It would be relevant to cite at least one.

Response: Instead of one we have cited multiple publications for microprotein MS detection challenges and have dedicated new text to these challenges throughout the text, particularly in the Discussion.

Line 172 (and other occurences) – The authors should reference here the original publication from which the Ribo-Seq data comes from.

Response: Original Ribo-seq publication cited at first mention and all subsequent occurrences.

Line 173 - if this homology independent algorithm is ShortStop, this should be noted here, instead of in line 183. The initial sentences of this paragraph and the next one seem repetitive.

Response: ShortStop is now named at first mention.

Line 182 – It would be interesting to visualize the number of predicted tryptic peptides for these 1607 proteins in Extended Figure 1. In our experience, it is not unusual to find microproteins with 0 tryptic cleavage sites.

Response: We have done this in new Figure 2E-F. Thank you for the suggestion.

Line 182 – The entire paragraph is confusing. After multiple times reading it, it remains unclear if the panels in Figure 2 are wrongly labelled or if the cross-references in the text are referring to the wrong panels. In addition to checking the cross-references, we suggest an improvement in the sentence's clarity.

Response: We apologize for the confusion. We have rewritten this and cross-referenced the panels to avoid confusion in our resubmission.

Line 195 – Do the authors mean "Undetected microproteins may fall outside..."? Could this indicate that some of these are simply false identifications? If they have low ribosome occupancy and no MS-data, there is not enough support for their expression. Line 198 – As above, is this referring to Fig 2C?

Response: We have corrected this for clarity.

Line 204 – typo in analysis

Response: We have now added two references.

Line 207 – the authors should reference at least one of the studies showing that many microproteins originate de novo and are less conserved.

Response: All figure panel cross-references verified and corrected throughout.

Line 212 and 217 – Panels F and G are missing, or wrongly cross-referenced. Line 249 – The authors claim that at the peptide level, microproteins that map to neuronal-associative genes were lower abundance than canonical Swiss-Prot proteins (Fig 3C). This however was showed for the overall microproteome in Figure 1D. Did the authors compared the noncanonical neuronal-associative microproteins from Fig 3C, to a background of all other noncanonical microproteins to see if there was a difference? Line 249 – Same as above – Figure 3C seem to indicate that non canonical small proteins have larger Log10PSM values than SwissProt for most cell types. This is conflicting to the claim in the text. Line 251 – For astrocytes, the Log2-FC seem too heterogeneous to support the claim that they are generally upregulated in AD.

Response: We have removed this analysis from the revision, in line with similar comments from Reviewer 3. We did not communicate this result as intended, and in its original form we now understand it is prone to misinterpretation. Therefore, in the revised version, we show 4 specific examples of how to implement microproteins with previously acquired summary statistics (i.e., SST, MDK, BCL3, and RAB3C). We clearly state this is meant to support functional studies and to motivate future scRNA-seq studies that directly quantify smORFs.

Line 280 – reference missing.

Response: Reference added.

Line 284 – reference to RP3 missing.

Response: Reference added.

Fig 6A – We recommend including an y-axis for the ribo-seq data to enable to visualization of the signal intensity.

Response: Done. All Ribo-Seq axes have been appropriately scaled and included y-axes.

Line 384 – can the authors comment on the biological significance of a -0.06 log2-fold change in the context of the project? While significant, this difference seem way too small to be meaningful.

Response: The text has been revised to contextualize this within the bulk-tissue, large-cohort TMT-MS framework where effect size attenuation is expected, and to note that statistical significance in a ~500-brain cohort does not necessarily imply large individual-level effect sizes.

Line 629 – Paragraph is duplicated.

Response: Duplicate paragraph removed.

Line 672 – Please highlight the number of sequences present in the downloaded UniProt proteome, and the date in which it was downloaded.

Response: UniProt proteome sequence count and download date added to the Methods section.

Line 708 – A reference for the RP3 pipeline is missing.

Response: Reference added.

A few comments on the Streamlit App. These are not critical but might be considered as improvement points for next updates on the platform.

- When downloading a CSV, the emojis used in the web platform are downloaded as unformatted code, which makes sorting/filtering in excel or R a bit more complicated (e.g. “No” is viewed in excel as “âŒ No”.

- In the general noncanonical explorer, it would be interesting to see the fold-changes observed in TMT-MS / RNA instead of simply Yes/No.

- In the detailed individual analysis hub, when you have a large number of entries in the table, but are displaying less rows (e.g. showing 4,321 entries but displaying only 50 rows), any attempt to sort the table by a column only affects the 50 visible rows. For example, if I sort by TMT_log2foldchanges, it sorts only for those 50 values and it remains unclear how it looks for the rest.

Response: All three issues resolved in the re-engineered Streamlit application. (1) Emoji encoding in CSV downloads corrected. All columns export as clean UTF-8 text. (2) Table sorting now operates on the full dataset, not only the visible rows. (3) Fold-change values (log2FC from TMT-MS and RNA-seq) are now displayed directly in the explorer tables rather than as binary Yes/No indicators.

Reviewer #1 (Remarks on code availability):

The provided code contains sufficient information to run it, including a list of requirements and readme files. The code availability on GitHub makes it compliant to good practices on data transparency.

Reviewer #2 (Remarks to the Author):

I co-reviewed this manuscript with one of the reviewers who provided the listed reports. This is part of the Nature Aging initiative to facilitate training in peer review and to provide appropriate recognition for Early Career Researchers who co-review manuscripts.

Reviewer #3 (Remarks to the Author):

This manuscript presents an atlas of unannotated microproteins in the human brain, utilizing a multi-omics workflow to characterize these proteins in the context of Alzheimer's disease (AD). The authors integrated long-read transcriptomics, Ribo-Seq, and quantitative proteomics (TMT-MS) across more than 480 dorsal lateral prefrontal cortex samples to identify 3,217 noncanonical microproteins absent from standard reference databases. The authors find that several microproteins that are differentially expressed in AD are regulated independently of their canonical parent genes (e.g., EMC10-dORF, UBA1-uORF), indicating disease-specific regulatory mechanisms. The

authors report that 215 loci annotated as "pseudogenes" are translated in the human brain and that there is coordinated repression of 13 actin-related pseudogenes in AD.

The most rigorously supported section of this study is in figures 5 and 6, where the authors claim that uORF1 is the dominant product of the MKKS locus, localizes to mitochondria, and controls mitochondrial respiration. The authors demonstrate a clear distinction between the gene locus (MKKS) and its microproteins. They show that while MKKS is a canonical gene, its primary output in the brain is the noncanonical uORF1 microprotein. The use of the "PepCentric" meta-database to show the absence of canonical MKKS across 66,700 public experiments provides strong external validity. Additionally, the controls for the CRISPR experiments are strong and the 40-50% reduction in respiration is biologically significant. As a whole, this manuscript nicely integrates multiple techniques (long-read RNA-seq, TMT-MS, Ribo-Seq, DIA-MS). The data quality is high, with clear MS/MS spectra, and immunofluorescence imaging data provided. Furthermore, the reporting is transparent, methods are detailed, and data and analysis code have been made available via public repositories.

This study is the first large-scale atlas of noncanonical microproteins in the aged and AD human brain and it provides a robust workflow for discovering noncanonical proteins in complex human tissues. The results are of interest to neurobiologists, genomics and proteomics researchers. The manuscript can be further strengthened after addressing the following comments:

1) The core of the study is the creation of a “high-quality” atlas of 3,217 noncanonical microproteins in the human brain. However, the implied reliability for all 3,217 targets is likely overstated even with a strict FDR cutoff in their analysis. Extended Data Fig. 1 reveals that noncanonical microproteins are significantly more likely to be identified by a single unique peptide compared to canonical proteins. These are known to carry a higher risk of being false positives. The distinction between a chemically detectable peptide and a biologically stable and functional protein is not fully addressed for the bulk of the atlas. The authors should consider stratifying the atlas into high-confidence (multi-peptide) and candidate (single-peptide) tiers. They should explicitly state the number and percentage of microproteins supported by ≥2 unique peptides versus those supported by only 1.

Response: New Figure 2 is dedicated entirely to this stratification. We introduce a 4-tier Microprotein Grading System (Insufficient, Weak, Moderate, Strong) based on PROSIT spectral angle agreement and fragment-ion coverage. Figure 2B shows the full grade matrix with explicit counts at each tier. Figure 2D shows the grade breakdown by smORF type. Figure 2F directly reports the number of microproteins supported by 1 vs. ≥2 unique peptides, cross-referenced by confidence grade. Specifically, of 3,065 Unreviewed microproteins assessed (assigned to their highest-confidence peptide

grade), 995 were graded Strong (32.5%), 1,393 Moderate (45.4%), 313 Weak (10.2%), and 364 Insufficient (11.9%). All atlas entries are now labeled with their confidence grade in the manuscript, supplemental tables, and the Streamlit web application, enabling users to filter by their preferred confidence threshold.

2) The immunofluorescence validation (e.g., Fig. 1J) should have statistical quantification of colocalization or expression levels.

Response: Quantification has been added to the confocal microscopy data in revised Figure 3J. Specifically, we now quantify BCL3-smORF and RAB3C-smORF localization in HMC3 microglial cells overexpressing each construct, using Manders' Colocalization Coefficient (M1) to measure overlap with the DAPI nuclear channel. BCL3 shows near-complete nuclear overlap (M1 = 0.94), whereas RAB3C is excluded from the nucleus (M1 = 0.00), confirming distinct subcellular distributions.

3) The authors claim that microproteins can be regulated independently of their canonical parent genes. However, the DE analysis relies on short-read RNA-seq (STAR/featureCounts) to quantify specific isoforms. Deconvoluting the expression of a dORF-containing isoform from the canonical isoform using short reads is prone to errors. The long-read sequencing data would be better for quantification, but has a very small sample size. To substantiate the claim of independent regulation, the authors should validate the isoform-specific expression changes for key examples (like EMC10) using qPCR using a larger sample size.

Response: The reviewer's concern is valid and we address it directly. Our approach was specifically designed to sidestep isoform assignment. Rather than attributing reads to full transcript isoforms, we quantified expression by counting reads mapping exclusively to the coding sequence of each ORF, the smORF CDS and the main ORF CDS separately, using the FeatureCounts "cds" tag. This distinguishes transcript classes at the CDS level without requiring isoform deconvolution and makes no claim about which full-length isoforms carry the signal, an important methodological distinction we now make explicit in the revised manuscript.

We were unable to perform qPCR validation on the ROSMAP samples, as these post-mortem brain tissues are a finite and irreplaceable resource that cannot be reprocessed at scale. Instead, we performed a replication analysis in the independent MSBB cohort, which represents a separate brain region (parahippocampal gyrus), independent tissue processing, and an independent sequencing pipeline.

This CDS-level analysis, presented in new Figure 4, revealed a bimodal coupling architecture across 4,326 smORF–main ORF pairs in ROSMAP. Half of all pairs (n = 2,163; 50%) showed near-perfect co-expression (mean |r| = 0.97), consistent with the smORF and main ORF being carried on the same isoform. The remaining half (n =

2,163; 50%) were substantially decoupled (mean |r| = 0.52; global r = 0.69), with a 10th percentile of mean |r| = 0.25, indicating that a meaningful fraction of pairs are effectively uncorrelated across individuals. This decoupling signature replicated in the MSBB parahippocampal gyrus cohort (r = 0.81; Figure 4M–O), confirming it is not cohort- or region-specific.

To directly test whether this decoupling reflects statistical independence in disease, we applied a Likelihood Ratio Test across 502 smORF-containing transcripts differentially expressed in SymAD versus non-AD (RNA-seq FDR < 0.05). Of 390 testable pairs, 152 (39%) showed disease-associated changes that could not be accounted for by the main ORF. dORFs were disproportionately represented: 56 of 116 differentially expressed dORFs (48%, the highest rate of any smORF class) showed significant independence, and these independently regulated dORFs were enriched for Moderate-to-Strong PROSIT spectral grades, providing protein-level support for their biological relevance. Figure 4 directly shows smORF CDS versus main ORF CDS correlations across the full ROSMAP cohort, with selected dORF cases (EMC10, RNF41, GNAQ) shown with violin plots and corresponding graded MS/MS and Ribo-seq tracks (Figure 4J–L).

For every microprotein, we make its smORF expression and the corresponding gene's mORF publicly available for bulk download here: https://huggingface.co/datasets/brmiller/brain-microprotein-atlas/tree/main/expression_profiles. They can also be visualized with ease on our app, as shown below:

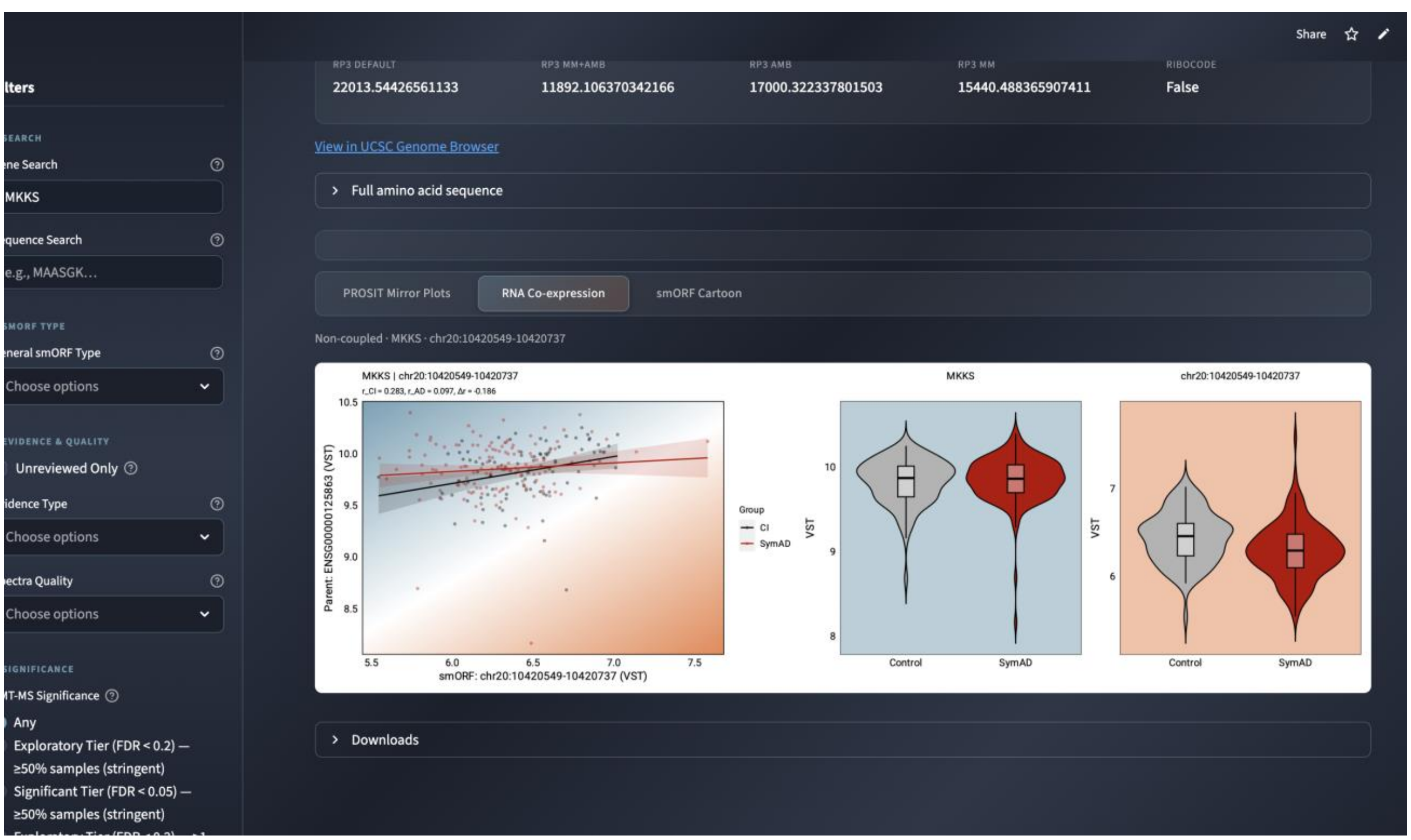

The observation that smORF-containing transcripts can be regulated independently of their host gene's main ORF is also supported by prior literature. Intrinsic polyadenylation of transcripts terminating in 5' UTR introns has been identified as a source of translated microproteins regulated independently of their full-length counterparts (Devaux et al., 2025). Similarly, a widespread class of capped 3' UTR-derived RNAs exists as autonomous fragments that do not co-localize with their parental mRNAs (Haberman et al., 2024; Malka et al., 2017). We expand on this in the Discussion.

We fully acknowledge that long-read native transcriptomics at comparable cohort scale would be the ideal future validation. We significantly expand on this in the limitations portion of the Discussion. We wish to bring attention to this de-correlation observation to motivate exactly the sort of study the reviewer suggests. An important technical consideration is that shorter transcripts, which disproportionately harbor smORFs, may fall below assembly quality thresholds during native transcriptome construction, potentially masking smORF-containing isoforms and requiring dedicated assembly strategies to recover.

4) The authors claim that AD-associated microproteins are enriched in excitatory neurons and downregulated in glia. The phrasing of their claim implies that authors assume that the microproteins follow the gene's bulk cell-type specific expression patterns, which contradicts the authors' major claim that microproteins can be uncoupled from canonical gene regulation. The data in figure 3 more so supports that only the genes encoding them are. "Enrichment in excitatory neurons" should be rephrased to something like "Mapping to genes associated with excitatory neurons." If neurons simply make more RNA species in general they will naturally have more "candidate genes" for microproteins. This doesn't mean the microproteins are biologically specific to neurons. To demonstrate actual cell-type specificity, the authors should provide validation for candidates using immunofluorescence co-staining (e.g. staining for microprotein and excitatory neurons markers) or RNAscope targeting the smORF sequence.

Response: We acknowledge that our original phrasing was imprecise and therefore we have removed the cell type–specific figure. Our intent was to illustrate, at a global level, genes encoding microproteins in a single-cell context. However, the way that we presented the original results unintentionally implied that the microproteins themselves are differentially regulated, an interpretation that is not supported and is confounded by technical limitations, as reflected in our bulk analysis (Fig. 4).

We have therefore reframed this analysis as targeted, single-gene examples. In the revised Fig. 3, we highlight SST and MDK (consistent with prior reports), as well as BCL3 and RAB3C, showing evidence of expression from these loci in microglia and related cell types.

We present these examples as illustrative rather than definitive, with the goal of motivating functional studies and encouraging more refined scRNA-seq approaches to capture smORF-containing transcripts.

5) The authors claim that there are >5,000 peptide spectrum matches for ACTBP11. This is supported by MS, long-read RNA seq and in vitro overexpression to look at protein stability. If a SNP in the abundant canonical protein mimics the pseudogene sequence, the entire identification could be an artifact. The authors must provide a visual showing multiple sequence alignment of ACTBP11 versus ACTB (and other actin paralogs) in the supplement. This alignment should highlight the tryptic peptide sequences identified by MS. In Figure 4 this is done with a few, but they should compare against SNP databases.

Response: This is a valid point, and we thank the reviewer for bringing it to our attention. We do exactly this analysis and present it in Extended Data Fig. 8. We show a multiple sequence alignment of ACTB sequences that are a product of missense mutations for ACTBP11 as well as ACTBP7 for rigor. Furthermore, new Figure 8 shows the k-mer alignment: 24-nucleotide unique k-mers are overlaid on the ACTBP7 locus sequences visually demonstrating which regions are uniquely mappable to ACTBP vs. shared/multi-map with other genomic loci. Additionally, Figure 8 now shows Ribo-Seq coverage at the ACTBP7 locus, providing orthogonal transcript-level evidence that these loci are actively translated as distinct entities. We also show dual high-resolution PROSIT mirror plots for two distinct ACTBP tryptic peptides, validating the spectral assignments with b and y ion coverage over the unique residues. With unique Ribo-Seq coverage and unique tryptic peptides, the data suggest endogenous production of microproteins from this locus, which warrants future studies on the endogenous levels, function, etc., as outlined by a recently published *Nature Communication* manuscript targeting the UBBP4 pseudogene and its 700-fold lower production compared to UBB.

6) In Figure 5, the authors report that actin-related pseudogenes are actively translated. The data supporting this shows that overexpressed pseudogenes form stable proteins. The authors should consider endogenous knockdown strategies. While challenging due to sequence homology, targeting unique UTRs or specific junction sites using shRNAs or ASOs would provide proof that the endogenous pseudogene product is functional, rather than just the overexpressed construct.

Response: We agree that endogenous knockdown would provide stronger evidence of function. For Micro-MKKS63, we successfully generated CRISPR-Cas9 knockout HMC3 microglia using two independent sgRNAs that disrupt the smORF, and the resulting mitochondrial respiration defects are shown in Figure 7. Micro-MKKS63 was in the top 1% of most abundant microproteins by PSM in our atlas, further motivating knockout because of its high abundance. However, for single-exonic pseudogenes, with

endogenous abundance unknown and with high sequence homology, designing specific shRNAs or ASOs is not currently reliable. Our 24-nt unique k-mer analysis (Fig. 8A) directly quantifies this limitation, showing that uniquely targetable regions are sparse and fragmented across the locus.

As an alternative, we now discuss CRISPR-based promoter-targeting strategies as a potential future direction that may improve locus specificity. In the absence of feasible knockdown approaches, we instead provide complementary new experimental evidence of biological activity, showing ACTBP7 and ACTBP11 overexpression alters F-actin organization (Fig. 8E–F; Extended Data Fig. 8), suggesting that the encoded microproteins are stable and capable of influencing cellular structure under these experimental conditions.

We emphasize that these overexpression experiments are not intended to reflect endogenous expression levels, but rather to test whether these pseudogene-derived products can exert measurable effects in vitro. We have clarified this point in the Discussion and now explicitly note that future studies will be needed to determine endogenous abundance, define cell-type specificity, and evaluate function under physiological conditions.

In addition, consistent with the reviewer's concern regarding statistical power, we have moved the nanopore-based differential expression analysis to the supplement and now present these results as nominal. We avoid making claims of essential or endogenous function for this class of microproteins. Instead, we position these findings as an example of how orthogonal omics data can guide hypothesis-driven studies of difficult-to-detect loci. Our results further suggest that pseudogene-derived microproteins represent a technically challenging yet potentially important class of molecules, and that this study presents a multi-omics framework to motivate resolving their expression and function.

7) The paper highlights the phenomenon of independent regulation, but offers no mechanistic explanation. Is it due to specific RNA-binding proteins or distinct promoter usage? The authors should perform bioinformatic motif analyses on the regulatory regions (UTRs, promoters) of the independently regulated transcripts. Identifying enriched motifs would provide a testable hypothesis for the mechanism of this uncoupling.

Response: We agree that identifying mechanisms underlying independent regulation is critical. In response, we have added a new Fig. 5 dedicated to this question, providing two complementary lines of analysis.

First, we tested whether smORF-containing transcripts arise from distinct transcription start sites. Using HOMER to analyze promoter regions across 3,631 matched smORF–

main ORF gene pairs, we find that the majority (~75%) of smORF-associated promoters share motif content with their corresponding main ORF promoters (Extended Data Fig. 5). This shared promoter architecture argues against widespread alternative transcription initiation as the primary driver of smORF–main ORF decoupling and instead points to post-transcriptional mechanisms.

Second, we performed FIMO-based motif analysis across splice junctions of smORF-containing transcripts. We observe distinct RNA-binding protein (RBP) motif enrichment compared to transcripts without evidence of microprotein production, with further stratification by smORF class (Fig. 5B). Short-isoform smORFs are enriched for GC-rich motifs associated with splicing regulators such as RBM4, PPRC1, and RBM8A. In contrast, downstream ORFs show enrichment for motifs linked to 3′ splice site recognition and polyadenylation (e.g., U2AF2, PABPC1, CPEB4), implicating 3′ end processing in the generation of these isoforms. These class-specific patterns support a model in which distinct post-transcriptional regulatory programs govern smORF-containing transcript production.

At the MKKS locus (Fig. 5C), we identify a predicted PTBP1 binding motif at the shared exon 2–3 splice junction present in both smORF-containing and full-length isoforms. Given PTBP1's established roles in alternative splicing and proximal polyadenylation, this provides a specific, testable hypothesis for how isoform balance could be regulated at this locus, consistent with the observed low correlation between smORF and main ORF transcripts (Pearson's $r = 0.15$).

We emphasize that these analyses are hypothesis-generating. We make the complete promoter and splicing results available in Supplemental Tables 8 and 9. We also generated cartoon figures that illustrate transcript architecture for every microprotein, to visualize exon/intron similarity and junction of smORFs and their canonical counterpart; they can be downloaded in bulk here: https://huggingface.co/datasets/brmiller/brain-microprotein-atlas/tree/main/smorf_cartoon_figures and are also available for easy viewing on our streamlit app. To further prioritize candidates, we rank motifs using a publicly available Dementia Score (Fig 5). This represents, to our knowledge, the first systematic RBP motif analysis of brain-derived smORF-containing transcripts and provides a framework for targeted mechanistic follow-up and provides testable mechanistic hypotheses for experimental follow-up.

8) Validating the differential expression of key microproteins in an independent patient cohort or brain region using targeted MS would bolster the findings.

Response: Cross-cohort replication has been performed and is presented in new Figure 4 (panels M–O). We replicated our differential expression signatures in the independent MSBB cohort (parahippocampal gyrus, a separate brain region from the

ROSMAP DLPFC; ROSMAP: SymAD n = 138, non-AD n = 87; MSBB: SymAD n = 132, non-AD n = 47). These comparable sample sizes provide meaningful statistical power for cross-cohort comparison. The smORF-level coupling profiles show robust replication (r = 0.81 between ROSMAP and MSBB). Individual microproteins including NCOA1 show concordant directional changes in both cohorts, with violin plots shown for both datasets in Figure 4N–O.

9) Lastly, increasing the sample size for the long-read transcriptomic analysis would improve statistical power for the pseudogene findings.

Response: We agree that a larger long-read cohort would strengthen the pseudogene findings. As previously mentioned, the pseudogene differential expression findings have been moved to supplementary in Extended Data Fig. 7 and communicated as explicitly nominally significant. We note this as a limitation in the revised manuscript and flag it as a priority for future work.

Reviewer #4 (Remarks to the Author):

I co-reviewed this manuscript with one of the reviewers who provided the listed reports. This is part of the Nature Aging initiative to facilitate training in peer review and to provide appropriate recognition for Early Career Researchers who co-review manuscripts.

Reviewer #4 (Remarks on code availability):

The code is sufficiently detailed and includes the necessary instructions for the community to reproduce the results.

Reviewer #5 (Remarks to the Author):

This study by Miller et al. presents a microprotein atlas describing previously uncharacterized microproteins found via proteogenomic approaches after sampling of postmortem dorsolateral prefrontal cortexes from patients with and without Alzheimer's disease. These proteins were identified via data independent acquisition proteomic approaches of the same dorsolateral prefrontal cortex samples, via a microprotein-proxy approach generating a set of Swiss-Prot analog microproteins based on physicochemical characteristics, and by ribosome profiling machine learning analysis (ShortStop). Transcript and peptide level changes of several microproteins were reported to correlate with Alzheimer's disease. Lastly, the study describes a 63 amino acid mitochondrial microprotein encoded by a uORF at the MKKS locus (uORF1-MKKS) which exhibits Alzheimer's-associated changes.

Alzheimer's disease is known to impact the proteome and thus one would expect that proteins associated with Alzheimer's disease would be extensively studied. However, due to the noted challenges associated with microprotein detection, many have been absent from reference databases and are thus of interest. Nevertheless, there are several interrelated issues with the current study.

1. Broadly, much of this paper is dense and a significant portion of the text describes lists, which provide limited insights, though the atlas will be a valuable resource. Further characterization of uORF1-MKKS might provide more understanding of microprotein contribution to Alzheimer's disease.

Response: The manuscript has been substantially revised for readability, with list-heavy passages rewritten as integrated prose throughout.

For the MKKS locus, the revision adds mechanistic depth. New analyses show the MKKS smORF-containing and main ORF transcripts show a Pearson r = 0.15 across ROSMAP samples, placing this locus in the bottom 10th percentile of all 4,326 smORF–main ORF pairs (Figure 4). HOMER promoter analysis confirmed shared promoter architecture between the two transcript classes, arguing against alternative transcription initiation, while RBP motif analysis at splice junctions identified a predicted PTBP1 binding site at the shared exon 2–3 junction as a candidate mechanism for selective smORF-only isoform production (Figure 5C). Co-expression network analysis anchored to the smORF CDS revealed robust mitochondrial gene ontology enrichment across disease states, whereas the same analysis anchored to the main ORF CDS showed no significant enrichment in either condition (Figure 6D–G). These analyses complement the CRISPR-based functional characterization presented in Figure 7.

2. "noncanonical", which is just being used to refer to proteins that have not previously been detected, is not the best terminology (grouping alt-ORF, uORF, oORF and dORF, some without definition). The authors should be more precise. Why not use terminology "used by other reports"?

Response: Terminology revised throughout. We now follow conventional nomenclature in this revision, where 'Reviewed' represents UniProt/Swiss-Prot reviewed sequences and 'Unreviewed' does not. Further, smORF subtype terminology (uORF, dORF, iORF, short-isoform, psORF) is explicitly defined at first use with reference to the conventions used in the broader microprotein field. The smORF type nomenclature borrows from the most recent standard in the field. We dedicate a nomenclature section in the discussion to discuss this and its future implications.

3. Page 3: How are microproteins with significant homology with the canonical protein but with extended or truncated termini synthesized? How is the N-terminally extended short isoform of RAB3C lacking the canonical C-terminus synthesized? Are these in the

same or different frames? Is there evidence for alternative splicing or translational slippage? More explanation needs to be provided here.

Response: In the revision we show unique promoter motifs for RAB3C in Supplemental Table 17. In new Figure 3, we also show unique Ribo-Seq translation across both the N-terminal extension smORF and the main ORF as well as unique intron/exon motifs at the first intron junction that may explain its termination. The revised text acknowledges this ambiguity explicitly and notes alternative expression or splicing as the most parsimonious explanation. In addition to RAB3C, every smORF and its ribosomal coverage is now made publicly available through our streamlit app, specifically in our UCSC genome browser hyperlinks: https://genome.ucsc.edu/s/brmiller/AD%20Dark%20Microproteome.

4. Page 5: The authors need to better explain what they mean by proxy for microproteins.

Response: We have re-written the text in a way that the word "proxy" is no longer necessary.

5. Page 9: "When compared against the entire in silico protein database, although the smORF shared homology…" is unclear.

Response: We have re-written the text and attempted to improved clarity.

6. Approaches and abbreviations should be better defined for a general reader, who, for example, may not know the difference between DDA and DIA.

Response: We see how the abbreviations are difficult to follow. We have since condensed and streamlined our evidence types. All abbreviations defined at first use: DDA (data-dependent acquisition), DIA (data-independent acquisition), TMT (tandem mass tag), smORF (small open reading frame), uORF (upstream ORF), dORF (downstream ORF), oORF (overlapping ORF), psORF (pseudogene-derived ORF), DLPFC (dorsolateral prefrontal cortex), CPM (counts per million), PSM (peptide-spectrum match). To make abbreviations less cumbersome, we have restructured figures to combine similar data types as necessary (i.e., collapsing all smORFs upstream of the main ORF simply as "Upstream ORF" and removing DIA/DDA separation from figures. This is meant to streamline interpretation. Precise definitions are now provided in Supplemental Table 18 for smORF types.

7. The authors need to be more precise and list actual numbers or fold differences. For example, on Page 3, "Many of these smORF-encoded proteins…", "Finally, many smORFs are encoded…", "microprotein was modestly downregulated…", "isoform was modestly upregulated…",

Response: All vague quantifiers replaced with specific numbers throughout. For example: ‘many’ replaced with actual counts; ‘modestly downregulated’ replaced with the specific log2 fold change and FDR value; ‘several’ replaced with the actual number of microproteins meeting each criterion.

8. More care should be taken with the text and figures. For example:

--Lines 214 and 218 refer to Fig. 2f and 2g, respectively. These panels are not present in Fig. 2.

--Fig. 3b has a typographical error “Signifnificant”

--Lines 562-563: The following is not a complete sentence “The MKKS locus offers a striking example of how disease-relevant proteins without consideration of smORFs.”

--Line 604 “detection. In thi context, DIA” should be “In this context, DIA”

Response: We have re-done every figure and revised the manuscript text for clarity.

Final Summary and Additional Context

During revision, Deutsch et al. (Nature, 2026) reported a TransCODE Consortium catalogue of ncORF-encoded microproteins with protein evidence derived primarily from cell-line ribosome profiling. We position our work as complementary, non-overlapping, and expansive. Our atlas is derived entirely from aged human frontal cortex tissue, with RNA and protein evidence drawn from the same donors and matched across clinical AD states. This same-donor, multi-omics design enabled frontal cortex-specific evidence of microproteins, disease-state analyses, smORF:mORF co-expression, mechanistic analyses, and Micro-MKKS63 microglial knockout that are unique to this manuscript. We have noted this distinction in the cover letter and have expanded significantly about the implications of our work in the Discussion. We are particularly excited because our manuscript positions our atlas as not only a discovery resource but an immediately actionable one for the neuroscience aging research community, at a time when microprotein research is gaining broad attention.

Again, we sincerely thank the reviewers for their thoughtful feedback and the clear effort they invested in strengthening this manuscript. Their comments substantially improved the study, and we are grateful for their careful evaluation and constructive suggestions.

Response to Reviewers

We thank the reviewers for helping us improve the quality of our manuscript and are thrilled that they share our enthusiasm with the presented atlas. The following is a point-by-point response to their request for minor changes. We have satisfied every one of these minor revisions.

Reviewer #1 (Remarks to the Author):

(not available for re-review)

Reviewer #2 (Remarks to the Author):

The authors have addressed all of the Reviewers concerns, highlighting some of the study limitations in the text and significantly improving the manuscript. The adoption of the new 4-tier categorization based on spectral quality is an important improvement worth mentioning.

While the current version of the manuscript is already fit for publication, I would still suggest a few minor changes. These changes mostly intend to prevent an overestimation of their data, as the authors themselves acknowledge that large-scale proteomics studies are prone to false positives (Line 200).

Response: We thank the reviewer, are happy to hear the current version is already fit for publication. We have accordingly addressed all minor changes.

Content suggestions:

Abstract – line 20: the authors claim to provide proteomic evidence of 4321 microproteins. > 450 proteins with Insufficient and > 350 proteins with Weak proteomic evidence according to their new categorization inflate this number. I suggest rewriting it to indicate these are protein candidates only.

Response: We have re-written the abstract exactly how the reviewer suggests. As the reviewer noted, approximately 1,000 Unreviewed microproteins of the 3,217 we report are "Strong" identifications, and therefore we have set this as the new line for high-confidence identifications. We write in the revised Abstract, “We identified 1,067 microproteins absent from reviewed UniProtKB entries with high-confidence spectral support.”

Similarly, in line 125, we advise against claiming that all of these 4321 microproteins are “high-confidence identifications” based on FDR alone. The follow-up analysis show that only approximately 1000 are Strong identifications. The authors need to set a line between identifications and high-confidence identifications.

Response: We have removed “high quality” and instead simply note 4,321 microproteins pass FDR <0.05 that were subject to PROSIT grading. Moreover, since the reviewer suggested drawing a line at the “approximately 1,000 microproteins,” we have since generated "Strong" only FASTA and GTF files for download in our GitHub repository and Streamlit app. Generating this file brought to our attention that the previous Figure 2 represented an earlier, stale data export illustrating 995 Strong microproteins rather than the correct 1,067. Revised Figure 2 corrects this visual discrepancy and does not affect any analyses or conclusions. These edits and new data files ensure that Strong-graded microproteins are easily accessible.

Methods – line 1013: the procedure for determining the spectral angle needs to clarified. A reference on the criteria for assigning the evidence labels is needed, or the authors need to explain why those values were chosen.

Response: The procedure has been clarified, and the reference has been added in both the Results and Discussion.

Textual corrections:

Line 55: please use a cross-reference to clarify that dORF means downstream ORF.

Response: We now use a cross-reference to clarify dORF means downstream ORF.

Figure 7 – Legend skips subpanel F.

Response: We have corrected Figure 7’s legend.

Reviewer #3 (Remarks to the Author):

I thank the authors for their thorough revision. The manuscript addresses the concerns I raised in the first round, and several of the changes go beyond what the rebuttal letter alone communicated. The revision substantially strengthens the paper through PROSIT-based confidence grading, MSBB cross-cohort replication, new mechanistic motif analyses and deepened MKKS characterization. The atlas will be a valuable resource for the community.

Response: We thank the reviewer for their suggestions and are happy to hear such enthusiasm.

Reviewer #4 (Remarks to the Author):

I co-reviewed this manuscript with one of the reviewers who provided the listed reports. This is part of the Nature Aging initiative to facilitate training in peer review and to provide appropriate recognition for Early Career Researchers who co-review manuscripts.

Reviewer #6 (Remarks to the Author):

We appreciate the substantive effort the authors put into significantly revising their manuscript, which has improved as a result. Some limited issues that are still outstanding.

1. Accessibility to a broad audience:

--dORF is first used on line 56 in Mai section, following the use of downstream ORF on line 55. dORF is only explicitly defined as “downstream ORF” in the Results section on line 131.

Response: We now use a cross-reference to clarify dORF means downstream ORF.

-- The consistent use of “MS” (first used line 89) to mean mass spec is never defined as such and could prove confusing at first sight for someone coming from a field of with another abbreviation for MS.

Response: MS is now defined at first use in line 50.

--CDS is first defined as coding sequence on line 1267 but is used earlier throughout the manuscript.

Response: CDS is now defined at first use in line 335.

--ROSMAP should be defined at first use.

Response: ROSMAP is now defined at first use in in line 339.

--DLPFC (dorsolateral prefrontal cortex) may not be familiar to all the readers of Nature Aging (but is not defined anywhere) perhaps at use on line 107.

Response: DLPFC is now defined at first use on line 106.

--Overuse of shorthand nomenclature may detract from the clarity of the manuscript.

Response: We have limited use of shorthand nomenclature.

2. Precision about numbers:

By what metric have the authors concluded that most smORFs are within genes already annotated?

Response: We have removed this phrase. We apologize for the imprecision. We simply state exactly where the smORFs are located in the Results section (line 101).

On line 62, “Transcriptional evidence alone is insufficient to determine which smORFs are active because most are embedded within genes already annotated to encode canonical proteins (main ORFs), residing upstream, within, or downstream of the annotated coding sequence.” This sentence also is a little confusing as the authors use

the term ‘within’ twice in two differing ways: within genes already annotated… residing upstream, within, or downstream of the annotated coding sequence.

Response: We apologize for the confusing sentence. We have removed it when shortening the introduction as part of the word count reduction requested by the Editorial team.

3. Care with figure call outs and figures:

--Figure 7:

-Line 546 panel G is described as Proteomics analysis but displays basal OCR timecourse.

-Line 551 references panel L. Panel L does not exist.

Response: We have corrected Figure 7’s legend.

--Figure 1:

-The orange background block and the dotted lines above and below parent gene in Figure 1D are distracting and not needed.

Response: We have removed the orange background block and the dotted lines.
